# The Role of Viral and Host MicroRNAs in the Aujeszky’s Disease Virus during the Infection Process

**DOI:** 10.1371/journal.pone.0086965

**Published:** 2014-01-24

**Authors:** Oriol Timoneda, Fernando Núñez-Hernández, Ingrid Balcells, Marta Muñoz, Anna Castelló, Gonzalo Vera, Lester J. Pérez, Raquel Egea, Gisela Mir, Sarai Córdoba, Rosa Rosell, Joaquim Segalés, Anna Tomàs, Armand Sánchez, José I. Núñez

**Affiliations:** 1 Departament de Ciència Animal i dels Aliments, Universitat Autònoma de Barcelona (UAB), Bellaterra, Barcelona, Spain; 2 Departament de Genètica Animal, Centre de Recerca en AgriGenòmica (CRAG), CSIC-IRTA-UAB-UB, Universitat Autònoma de Barcelona, Bellaterra, Barcelona, Spain; 3 Centre de Recerca en Sanitat Animal (CReSA), UAB-IRTA, Campus de la Universitat Autònoma de Barcelona, Bellaterra, Cerdanyola del Vallès, Spain; 4 Centro Nacional de Sanidad Agropecuaria (CENSA), La Habana, Cuba; 5 CRAG scientific services, Centre de Recerca en AgriGenòmica (CRAG), CSIC-IRTA-UAB-UB, Universitat Autònoma de Barcelona, Bellaterra, Barcelona, Spain; 6 Departament d’Agricultura, Ramaderia, Pesca, Alimentació i Medi Natural de la Generalitat de Catalunya (DAR), Barcelona, Spain; 7 Departament de Sanitat i Anatomia Animals, Universitat Autònoma de Barcelona, Bellaterra, Barcelona, Spain; 8 Program Infection and Immunity, FISIB, Bunyola, Spain; Sun Yat-sen University, China

## Abstract

Porcine production is a primary market in the world economy. Controlling swine diseases in the farm is essential in order to achieve the sector necessities. Aujeszky’s disease is a viral condition affecting pigs and is endemic in many countries of the world, causing important economic losses in the swine industry. microRNAs (miRNAs) are non-coding RNAs which modulates gene expression in animals, plants and viruses. With the aim of understanding miRNA roles during the Aujeszky’s disease virus [ADV] (also known as suid herpesvirus type 1 [SuHV-1]) infection, the expression profiles of host and viral miRNAs were determined through deep sequencing in SuHV-1 infected porcine cell line (PK-15) and in an animal experimental SuHV-1 infection with virulent (NIA-3) and attenuated (Begonia) strains. In the *in vivo* approach miR-206, miR-133a, miR-133b and miR-378 presented differential expression between virus strains infection. In the *in vitro* approach, most miRNAs were down-regulated in infected groups. miR-92a and miR-92b-3p were up-regulated in Begonia infected samples. Functional analysis of all this over expressed miRNAs during the infection revealed their association in pathways related to viral infection processes and immune response. Furthermore, 8 viral miRNAs were detected by stem loop RT-qPCR in both *in vitro* and *in vivo* approaches, presenting a gene regulatory network affecting 59 viral genes. Most described viral miRNAs were related to Large Latency Transcript (LLT) and to viral transcription activators *EP0* and *IE180*, and also to regulatory genes regarding their important roles in the host – pathogen interaction during viral infection.

## Introduction

Domestic pig (*Sus scrofa domesticus*) can be found worldwide because of their extraordinary importance in the production of red meat, lard and cured products [1]. Genetic improvement of the pig has evolved over the years, from selection for productive and reproductive characters to more recent interest in product quality and new non-economic factors, like animal health and welfare [2]. In this sense, safeguard farm sanitary conditions and animal maintenance are of great interest to provide a better quality of animal life and also a better pork quality and farm performance. Aujeszky’s disease (AD), also known as pseudorabies, is a disease of great economic importance, especially for intensive production systems that concentrate large farms located relatively near from each other. The etiological agent of the disease is the AD virus (ADV), also known as suid herpesvirus type 1 (SuHV-1) and pseudorabies virus (PRV), from the *Alphaherpesvirinae* subfamily, genus *Varicellovirus*. The disease causes significant economic losses in pig farms, mainly by reducing the litter size, abortions and slow growth of the animals, as well as restrictions on movements related to disease control. Young animals develop symptoms of central nervous infection while growing pigs and adults display respiratory signs. Pig is the primary host and the main disseminator of the disease. Moreover, in pigs surviving the acute infection, virus can develop a latency stage, and, subsequently, act as a reservoir [3]. It is a notifiable disease by the World Organisation for Animal Health (OIE).

SuHV-1 has a linear double-stranded DNA genome of about 142 Kb. Its genome has been fully sequenced [4] and comprises two unique sequences, the 5′ long and the 3′ short, the last one flanked by two inverted repeated sequences. The genome encodes more than 70 proteins [5], and two spliced transcripts are described: US1 and the Large Latency Transcript (LLT), while a third (UL15) has a putative splicing [4]. The virus has the possibility to induce latency in nervous system after an acute infection circularizing its genome and persisting like an episome with limited viral gene expression [6]. The molecular bases of latency in herpesviruses are still unknown. The control of the disease has been based on vaccination campaigns with the use of marker vaccines obtained by deletion of determined genes like TK and gE.

During last years, microRNAs (miRNAs) have been described and characterised as small non-coding RNAs involved in post transcriptional regulation of gene expression in animals, plants and some DNA viruses. They participate in a wide range of biological processes acting mainly through down-regulation of target messenger RNAs (mRNAs) by blocking protein translation or inducing mRNA degradation [7]–[13]. In addition, miRNA expression has also been associated with many different pathological processes, such as cancer, neurological disorders, inflammatory pathologies, cardiovascular diseases and infectious diseases [14]–[19].

Recently, several studies have confirmed the existence of viral miRNAs [20]–[22]. They are miRNAs codified by the viral genome, and mainly, they are described in the herpesvirus family [22]–[24]. These viral miRNAs work supporting the development of viral infection by using host miRNA biogenesis system to control the expression of their own and host genes [25], [26]. They can take advantage of a conserved gene regulatory mechanism within the host cell and establish a cellular environment conducive to viral replication [27]. Viral miRNAs act by regulating fundamental cellular processes in immunity, apoptosis and key steps in the transition from latent to lytic infection [6], [28]–[30]. In contrast to viral proteins, miRNAs can regulate host and own gene expression avoiding protein factors exposed to host antigenic immune response and, moreover, viruses also have relative little coding capacity [24], [27], [31]. Most studied herpesvirus express miRNAs during latency, even being a restrictive gene expression stage. In SuHV-1, for instance, the 13 miRNAs described up to date in miRBase database (v19, August 2012, URL: http://www.mirbase.org/, [32]–[34]) are encoded in the large latency transcript (LLT), the unique transcript expressed during the latency stage which generates the Latency Associated Transcripts (LAT) [35], [36]. However, little is known about the key roles that viral miRNAs can develop during the virus infection process. The virus is transmitted primarily through physical contact between pigs by secretions. First replication takes part in epithelial tissues and entries directly into the sensory nerve endings in the nasopharynx. The normal AD incubation period is from 2 to 6 days. There are several porcine cells used in many scientific studies that allow the laboratory culture of SuHV-1, such as the cell line derived from pig kidney PK-15 [3], [35] or dendritic cells [36], but there are no miRNAs studies involving pig tissues infected with SuHV-1.

In order to unravel the role of viral miRNAs during the ADV infection process, a SuHV-1 experimental infection using Landrace pigs was performed using two viral strains (NIA-3 and Begonia). Furthermore, *in vitro* infections with the same viral strains were also conducted. Characterisation and functional study of viral miRNAs are crucial to understand the molecular bases of herpesvirus pathogeny and, consequently, to develop mechanisms to fight against the disease and improve pork production.

## Materials and Methods

### Biological Material and Ethics Statement

Two strains of SuHV-1 were used in the experiment: the NIA-3 virulent strain and the Begonia attenuated strain. Begonia strain is derived from NIA-3 strain and is used as live attenuated vaccine.

A total of 20 4-week-old Landrace pigs were used in the experimental infection with both SuHV-1 strains. All animal procedures were performed in CReSA biosafety level 3 (BSL3) facilities (Centre de Recerca en Sanitat Animal, Universitat Autònoma de Barcelona, Bellaterra, Spain) and were carried out according to Spanish and European animal experimentation ethics law and approved by the institutional animal ethics committee of Universitat Auntònoma de Barcelona. Porcine Kidney (PK-15) cell lines were used for *in vitro* infection also with NIA-3 and Begonia SuHV-1 strains.

### Cell Culture and Animal Infections

PK-15 cell line was used for viral stocks preparation and *in vitro* infections. Cells were grown at 37°C and 5% CO_2_ and maintained in Dulbecco’s Modified Eagle Medium (DMEM) supplemented with 5% FCS, 100 ug/ml streptomycin and 100 IU/ml penicillin. A viral stock with a titre of 10^7,57^ TCID_50_/mL was prepared in PK-15 cells for NIA-3 virulent strain and 10^8,49^ TCID_50_/mL for Begonia attenuated strain.

PK-15 cell cultures at 65% of confluence were infected with a MOI of 0.05 with NIA-3 or Begonia strains. PK-15 cells inoculated with DMEM were maintained as non-infected cells. Samples were recovered at 12, 24 and 30 hours post infection (hpi). Cytopathic effect for both viruses was observed at 24 and 30 hours affecting 25 and 90% of the cells, respectively.

In the *in vivo* experimental infection, 9 animals were intranasally inoculated with 10^3^ TCID_50_ NIA-3 virulent strain, 6 with the same dose of Begonia attenuated strain and 5 animals with PBS as healthy pigs. At least one animal per group was euthanized at 4, 5 and 6 days post infection (dpi). The remaining animals were slaughtered at 7 dpi. Olfactory bulb (OB) and trigeminal ganglia (TG) samples for each animal were collected, immediately snap-frozen in liquid nitrogen and stored at −80°C until use. All samples were taken from CReSA BSL3 facilities (Bellaterra, Spain) under veterinary supervision.

### RNA Isolation

Total RNA was isolated using TRIzol® reagent (Invitrogen, Carlsbad, USA) following the manufacturer’s recommendations, quantified using ND 1000 Nanodrop® Spectrophotometer (Thermo Scientific, Wilmington, USA) and its quality was assessed on an Agilent 2100 Bioanalyzer using the RNA 6000 Nano kit (Agilent Technologies, Santa Clara, USA).

### Small RNA Library Construction and High Throughput Sequencing

A total of 21 libraries were performed in order to high throughput sequence them (Table 1). 9 libraries were from cell cultures infected with NIA-3, Begonia or mock-infected (control) cells at 12, 24 and 30 hpi. The remaining 12 libraries belonged to animal infections. BO and TG tissues were selected from 3 NIA-3 infected animals euthanized at 4, 6 and 7 dpi, 2 Begonia infected animals sacrificed at 4 and 7 dpi and 1 healthy animal necropsied at 5 dpi.

**Table 1:** Summary of employed samples in the study.

Sample	Infection	Group	Approach	PI^1^ sampletaken time	Sequenced by Ion Torrent	RT-qPCR detection
CC12	*In vitro*	Mock-infected	Cell culture	12 hours	Yes	Yes
CC24	*In vitro*	Mock-infected	Cell culture	24 hours	Yes	Yes
CC30	*In vitro*	Mock-infected	Cell culture	30 hours	Yes	Yes
NIA12	*In vitro*	NIA-3 infected	Cell culture	12 hours	Yes	Yes
NIA24	*In vitro*	NIA-3 infected	Cell culture	24 hours	Yes	Yes
NIA30	*In vitro*	NIA-3 infected	Cell culture	30 hours	Yes	Yes
BEG12	*In vitro*	Begonia infected	Cell culture	12 hours	Yes	Yes
BEG24	*In vitro*	Begonia infected	Cell culture	24 hours	Yes	Yes
BEG30	*In vitro*	Begonia infected	Cell culture	30 hours	Yes	Yes
32BO	*In vivo*	Healthy	OB	5 days	Yes	Yes
37BO	*In vivo*	NIA-3 infected	OB	4 days	Yes	Yes
41BO	*In vivo*	NIA-3 infected	OB	6 days	Yes	Yes
43BO	*In vivo*	Begonia infected	OB	7 days	Yes	Yes
45BO	*In vivo*	NIA-3 infected	OB	7 days	Yes	Yes
48BO	*In vivo*	Begonia infected	OB	4 days	Yes	Yes
32TG	*In vivo*	Healthy	TG	5 days	Yes	Yes
37TG	*In vivo*	NIA-3 infected	TG	4 days	Yes	No
41TG	*In vivo*	NIA-3 infected	TG	6 days	Yes	No
43TG	*In vivo*	Begonia infected	TG	7 days	Yes	Yes
45TG	*In vivo*	NIA-3 infected	TG	7 days	Yes	Yes
48TG	*In vivo*	Begonia infected	TG	4 days	Yes	Yes
31BO	*In vivo*	NIA-3 infected	OB	7 days	No	Yes
34BO	*In vivo*	NIA-3 infected	OB	4 days	No	Yes
35BO	*In vivo*	NIA-3 infected	OB	7 days	No	Yes
38BO	*In vivo*	NIA-3 infected	OB	5 days	No	Yes
39BO	*In vivo*	NIA-3 infected	OB	7 days	No	Yes
42BO	*In vivo*	Begonia infected	OB	6 days	No	Yes
44BO	*In vivo*	Begonia infected	OB	7 days	No	Yes
46BO	*In vivo*	Begonia infected	OB	7 days	No	Yes
47BO	*In vivo*	Begonia infected	OB	5 days	No	Yes
49BO	*In vivo*	Healthy	OB	7 days	No	Yes
50BO	*In vivo*	Healthy	OB	7 days	No	Yes
31TG	*In vivo*	NIA-3 infected	TG	7 days	No	Yes
34TG	*In vivo*	NIA-3 infected	TG	4 days	No	Yes
35TG	*In vivo*	NIA-3 infected	TG	7 days	No	Yes
36TG	*In vivo*	Healthy	TG	4 days	No	Yes
38TG	*In vivo*	NIA-3 infected	TG	5 days	No	Yes
39TG	*In vivo*	NIA-3 infected	TG	7 days	No	Yes
44TG	*In vivo*	Begonia infected	TG	7 days	No	Yes
46TG	*In vivo*	Begonia infected	TG	7 days	No	Yes
47TG	*In vivo*	Begonia infected	TG	5 days	No	Yes
49TG	*In vivo*	Healthy	TG	7 days	No	Yes

1 Post Infection. OB: Olfactory bulb, TG: Trigeminal ganglia.

Two last columns indicate which samples were used to create the libraries for sequencing by Ion PGM™ sequencer (n = 21) and which samples were added later in order to validate viRs through RT-qPCR (n = 40).

Small RNA fraction from each sample was excised and isolated from denaturing 12.5% polyacrilamide gel electrophoresis (PAGE) using miSpike™ (IDT®, Coralville, USA) as internal size marker. 50 µg of total RNA for each sample were loaded on separate gels to avoid cross-contamination. Gels were stained with GelStar® Acid Nucleic Gel Stain (Lonza, Basel, Switzerland) for UV visualization. Excised small RNA fraction were purified using Performa® DTR gel filtration cartridges (EdgeBio, Gaithersburg, USA). Briefly, 3′ and 5′ linkers from miRCat™ kit (IDT, Coralville, USA) were ligated at both ends of the small RNAs in two separated reactions using a T4 RNA ligase without ATP (Fermentas, Germany) and T4 RNA ligase with ATP (Ambion, Austin, USA), respectively. Between 3′ and 5′ primer ligations, the 60 nt RNAs were purified by PAGE to eliminate unligated products. Then, linked products were used to perform a reverse transcription reaction using the SuperScript™ III Reverse Transcriptase kit (Invitrogen™, Carlsbad, USA) and the cDNA obtained was amplified with the Expand High Fidelity System (Roche, Germany). PCRs were done to amplify the cDNA with primers complementary to 3′ and 5′ linkers and, in addition, they included multiplex identifiers at the 5′ end (a five nucleotide sequence tag) to allow differentiation between libraries. The number of PCR cycles was optimized for each sample, in order to minimize/avoid saturation, ranging from 21 to 30. Purification was carried out by using QIAquick PCR Purification Kit (Qiagen®, Germany). Libraries were quantified with Qubit™ fluorometer, Quant-IT™ (Invitrogen™, Carlsbad, USA), prepared to a 10^11^ DNA molecules/µL and equimolecular pooled according to their indexes. Ion Torrent adapters were ligated to 30 ng of pooled DNA and libraries were then amplified with Ion Torrent primers for 8 cycles, size selected (2% E-Gel Size Select, Invitrogen), and sequenced in four 314 chips in Ion PGM™ sequencer (Life Technologies, Carlsbad, USA) following the manufacturer’s protocol at DNA sequencing facilities at CRAG (Bellaterra, Spain). Software version for base calling was Torrent-Suite v2.0.1 (Life Technologies, Carlsbad, USA). Sequencing data was deposited at European Nucleotide Archive (ENA, http://www.ebi.ac.uk/ena/) with the accession number E-MTAB-1868.

### Sequence Processing Scheme

Primers sequences were trimmed and only those insert sequences between 15 and 29 nucleotides and with total number of sequences ≥3 were kept for further analysis. For porcine miRNA profiling, sequences were compared to all available miRNA sequences (miRBase v19) using local Blast. Parameters were set to 100% identity and up to 4 mismatches allowed at the end of the sequences to assume variability on 3′ and 5′ ends [37].

For viral miRNA discovery, sequences were blasted to SuHV-1 genome (NCBI Reference Sequence: NC_006151.1) considering 100% of alignment and identity (perfect match). Sequences positioned at annotated regions were discarded. Remaining sequences were clustered taking into account only the position in the genome. Hence, sequences positioned in the same region were grouped and the sequence with higher copy number (CN) was selected as the reference sequence for each cluster. A total of 14 clusters (viRs) were described and considered as putative viral miRNAs (Table S1). viRs were blasted to viral miRNAs described in miRBase v19 and compared to the described SuHV-1 miRNAs up to date [35], [36]. Flanking regions (50 nt) of the selected reference sequences for each cluster were used to predict pre-miRNA folding structure using MFold software [38] following the guidelines reported by Ambros et al. [39] for animal miRNAs (Figure S1). At the end, 8 viRs were selected for RT-qPCR detection (Table 2).

**Table 2:** Putative viral miRNAs (viRs) selected for RT-qPCR detection.

viR	information	Genome position^1^	Sequence (5′-3′)	Length	Copy number	Approach^2^	RT-qPCR detection
viR02	prv-miR-LLT1^a^	97929–97949	TCTCACCCCTGGGTCCGTCGC	21	2,299	CC+IT	+
viR04	prv-miR-7-5′^b^	99282–99301	CCGCCCCCGGGGGGTTGATG	20	27	CC	+
viR05	new viR	99302–99322	GGGATGGGCGCTCGGGGGTGA	21	7	CC	+
viR06	prv-miR-7-3′^b^	99342–99363	ACCACCGTCCCCCTGTCCCTCA	22	6	CC	+
viR08	new viR	99843–99862	TCAAACTTCCTCGTGTCCCC	20	57	CC	+
viR09	prv-miR-4^c^, moR8^b^	100203–100220	CGGAACCGGGTGCAGGCG	18	872	CC+IT	+
viR11	prv-miR-8-3′^b^	100267–100287	CAACCCTTCTGGAGCCCTACC	21	569	CC	+
viR14	new viR	102016–102040	TTCCGCCCGCTCTCCCACCGCCTTT	25	4	CC	+

a homology in miRBAse v19 (*p-value* <0.001).

b described at (Wu 2012).

c described at (Anselmo 2011).

1 Genome Position: start-end.

2 CC: Cell Culture (*In vitro* approach); IT: Infected Tissue (*In vivo* approach).

Differences in host and viral miRNA expression were assessed. Total number of sequences obtained for each porcine miRNA or viR was normalised by library size (in counts per thousand) and, then, averaged by group. Fold changes (FC) between groups were calculated using normalised data.

### RT-qPCR Detection

For RT-qPCR detection, additional samples from animal infected tissues were added (OB tissue: 5 NIA-3, 4 Begonia and 2 healthy; TG tissue: 5 NIA-3, 3 Begonia and 2 healthy, reaching a total of 40 samples. Two samples (37TG and 41TG from NIA-3 group) were not available for RT-qPCR detection due to lack of amount of cDNA. See Table 1). RT reactions were performed in duplicate using total RNA as previously described by Balcells et al. [40]. Briefly, 1 µg of total RNA in a final volume of 20 µL including 2 µL of 10x poly(A) polymerase buffer, 0.1 mM of ATP, 0.1 mM of each dNTP, 1 µM of RT-primer, 200 U of M-MuLV Reverse Transcriptase (New England Biolabs, USA) and 2,5 U of poly(A) polimerase (New England Biolabs, USA) was incubated at 42°C for 1 hour and at 95°C for 5 minutes for enzyme inactivation. Non template controls (NTC), minus RT and minus poly(A) polymerase controls for each sample were included.

DNA primers for each viR were designed following the methodology suggested by Balcells et al. [40] (Table S2). qPCR reactions were performed in duplicate in 20 µL final volume including 10 µL SYBR® Select Master Mix (Life Technologies, Carlsbad, USA), 300 nM of each primer and 5 µL of a 1∶20 dilution of the cDNA cell cultures or 1∶15 dilution of the cDNA animal infected tissues on an 7900HT Sequence Detection System (Applied Biosystems, Warrington, UK). Standard curves were generated by 5 fold serial dilutions of a pool of NIA-3 and Begonia infected cell cultures cDNAs in order to calculate the qPCR efficiency. Thermal profile was set as follows: 50°C for 2 min, 95°C for 10 min and 40 cycles at 95°C for 15 sec and 60°C for 60 sec. NTC and minus poly(A) polymerase controls were included. Melting curve analysis was included at the end of the qPCR to detect unspecific amplifications. Hsa-miR-93, Hsa-miR-25, Ssc-miR-106a, Ssc-miR-17-5p, Ssc-miR-26a were used as reference miRNAs [41], [42].

Quantities from each sample were obtained from the calibration (standard) curve added in each RT-qPCR reaction, and only those samples classified as quantifiable were used for statistical analyses. GeNorm v.3.5 software [43] was used to examine the stability of the reference miRNAs (M<1.5) and to obtain a normalization factor (NF). The quantity obtained from each miRNA was normalised by the NF and FCs were calculated in relation to the lowest normalised value. Finally, FCs were log_2_ transformed in order to perform the statistical analyses with the General Linear Models procedure of the Statistical Package for the Social Scientists (IBM® SPSS® Statistics 19; IBM Corporation, Armonk, USA). *In vitro* expression data were analysed to study the differences between the infection groups (NIA-3, Begonia and mock-infected) and time groups (12/24/30 hours) by a two-way analysis of variance (ANOVA). Significance threshold was set at α<0.001 due to the unequal sample size and variances. Estimated marginal means were also calculated using the least significance difference (LSD) as confidence interval adjustment. The same strategy was followed in NIA-3 *in vivo* expression data for the tissue group (OB, TG) and time groups (4/5/6/7 days).

### Target Prediction and Functional Analysis

DIANA - microT v3.0 web server [44], [45] was used to identify *in silico* potential mRNA targets for the most abundant and the differentially expressed porcine miRNAs. Porcine genes are not included in the current version of DIANA - microT v3.0 and predictions were based on the human mRNA:miRNA interactions assuming sequence conservation. *In silico* functional annotation of putative mRNA target genes for each miRNA were analyzed with WEB-based Gene Set Analysis Toolkit (WebGestalt, [46]). Predicted miRNA targets were functionally annotated through the biological process information supported by Gene Ontology (GO, [47]) and the pathways in which they were involved were described by using the Kyoto Encyclopedia of Genes and Genomes database (KEGG, [48], [49]). Over or under represented functional categories were identified with hypergeometric test corrected by the multiple test adjustment proposed by Benjamini & Hochberg [50]. Significant threshold was set at α<0.05.

The miRanda algorithm [51] was utilised to predict putative targets for viral miRNAs (viRs), using the following parameters: -sc 140 -en 20. Strict alignments were required in the seed region (G:U wobble is not allowed). Cytoscape 2.8.2 software [52], [53] was used to build the gene regulatory network formed by viral miRNAs and their target genes from SuHV-1 genome. Most abundant DE porcine miRNAs in infected samples were also added to gene regulatory network.

## Results

### Clinical Signs of Infected Animals

Seven animals inoculated with NIA-3 strain developed clinical signs, affecting nervous system, and only two animals euthanized at 4 dpi only presented pyrexia, maybe due to the early time that were euthanized. Any animal inoculated with Begonia strain presented clinical signs.

### Sequence miRNA Annotation

A total of 21 small RNA libraries (9 from PK-15 cell line cultures and 12 from *in vivo* animal infection) were sequenced in a Ion PGM™ sequencer (Life Technologies). After trimming the adaptors sequences, inserts ranging from 15 to 29 nt (corresponding to miRNA size) and found more than two times (copy number (CN)>2) were aligned to miRBase database (v19). 435,434 counts (4,029 unique sequences) could be aligned to miRBase database, representing a 37% of total counts used in this study (Table 3). In PK-15 cell line libraries, 229 miRNAs were described, 109 of them had been already described in pig, 113 were orthologous miRNAs and 7 were viral miRNAs (6 from SuHV-1 and 1 from Rhesus Rhadinovirus – RRV). In *in vivo* animal infection libraries, a total of 302 miRNAs were described, 150 that had been previously described in *Sus scrofa*, 151 were orthologous and only 1 viral miRNA from SuHV-1 was found. No viral miRNAs were identified in mock-infected PK-15 cell cultures and in healthy animals.

**Table 3:** Summary of sequence processing scheme.

	PK-15 cell line cultures	Animal infection	General
Raw reads obtained	705,846	855,701	1,561,547
Trimmed and non empty reads	500,870	694,944	1,195,814
Counts ranging from 15 to 29 nt	490,848	674,538	1,165,386
Counts aligned to miRBase (unique sequences)	212,519 (2,151)	222,915 (2,841)	435,434 (4,029)
miRNA profile	229	302	361
*Sus scrofa* miRNAs	109	150	193
Orthologous miRNAs	113	151	161
Viral miRNAs	7	1	7
Counts aligned to SuHV-1 genome (unique sequences)	3,948 (47)	31 (5)	3,979 (50)
Putative viral miRNAs (viRs)	14	2	14

### Differential Expression Analysis

#### *In vitro* infection

Looking for miRNA abundance differences among groups (NIA-3 infected, Begonia infected and mock-infected), 138 miRNAs (60%) were more expressed in mock-infected group and 91 miRNAs (40%) were more expressed in infected groups. Interestingly, 35 miRNAs were specifically expressed in infected groups, whereas two miRNAs were only expressed in mock-infected group.

miRNAs were considered differentially expressed (DE) when fold change (FC) difference between groups was greater than 5 or when a miRNA was not expressed in both infected groups or in mock-infected group. Out of the 229 miRNAs described in the *in vitro* profile, 111 (48%) miRNAs were DE; of which, 69 (30%) were up-regulated in mock-infected group and 42 (18%) over-expressed in both infected groups (Table S3). Looking at most abundant miRNAs (CN>100), we observed a clear predominance of those miRNAs over-expressed in mock-infected group (Table 4), such as miR-125b-5p, miR-99b-5p and miR-100. The only miRNA over-expressed in infected groups with CN>100 was the viral miR-LLT1 (CN = 3,280). On the other hand, comparing both infected groups, two miRNAs were up-regulated in Begonia infected group and down-regulated in NIA-3 infected group, being, consequently, DE between both infected groups: miR-92a and miR-92b-3p. Interestingly, miR-2887 presented the highest FC differences between mock-infected and NIA-3 infected groups (204 fold). Although it was over-expressed in mock-infected group, it also was DE between NIA-3 and Begonia infected groups, like miR-4286 and let-7d-3p.

**Table 4:** The most abundant (CN>100) DE miRNAs between infected groups (NIA and BEG) and mock-infected group (MI) in the *in vitro* approach.

miRNA	Counts	BEG *vs*. MI	NIA *vs*. MI	NIA *vs*. BEG
miR-92a	5,495	3.37	−1.54	−5.20
miR-125b-5p	4,896	−7.37	−8.36	−1.13
miR-LLT1	3,280	–	–	−4.04
miR-99b-5p	2,220	−3.48	−6.90	−1.99
miR-100	1,891	−5.47	−7.90	−1.44
miR-92b-3p	704	2.18	−2.62	−5.70
miR-2779	512	−1.38	−5.41	−3.91
miR-2887	479	−20.24	−203.79	−10.07
miR-2904	408	−13.18	−21.41	−1.62
miR-125a-5p	346	−8.13	−13.31	−1.64
miR-5109	294	−9.38	−18.18	−1.94
miR-4286	243	−2.60	−16.78	−6.45
miR-30a-5p	226	−7.87	−9.07	−1.15
let-7b-5p	209	−3.64	−5.76	−1.58
miR-26a-5p	206	−5.33	−3.84	1.39
miR-339-5p	176	−4.97	−22.89	−4.60
let-7d-3p	171	−1.36	−8.66	−6.38
miR-19b	144	−6.97	−4.30	1.62
miR-23a-5p	126	−11.83	−27.63	−2.34
let-7i-5p	122	−5.40	−6.29	−1.17
miR-505-5p	117	−15.46	−25.93	−1.68
miR-4454	104	−3.32	−7.51	−2.26

Focusing on the described viral miRNAs, miR-LLT9 (CN = 3) was only described in NIA-3 infected group. miR-LLT6, miR-LLT8 and miR-LLT11a were more expressed in NIA-3 infected group and miR-LLT1 and miR-LLT2 presented more expression in Begonia infected group. Only miR-LLT2 was DE between infected groups, although its abundance was only 13 counts. The viral miRNA from RRV (miR-rR1–5, CN = 415) was more expressed in mock-infected group, therefore it was not considered a real SuHV-1 encoded miRNA, but a miRNA expressed in PK-15 cells.

#### *In vivo* infection

In contrast to *in vitro* approach, miRNA abundance differences revealed a turnaround in which 211 (70%) miRNAs were more expressed in infected NIA-3 and Begonia groups, and the remaining 91 (30%) miRNAs were more expressed in healthy group. Moreover, 75 miRNAs were specifically expressed in infected groups, and only two miRNAs were only expressed in healthy group.

Focusing on those more expressed miRNAs in infected groups and DE between them, we observed that miR-206 (FC = 648), miR-133a (FC = 108), miR-133b (FC = 88) and miR-378 (FC = 5) were more expressed in NIA-3 group (Table S4). On the other hand, miR-137 (FC = −6) and miR-1249 (FC = −5) were the most expressed miRNAs presenting an up-regulation in Begonia infected group. The only viral miRNA detected (miR-LLT1, CN = 9) were only expressed in NIA-3 infected group, diverging from its expression in the *in vitro* approach, where it was more expressed in Begonia infected group.

Taking into account both approaches, out of 361 total described miRNAs, 170 miRNAs were present in both *in vitro* and *in vivo* profiles and 191 miRNAs were present only in one profile (59 miRNAs from *in vitro* approach and 122 miRNAs from *in vivo* approach, see Table S5). There were notable differences in the expression of profile-shared miRNAs. Only 27 miRNAs followed the same expression profile in both approaches, and 11 of them where DE in both groups: 4 miRNAs (miR-26b-5p, miR-29b-2-5p, miR-450b-5p and miR-450c-5p) were only expressed in infected groups and the remaining 7 miRNAs (let-7b-3p, miR-193b, miR-345-5p, miR-1306-5p, miR-2779, miR-2898 and miR-4286) were more expressed in mock-infected or healthy group, although all of them were expressed at low levels (CN<100) in both profiles. On the other hand, 35 miRNAs (CN>100) showed a different expression pattern between *in vitro* and *in vivo* approaches, but only two miRNAs, let-7i-5p and miR-30d-5p, presented differential expression regarding both approaches. In both cases, they were up-regulated in mock-infected group in the *in vitro* approach, and their expression changed in the *in vivo* approach, having a major expression in NIA-3 infected group. Among the non-profile-shared miRNAs, there were 6 high expressed miRNAs (CN>1,000 counts): miR-99a-5p (11,178 counts), miR-10a-5p (3,570 counts), miR-133a (1,990 counts), miR-218b (1,887 counts), miR-9-3p (1,620 counts) and miR-129a (1,566 counts). All these 6 miRNAs were expressed in the *in vivo* profile, except miR-10a-5p which was expressed in the *in vitro* profile.

### Target Prediction and Functional Analysis of Host miRNAs

*In silico* target prediction were performed for those most abundant and DE porcine miRNAs presenting differential expression between NIA-3 and Begonia infected groups: miR-92a and miR-92b-3p from the *in vitro* approach and miR-206, miR-133a, miR-133b and miR-378 from the *in vivo* approach. A total of 1,629 target genes were identified (Table S6) and functionally analysed through KEGG pathways database. Significant related pathways to target genes were found for all miRNAs except for miR-378. Interestingly, many pathways related to viral infection process and immune response resulted significant, such as RIG-I-like receptor signalling pathway, B and T cell receptor signalling pathways, Fc gamma R-mediated phagocytosis and chemokine signalling pathway. Furthermore, miRNAs targets were also involved in more general biological processes, such as cell cycle, apoptosis, endocytosis, focal adhesion and RNA transport and degradation. Finally, pathways focused on nervous system were also described, like axon guidance, neurotrophin signalling pathway and long-term potentiation.

### Viral miRNA Identification and RT-qPCR Detection

Trimmed and non-empty reads ranging from 15 to 29 nt were also aligned to SuHV-1 genome (NC_006151.1) considering only 100% of alignment and identity. A total of 3,979 counts (50 unique sequences) yielded a positive match, 3,948 counts (47 unique sequences) from PK-15 cell line libraries and 31 counts (5 unique sequences) from *in vivo* animal infection libraries (Table 3). All sequences homologous to an annotated region were removed and the remaining sequences were clustered by the position in the SuHV-1 genome resulting in 14 putative viral miRNAs. All of them were identified in cell cultures and only 2 of them could also be identified in animal infection samples: viR02 (mir-LLT1 in miRBase v19) and viR09 (Table 2). None of them were found in mock-infected cell cultures or in healthy animals. From these 14 putative viral miRNAs, viR03, viR07, viR10, viR12 and viR13 were already described at miRBase as miR-LLT2, miR-LLT6, miR-LLT8, miR-LLT9 and miR-LLT11a, respectively, and, consequently, were discarded for RT-qPCR detection. viR02 was also described at miRBase but it was maintained to be the most expressed viral miRNA in the study. viR01, the only putative viR which was not located at LLT intronic region, did not succeed at performing their pre-miRNA structure, and was also removed. Thereafter, 8 viRs remained to confirm their expression (Table 2) and a detection protocol through RT-qPCR was designed for them, which were successfully amplified with high RT-qPCR efficiencies, ranging from 90% to 110%, and standard curves correlations were at least of 0.98. Ct and Tm values, as well as amplification and melting curves can be found at supporting information (Datasets S1–S4).

Out of these 8 identified viRs, 5 of them were already described in previous studies [35], [36] and remaining 3 viRs were new described putative viRs (Table 2). viR09 was described as miRNA offset RNA (moRNA) in the Wu study [35] and our study also confirmed that viR09 was the moRNA originated from miR-LLT8 (Figure S1). As moRNAs functions remain unknown [54], [55], the described moRNA in this study (viR09) was kept in the functional analysis, as it was found in a major relative abundance than its contiguous mature miRNA and it was favourably detected through RT-qPCR.

**Figure 1:**
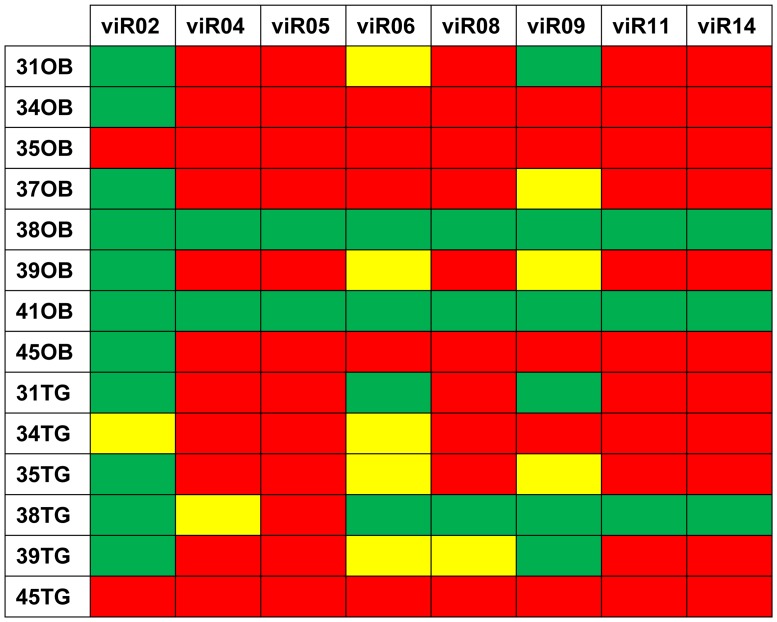
Results in viR detection through RT-qPCR for NIA-3 group from *in vivo* animal infection. Each line represents one sample and each column describes each studied viR. OB: Olfactory Bulb; TG: Trigeminal Ganglia. Colour determines either the viR could be detected or not in the sample. Green reflects the detection of the viR in the sample in quantifiable parameters. Yellow defines the detection of the viR without quantifiable parameters. Red determines that the viR was not detected in the sample. Parameters were considered quantifiable when: (1) viR detection is achieved in all reactions done per sample (4 RT-qPCR reactions, corresponding to 2 different RT per duplicate) and (2) a minimum of 3 out of the 4 RT-qPCR reactions have a cycle threshold (Ct) under 35.

All detected viRs were located in the intronic region of the LLT transcript, such as all previously described viRs. They were detected in the NIA-3 and Begonia groups from cell cultures and only presented expression in some samples from NIA-3 group from *in vivo* infection (Figure 1). Begonia group from *in vivo* infection as well as all mock-infected and healthy animal samples from *in vitro* and *in vivo* infection resulted without expression for all studied viRs.

Statistical analyses from RT-qPCR *in vitro* data revealed no significant expression differences between NIA-3 and Begonia groups. Referring at time group, there were significant expression differences (*p-value* <.001), being viRs more expressed in those samples taken at 24 or 30 hpi than those taken at 12 hpi (*p-value* <.001), excepting for viR05, which showed more expression in 12 hpi than in 30 hpi, although without significant differences in any time group.

Regarding NIA-3 *in vivo* group RT-qPCR data, statistical analyses only could be performed for viR02, viR06 and viR09. Results showed differential expression (*p-value* <.001) for tissue group and time group in all three viRs, being more expressed in olfactory bulb than in trigeminal ganglia. In time group, viRs presented differential expression in 4, 5, 6 and 7 dpi, presenting directional increasing from dpi 4 to dpi 6 and an interesting decrease of their expression at dpi 7.

### Gene Regulatory Network Viral miRNAs

**Figure 2:**
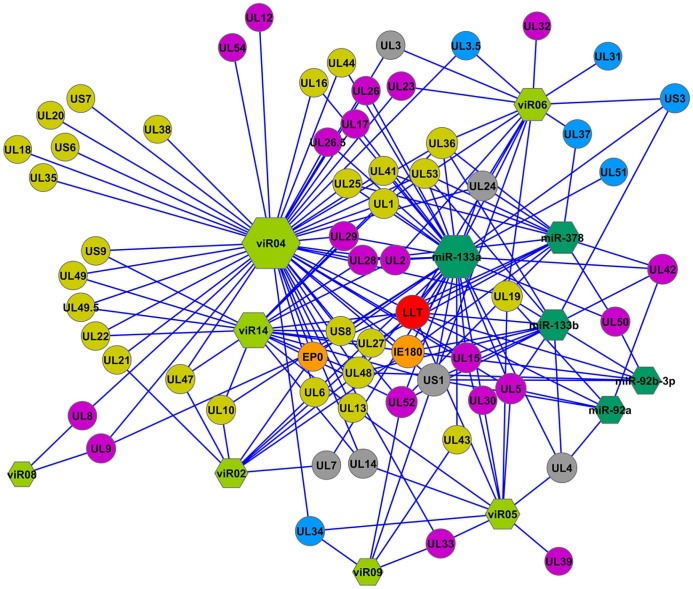
Regulatory gene network between SuHV-1 genes and detected viRs and most abundant DE host miRNAs in infected samples. Node shape represents either is a viral miRNA (hexagon, bright green), a host miRNA (hexagon, dark green) or a viral gene (circular). Node size simulates the number of interactions, which is directly proportional. Viral gene function is represented by colour: yellow means structural function (virion envelope, tegument or capside proteins); purple means regulatory function; blue represents viral egress function and grey means unknown function. LLT transcript is marked in RED and *EP0* and *IE180* viral activators are marked in orange.

miRanda algorithm was used to form the gene interaction network between the 8 described viRs and the 70 annotated genes in SuHV-1 genome, including large latency transcript (LLT). It found 110 significant interactions between 7 viRs and 59 SuHV-1 genes (Figure 2). No interactions could be retrieved for viR11. The most interacting viRs were viR04, viR14 and viR06 reaching at 48, 20 and 15 gene interactions, respectively. LLT was the gene associated to more viRs, up to 5: viR02, viR04, viR05, viR06 and viR14.

To test if porcine miRNAs can regulate viral genes, most abundant DE porcine miRNAs between viral strains were also added to the gene interaction network. Thus, miR-92a, miR-92b-3p, miR-133a, miR-133b, miR-378 and miR-206 generated a total of 71 significant interactions with 37 SuHV-1 genes (Figure 2). miR-133a had 33 gene interactions, including LLT and the regulatory genes *EP0*, *IE180*, *UL41* and *UL48.* miR-92a, miR-92b-3p, miR-133b, and miR-378 also interacted with LLT and with the regulatory genes IE180, UL41 and UL48. miR-206 was not associated to any viral gene.

## Discussion

This study is the first work that describes the host and pathogen miRNA expression profile in an *in vitro* and as well as *in vivo* SuHV-1 infection through high throughput sequencing. Regarding the host miRNAome, 193 porcine miRNAs out of a total of 306 annotated miRNAs in miRBase v.19 were described. Approximately half of all described miRNAs were orthologous, evidencing that there are still many porcine miRNAs to be described in order to complete the current annotation of porcine miRNAs in miRBase.

First conclusion comparing both profiles was that there were notable differences among described miRNAs and also among their expression pattern. Looking at those most abundant miRNAs in each profile (CN>100), only 33% of miRNAs were shared. This can be expected because our study has been developed with material of different nature: cell lines derived from kidney and nervous tissues from an acute infection, helping to perform different miRNA profiles and, assuming different expression patterns. Just comparing the PK-15 profile with the kidney profile from Timoneda et al., previous study [56], the shared miRNAs were up to 42%, more than in the *in vivo* profile, showing some analogy between PK-15 cells and kidney. However, PK-15 cell lines were chosen because they were a good substrate for the laboratory culture of SuHV-1, and, on the other hand, OB and TG tissues were chosen to be the tissues where the virus replicates at high level and, therefore, they are the election tissues for viral detection.

Sequenced libraries revealed a different porcine miRNA expression profile when the SuHV-1 infection was present, and presented some homology between the two viral strains used in this study, NIA-3 (virulent strain) and Begonia (attenuated strain). The change of expression of some porcine miRNAs between infected and mock-infected or healthy samples reflects that miRNAs can play key roles during the viral infection process, where virus can affect cellular miRNA expression profile on their own benefit. In this sense, many porcine miRNAs were described to be down-regulated in the infected samples, particularly in the *in vitro* infection, such as miR-125b-5p, miR-99b-5p, miR-100 and miR-2887, suggesting that viral mechanisms can affect host miRNA expression. For instance, miR-100 has already been described to be down-regulated in human cytomegalovirus infection [57], showing that it could be associated to viral infection.

Focusing on the *in vivo* approach, there were miRNAs DE between virulent and attenuated strains, particularly more expressed in NIA-3 infected group, like miR-133a (FC = 108), miR-133b (FC = 88), miR-378 (FC = 5) and miR-206 (FC = 648), suggesting that they could work activating those pathways related to the response against the viral infection. Interestingly, it seems that they could regulate targets that would be involved to immune response, such as RIG-I-like receptor signalling pathway, responsible for detecting viral pathogens, or also B and T cell receptor signalling pathways, which are key components for the activation of adaptive immunity and T lymphocytes, respectively, and ensure an efficient response of the immune system. They also were related to Fc gamma R-mediated phagocytosis, which plays an important role in host-defence mechanisms through the uptake and destruction of infectious pathogens, and chemokine signalling pathway, which works on the inflammatory immune response. miR-206 was previously described to be up-regulated in influenza A virus experimental infected pigs, and it was reported to interact with the antimicrobial protein mucin 1 (MUC1), MyD88 involved in secretion of type I IFN and pro-inflammatory cytokines, and chemokine CCL2 [58]. miR-133b plays a role in the maturation on midbrain dopaminergic neurons [59] and could be involved in the development of nervous system signs showed in NIA infected animals.

This change in miRNA expression could be explained by the differences in both virus strains. Begonia is an attenuated strain which has been genetically modified, removing glycoprotein E gene (gE) and thymidine kinase gene (tK), in order to be less effective at virus replication. In this sense, these miRNAs could increase their expression when the virulent strain NIA-3 is present in order to react against infection. It must be taken in consideration that further investigations to elucidate the biological roles of these miRNAs are clearly needed, including RT-qPCR validations, as these results are supported only by high throughput sequencing data.

Another miRNA which changed its expression pattern between NIA-3 and Begonia infections was miR-92a. While in the *in vitro* samples miR-92a was DE presenting an up-regulation in Begonia strain infection, in the *in vivo* samples its expression was higher in NIA-3 strain infection. A wide range of significant pathways were associated to miR-92a putative targets, from pathways related to neuronal functions such as axon guidance, neuro active-ligand receptor activation and neurotrophin signalling pathway, to pathways related to more general cellular functions like endocytosis, RNA degradation and focal adhesion. We could argue that in the *in vitro* approach, the Begonia strain virus does not receive a strong defensive response from the host and it could replicate in a similar level as NIA-3 begonia strain, according to RT-qPCR results from viral miRNAs expression. In this sense, the host response generated in the Begonia *in vivo* infection could cause the miR-92a fall of expression. Regarding to miR-92b-3p, it maintained its expression in both approaches, being more expressed in Begonia strain infection than in NIA-3 strain infection. As miR-92a, miR-92b-3p target genes were associated to a wide range of biological processes as well as nervous system pathways. As its expression has shown invariable in different tissues, despite of the viral infection, it could play a more general cell function.

We observed that the development of the viral infection did not happen in the same way in cell cultures than in tissues from experimentally infected pigs. The ability to react and fight against a viral attack was not the same in *in vitro* and *in vivo* due, among others, to the lack of immune response in cell culture. The miRNA expression variability can be determined by the frequency of the most expressed miRNAs in each approach. In the *in vitro* infection, miR-23a-3p was the most expressed miRNA representing the 50% of annotated reads, while miR-125b-5p was the most expressed miRNA in the *in vivo* infection, in both olfactory bulb and trigeminal ganglia, and only represented the 25% of the annotated reads. Thus, there was a major variability in the animal infection model. In comparison to previous studies, Anselmo et al. [36] described miR-21 as the most expressed miRNA in their study by using dendritic cells as approach, representing almost 91% of all small RNA sequence tags, while Wu et al. [35] found miR-7f as the most expressed miRNA in a PK-15 cell line culture, being the 17% of total small RNA reads. As miRNAs expression are spatial and temporal specific, the disparity of results must be taken in consideration.

Moreover, miRNAs expression differences in *in vitro* and *in vivo* profiles became more evident by using two different strains of SuHV-1. In this sense, the two strains were capable of infecting cell cultures producing cytopathic effect, while in the animals, the attenuated strain was unable to produce clinical signs showing a less effective replication, and therefore, we were not able to detect viral miRNAs in these samples through RT-qPCR. Focusing on viral miRNAs (viRs), we could detect differences in sequenced data expression between *in vitro* and *in vivo* groups through miRBase homology. In cell cultures, 7 viRs could be detected, being miR-LLT1 (CN = 3,280) the most expressed viral miRNA. In the animal infection, however, only one viR could be detected in sequenced data, which was also miR-LLT1 (CN = 9). Even so, viral miRNA identification based on genome sequence homology approach was able to detect and confirm through RT-qPCR the expression of 8 putative viRs. It is of great interest because this study confirms the expression of viral miRNAs originated from SuHV-1 LLT transcript during lytic infection, assuming their role in the early stage infection process. Nevertheless, due to the difficulties to obtain enough small RNAs especially from the OB and TG tissues for northern-blot analysis, we describe the eight novel miRNAs as “putatives”.

RT-qPCR expression data showed that there were no significant differences in viral miRNAs expression between infected groups (NIA-3 virulent strain and Begonia attenuated strain) in the *in vitro* infection, and confirmed that no expression were detected in Begonia infected group in the *in vivo* infection. As expected, samples taken at 24 and 30 hpi in the *in vitro* infection presented more viR expression than samples taken at 12 hpi. In the *in vivo* approach, significant differential expression were described between all 4 times where samples were taken (4, 5, 6 and 7 dpi), and presented an increasing expression from day 4 to day 6 and revealing a sudden decrease at day 7. This result could be explained because the expressed miRNAs in this initial phase of the acute infection in our animal experiment could play a role in the establishment of the infection and can be different from miRNAs expressed in a latent phase, which can be the same than whose are found in cell culture, as has been found for other herpesvirus [60].

Viral gene network analysis deciphered the complex interaction between the described putative viRs and SuHV-1 genes (Figure 2). Interestingly, putative viRs were associated with almost all described SuHV-1 genes [5], as described in a previous study [35]. Large Latency Transcript (LLT) was the most interacted transcript by viRs, confirming the important role of them in the latency stage development. As all previously described viral miRNAs in SuHV-1, the new putative viRs described in this study were also encoded in the LLT intronic region, confirming it as the primary and the unique far miRNA precursor region. In addition, most DE host miRNAs were also associated to viral regulatory mechanisms, relating the interaction of host miRNAs with the expression of viral genes. The gene regulatory network deciphered the important role of viR04 presenting up to 24 gene interactions related to structural role, from proteins related to the virion envelope (glycoproteins) to tegument and capside proteins. Thus, viR04 may play an important structural role, although it also presented numerous interactions to genes involved in processes like DNA repair and recombination, DNA cleavage, encapsidation and packaging. viR02 and viR14 were mainly associated to structural genes related to virion envelope glycoproteins and tegument proteins, and could play important roles during viral entry and virion morphogenesis. In contrast, viR05 was associated to non-structural genes related to DNA cleavage and encapsidation and DNA replication and packaging. Curiously, viR06 presented many interactions with genes related to viral egress (*UL3.5*, *UL31*, *UL37* and *UL53*), which could play an interesting role in the viral egress process. viR02, viR04, viR06 and viR14 were also linked to genes associated to regulatory functions (*EP0*, *IE180*, *UL41*, *UL48* and *UL54*). viR09, the moRNA described in this study, was associated to few viral genes, which were associated to DNA cleavage, packaging and replication functions, as well as viral egress and structural role regarding virion envelope proteins. Regarding the involvement of host miRNAs in the regulatory network, miR-133a, miR-133b, miR-92a, miR-92b-3p and miR-378 were related to LLT and the regulatory genes *EP0*, *IE180*, *UL41* and *UL48*. It could mean that they could play an active role in fighting against the viral infection process. Moreover, miR-133a was related with many structural and non structural genes, confirming the importance of miR-133a in the ADV infection, as it was previously determined to be related with host-defence pathways.

The potential regulation roles developed by miRNAs not only in the own host gene machinery but also in the viral infection mechanisms were described in this study, using *in vitro* and *in vivo* approaches. In addition, this study increases the knowledge about miRNAs putative functional roles in a herpesvirus infection and their host-pathogen interactions, supported by an *in vivo* approaching using nervous tissue.

## Supporting Information

Figure S1 **Predicted viral miRNA folding using MFold software^1^.** **^1^:** [40]. Green line points out the miRNA position into the pre-miRNA structure.
(TIFF)

Table S1 **Described 14 viral clusters from high throughput sequencing.** **^1^:** Genome Position: start-end. **^2^:** Approaches where viRs were found. CC: Cell Culture (*In vitro* approach); IT: Infected Tissue (*In vivo* approach).
(DOCX)

Table S2 **Primers and viR/miRNA sequences used for the RT-qPCR design.**
(DOCX)

Table S3 **Complete** ***in vitro*** **miRNA profile from sequencing and differential expression analysis through fold change between NIA-3 strain infected group (NIA), Begonia strain infected group (BEG) and mock-infected group (MI).**
(DOCX)

Table S4 **Complete** ***in vivo*** **miRNA profile from sequencing data and differential expression analysis through fold change between NIA-3 strain infected group (NIA), Begonia strain infected group (BEG) and healthy group (HE).**
(DOCX)

Table S5 **Described miRNAs in only one approach: cell culture (CC) or infected tissue (IT).**
(DOCX)

Table S6 ***In silico*** **target genes predicted for the most abundant DE porcine miRNAs in the infected groups.**
(DOCX)

Dataset S1 **Ct and Tm values of qPCR** ***in vitro*** **samples.**
(XLSX)

Dataset S2 **Ct and Tm values of qPCR** ***in vivo*** **samples.**
(XLSX)

Dataset S3 **Amplification and melting curves of qPCR** ***in vitro*** **samples.**
(PDF)

Dataset S4 **Amplification and melting curves of qPCR** ***in vivo*** **samples.**
(PDF)
