# Supplementary figures and images for: The Role of Viral and Host MicroRNAs in the Aujeszky’s Disease Virus during the Infection Process
Source: PLoS One. 2014 Jan 24;9(1):e86965. doi: 10.1371/journal.pone.0086965 (PMC3901728; doi:10.1371/journal.pone.0086965)

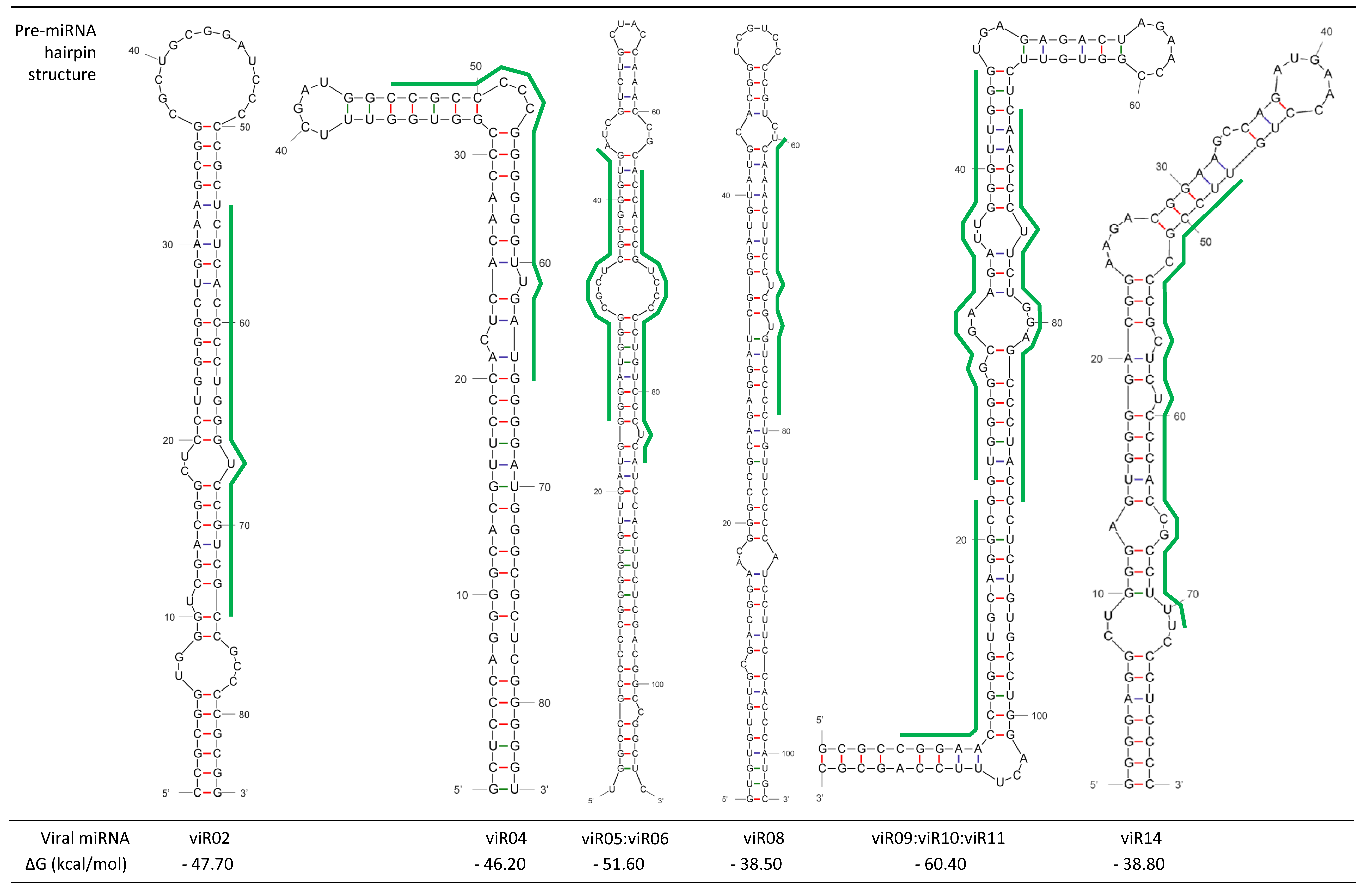

Supplement: Figure S1 — Predicted viral miRNA folding using MFold software1. 1: [40]. Green line points out the miRNA position into the pre-miRNA structure. (TIFF) [file pone.0086965.s001.tiff]
