# Supplementary material for: The Role of Viral and Host MicroRNAs in the Aujeszky’s Disease Virus during the Infection Process
Source: PLoS One. 2014 Jan 24;9(1):e86965. doi: 10.1371/journal.pone.0086965 (PMC3901728; doi:10.1371/journal.pone.0086965)
Supplement: Table S1 — Described 14 viral clusters from high throughput sequencing. 1: Genome Position: start-end. 2: Approaches where viRs were found. CC: Cell Culture (In vitro approach); IT: Infected Tissue (In vivo approach). (DOCX) [file pone.0086965.s002.docx]

**Table S1. Described 14 viral clusters from high throughput sequencing.**

| **viR** | **Sequence (5'-3')** | **Length** | **Genome position^1^** | **IsomiRs** | **Copy number** | **Approach^2^** |
| --- | --- | --- | --- | --- | --- | --- |
| viR01 | ACAACCCGGAGCGCCGCCGTC | 21 | 63745-63765 | 1 | 4 | CC |
| viR02 | TCTCACCCCTGGGTCCGTCGC | 21 | 97929-97949 | 5 | 2,299 | CC+IT |
| viR03 | CTCATCCCGTCAGACCTGCGCC | 22 | 98386-98407 | 2 | 12 | CC |
| viR04 | CCGCCCCCGGGGGGTTGATG | 20 | 99282-99301 | 2 | 27 | CC |
| viR05 | GGGATGGGCGCTCGGGGGTGA | 21 | 99302-99322 | 1 | 7 | CC |
| viR06 | ACCACCGTCCCCCTGTCCCTCA | 22 | 99342-99363 | 1 | 6 | CC |
| viR07 | CGTACCGACCCGCCTACCAGGC | 22 | 99550-99571 | 2 | 13 | CC |
| viR08 | TCAAACTTCCTCGTGTCCCC | 20 | 99843-99862 | 5 | 57 | CC |
| viR09 | CGGAACCGGGTGCAGGCG | 18 | 100203-100220 | 5 | 872 | CC+IT |
| viR10 | GTGGGGGCGAAGATTGGGTTGGG | 23 | 100221-100243 | 1 | 10 | CC |
| viR11 | CAACCCTTCTGGAGCCCTACC | 21 | 100267-100287 | 6 | 569 | CC |
| viR12 | CGAGGAGATGTGGAGGGGTG | 20 | 100377-100396 | 1 | 3 | CC |
| viR13 | AGGCTGGGAGTGGGGACGGAAGA | 23 | 101974-101996 | 2 | 38 | CC |
| viR14 | TTCCGCCCGCTCTCCCACCGCCTTT | 25 | 102016-102040 | 1 | 4 | CC |

**^1^:** Genome Position: start-end.

**^2^:** Approaches where viRs were found. CC: Cell Culture (*In vitro* infection); IT: Infected Tissue (*In vivo* infection).
