# Supplementary material for: The Role of Viral and Host MicroRNAs in the Aujeszky’s Disease Virus during the Infection Process
Source: PLoS One. 2014 Jan 24;9(1):e86965. doi: 10.1371/journal.pone.0086965 (PMC3901728; doi:10.1371/journal.pone.0086965)
Supplement: Table S2 — Primers and viR/miRNA sequences used for the RT-qPCR design. (DOCX) [file pone.0086965.s003.docx]

**Table S2. Primers and viR/miRNA sequences used for the RT-qPCR design.**

| **viR/miRNA** | **Sequence (5′-3′)** | **Forward primer (5′-3′)** | **Reverse primer (5′-3′)** |
| --- | --- | --- | --- |
| viR02 | TCTCACCCCTGGGTCCGTCGC | AGTCTCACCCCTGGGTC | TCCAGTTTTTTTTTTTTTTTGCGAC |
| viR04 | CCGCCCCCGGGGGGTTGATG | CGCCCCCGGGGGGTT | AGGTCCAGTTTTTTTTTTTTTTTCATC |
| viR05 | GGGATGGGCGCTCGGGGGTGA | AGGGGATGGGCGCTCG | TCCAGTTTTTTTTTTTTTTTCACCC |
| viR06 | ACCACCGTCCCCCTGTCCCTCA | CACCACCGTCCCCCTG | TCCAGTTTTTTTTTTTTTTTGAGGGA |
| viR08 | TCAAACTTCCTCGTGTCCCC | GCAGTCAAACTTCCTCGTG | TCCAGTTTTTTTTTTTTTTTGGGGA |
| viR09 | CGGAACCGGGTGCAGGCG | GCAGCGGAACCGGGTG | CCAGTTTTTTTTTTTTTTTCGCCTG |
| viR11 | CAACCCTTCTGGAGCCCTACC | GCAGCAACCCTTCTGGAG | GTCCAGTTTTTTTTTTTTTTTGGTAG |
| viR14 | TTCCGCCCGCTCTCCCACCGCCTTT | CAGTTCCGCCCGCTCTC | GGTCCAGTTTTTTTTTTTTTTTAAAGG |
| miR-25 | CATTGCACTTGTCTCGGTCTGA | CATTGCACTTGTCTCGGT | GGTCCAGTTTTTTTTTTTTTTTCAGA |
| miR-93 | CAAAGTGCTGTTCGTGCAGGTAG | GCAAAGTGCTGTTCGTG | TCCAGTTTTTTTTTTTTTTTCTACCT |
| miR-106a | AAAAGTGCTTACAGTGCAGGTAGC | GAAAAGTGCTTACAGTGCAG | TCCAGTTTTTTTTTTTTTTTGCTAC |
| miR-26a | TTCAAGTAATCCAGGATAGGCT | GCAGTTCAAGTAATCCAGGA | TCCAGTTTTTTTTTTTTTTTAGCCT |
| miR-17-5p | CAAAGTGCTTACAGTGCAGGTAG | CAAAGTGCTTACAGTGCAG | GGTCCAGTTTTTTTTTTTTTTTCTAC |
