# Supplementary material for: The Role of Viral and Host MicroRNAs in the Aujeszky’s Disease Virus during the Infection Process
Source: PLoS One. 2014 Jan 24;9(1):e86965. doi: 10.1371/journal.pone.0086965 (PMC3901728; doi:10.1371/journal.pone.0086965)
Supplement: Table S3 — Complete in vitro miRNA profile from sequencing and differential expression analysis through fold change between NIA-3 strain infected group (NIA), Begonia strain infected group (BEG) and mock-infected group (MI). (DOCX) [file pone.0086965.s004.docx]

**Table S3. Complete *in vitro* miRNA profile from sequencing and differential expression analysis through fold change between NIA-3 strain infected group (NIA), Begonia strain infected group (BEG) and mock-infected group (MI).**

| **miRNA** | **Copy**  **number** | **IsomiRs** | **Fold Change BEG *vs*. MI** | **Fold Change NIA *vs*. MI** | **Fold Change NIA *vs*. BEG** |
| --- | --- | --- | --- | --- | --- |
| miR-23a-3p | 105,318 | 245 | 1.26 | 1.43 | 1.14 |
| miR-221-3p | 18,709 | 97 | 2.04 | 2.74 | 1.34 |
| miR-200b-3p | 16,910 | 94 | 1.68 | 3.13 | 1.86 |
| miR-23b-3p | 9,797 | 88 | 1.74 | 1.65 | -1.05 |
| miR-92a | 5,495 | 49 | 3.37 | -1.54 | -5.20 |
| miR-125b-5p | 4,896 | 35 | -7.37 | -8.36 | -1.13 |
| miR-378 | 4,733 | 56 | -1.16 | 1.27 | 1.48 |
| miR-10a-5p | 3,570 | 52 | -2.04 | -1.38 | 1.49 |
| miR-LLT1 | 3,280 | 30 | - | - | -4.04 |
| miR-183-5p | 2,622 | 40 | -1.06 | 1.46 | 1.55 |
| miR-99b-5p | 2,220 | 20 | -3.48 | -6.90 | -1.99 |
| miR-365-3p | 1,925 | 27 | -1.27 | -2.71 | -2.13 |
| miR-100 | 1,891 | 19 | -5.47 | -7.90 | -1.44 |
| miR-192-5p | 1,679 | 31 | -2.08 | -1.33 | 1.57 |
| miR-21-5p | 1,664 | 25 | 1.53 | 1.67 | 1.09 |
| miR-222-3p | 1,520 | 40 | -1.11 | -1.04 | 1.07 |
| miR-29a-5p | 1,250 | 12 | -2.44 | -1.66 | 1.47 |
| miR-1983 | 1,247 | 41 | 1.85 | 3.01 | 1.63 |
| miR-429 | 1,179 | 17 | 1.42 | 1.67 | 1.18 |
| miR-30d | 970 | 27 | -3.64 | -2.93 | 1.24 |
| miR-25-3p | 920 | 10 | -1.47 | -1.98 | -1.34 |
| miR-10b | 918 | 33 | -2.73 | -1.21 | 2.26 |
| miR-139-5p | 852 | 25 | -1.63 | -1.79 | -1.10 |
| miR-193a-5p | 777 | 15 | -2.97 | -1.78 | 1.67 |
| miR-200a-3p | 755 | 10 | -1.21 | 1.00 | 1.22 |
| miR-92b-3p | 704 | 33 | 2.18 | -2.62 | -5.70 |
| miR-186 | 689 | 16 | -2.90 | -2.15 | 1.35 |
| miR-324-3p | 663 | 34 | -1.52 | -1.46 | 1.04 |
| miR-2779 | 512 | 27 | -1.38 | -5.41 | -3.91 |
| miR-191-5p | 502 | 18 | -2.87 | -2.68 | 1.07 |
| miR-2887 | 479 | 13 | -20.24 | -203.79 | -10.07 |
| miR-15b-5p | 438 | 9 | -2.95 | -1.87 | 1.58 |
| miR-18a-5p | 437 | 15 | -1.95 | -2.08 | -1.07 |
| miR-34a | 425 | 14 | 1.32 | 2.17 | 1.64 |
| miR-rR1-5 | 415 | 10 | -4.09 | -2.74 | 1.49 |
| miR-2904 | 408 | 15 | -13.18 | -21.41 | -1.62 |
| miR-551a | 370 | 11 | -3.28 | -4.13 | -1.26 |
| miR-20a-5p | 366 | 11 | -1.24 | 1.35 | 1.68 |
| miR-16 | 365 | 13 | 1.07 | 1.29 | 1.21 |
| miR-125a-5p | 346 | 15 | -8.13 | -13.31 | -1.64 |
| miR-27a | 345 | 17 | -2.04 | -1.81 | 1.13 |
| miR-5109 | 294 | 20 | -9.38 | -18.18 | -1.94 |
| let-7a | 277 | 11 | -1.80 | -2.60 | -1.45 |
| miR-374b-5p | 261 | 4 | -1.79 | 1.09 | 1.95 |
| miR-29b-3p | 257 | 10 | -4.11 | -4.21 | -1.02 |
| miR-29c-5p | 245 | 18 | -2.35 | -2.24 | 1.05 |
| miR-4286 | 243 | 7 | -2.60 | -16.78 | -6.45 |
| miR-30a-5p | 226 | 12 | -7.87 | -9.07 | -1.15 |
| miR-24-3p | 221 | 12 | -2.50 | -1.68 | 1.48 |
| let-7b-5p | 209 | 16 | -3.64 | -5.76 | -1.58 |
| miR-574-5p | 209 | 11 | -3.09 | -2.27 | 1.36 |
| miR-26a-5p | 206 | 11 | -5.33 | -3.84 | 1.39 |
| miR-93-5p | 203 | 6 | -3.02 | -1.62 | 1.86 |
| miR-22-3p | 202 | 6 | -4.05 | -3.92 | 1.03 |
| miR-130b-3p | 182 | 12 | -2.98 | -2.17 | 1.37 |
| miR-505-3p | 181 | 10 | -2.00 | -2.84 | -1.42 |
| miR-500a-5p | 178 | 6 | 3.84 | 1.28 | -3.01 |
| miR-339-5p | 176 | 9 | -4.97 | -22.89 | -4.60 |
| miR-135b-5p | 173 | 8 | -1.23 | 1.11 | 1.36 |
| miR-151-3p | 172 | 10 | 1.24 | -1.06 | -1.32 |
| let-7d-3p | 171 | 9 | -1.36 | -8.66 | -6.38 |
| miR-320 | 171 | 16 | -2.40 | -2.93 | -1.22 |
| miR-30e-5p | 164 | 9 | -3.74 | -3.40 | 1.10 |
| miR-532-5p | 164 | 8 | 1.10 | -1.88 | -2.06 |
| miR-29a-3p | 160 | 5 | -1.36 | -1.53 | -1.13 |
| miR-374a-5p | 155 | 5 | -3.00 | -1.38 | 2.17 |
| miR-18a-3p | 147 | 11 | -1.28 | -2.00 | -1.56 |
| miR-19b | 144 | 4 | -6.97 | -4.30 | 1.62 |
| miR-532-3p | 144 | 6 | -1.49 | -3.13 | -2.09 |
| let-7f | 134 | 6 | 1.33 | 1.09 | -1.21 |
| miR-23a-5p | 126 | 6 | -11.83 | -27.63 | -2.34 |
| let-7i-5p | 122 | 11 | -5.40 | -6.29 | -1.17 |
| miR-652-3p | 122 | 6 | 1.54 | 1.42 | -1.09 |
| miR-505-5p | 117 | 9 | -15.46 | -25.93 | -1.68 |
| miR-17-5p | 110 | 6 | -1.14 | -1.05 | 1.09 |
| miR-184 | 109 | 5 | -4.25 | -1.86 | 2.28 |
| let-7e | 105 | 6 | -1.74 | -1.36 | 1.28 |
| miR-4454 | 104 | 11 | -3.32 | -7.51 | -2.26 |
| miR-361-5p | 102 | 9 | 2.83 | 3.16 | 1.12 |
| miR-2898 | 92 | 6 | -3.32 | -18.69 | -5.63 |
| miR-193a-3p | 89 | 5 | -3.06 | -14.68 | -4.80 |
| miR-30b-5p | 85 | 3 | 9.19 | 4.61 | -1.99 |
| miR-4497 | 82 | 9 | -3.63 | -13.83 | -3.81 |
| miR-6240 | 80 | 11 | 4.02 | -7.90 | -31.73 |
| miR-15b-3p | 79 | 9 | 1.48 | 2.41 | 1.63 |
| miR-2889 | 78 | 9 | -8.55 | -64.68 | -7.57 |
| miR-769-5p | 71 | 6 | -1.24 | 1.06 | 1.31 |
| miR-339-3p | 68 | 6 | -3.26 | -3.02 | 1.08 |
| miR-423-3p | 68 | 10 | -2.80 | -1.87 | 1.50 |
| miR-423-5p | 67 | 10 | -3.49 | -7.91 | -2.27 |
| miR-6529 | 66 | 3 | -4.41 | -2.34 | 1.89 |
| miR-92c | 65 | 7 | -1.31 | -4.37 | -3.32 |
| miR-128 | 62 | 6 | -2.35 | -1.59 | 1.48 |
| miR-324-5p | 59 | 4 | -7.55 | -6.65 | 1.14 |
| miR-335-3p | 59 | 6 | 2.80 | 2.05 | -1.36 |
| miR-500a-3p | 59 | 8 | -1.47 | 1.62 | 2.38 |
| miR-125a-3p | 57 | 11 | -5.74 | -5.19 | 1.10 |
| miR-99b-3p | 56 | 5 | -5.52 | -4.69 | 1.18 |
| miR-185 | 54 | 3 | -25.20 | -23.46 | 1.07 |
| let-7d-5p | 53 | 3 | -2.25 | -2.36 | -1.05 |
| miR-503 | 49 | 6 | -1.19 | -1.01 | 1.17 |
| miR-345-5p | 48 | 1 | -1.75 | -16.10 | -9.23 |
| miR-130b-5p | 47 | 5 | -3.04 | -1.99 | 1.53 |
| miR-27b-3p | 46 | 2 | -1.17 | -1.20 | -1.03 |
| let-7b-3p | 45 | 6 | -3.59 | -5.54 | -1.54 |
| miR-103 | 44 | 6 | -4.66 | -8.21 | -1.76 |
| miR-664-5p | 44 | 3 | 1.46 | 3.06 | 2.09 |
| miR-148b-3p | 41 | 3 | -17.32 | -14.09 | 1.23 |
| miR-191-3p | 40 | 5 | -1.86 | -1.62 | 1.15 |
| miR-502b | 40 | 2 | -1.87 | -1.38 | 1.35 |
| miR-LLT11a | 39 | 2 | - | - | 1.47 |
| miR-155-5p | 36 | 6 | -1.62 | 1.47 | 2.38 |
| miR-301a-3p | 35 | 3 | -3.21 | -1.70 | 1.89 |
| miR-484 | 35 | 4 | -10.32 | -40.50 | -3.92 |
| miR-152 | 34 | 4 | -13.19 | -3.91 | 3.37 |
| miR-106b-3p | 32 | 4 | -3.36 | -3.80 | -1.13 |
| miR-126-5p | 30 | 1 | 1.01 | 1.97 | 1.95 |
| miR-200a-5p | 29 | 6 | 1.15 | -2.90 | -3.34 |
| miR-362-3p | 29 | 2 | 6.71 | 6.47 | -1.04 |
| miR-425-3p | 29 | 6 | -5.92 | -11.31 | -1.91 |
| miR-1260 | 28 | 3 | -1.24 | -31.05 | -24.97 |
| miR-17-3p | 28 | 3 | -2.35 | -4.09 | -1.74 |
| miR-30c-5p | 28 | 2 | -5.69 | -4.58 | 1.24 |
| miR-716b | 28 | 5 | -14.78 | -8.65 | 1.71 |
| miR-140-3p | 27 | 3 | 1.65 | 1.40 | -1.18 |
| miR-378a-5p | 27 | 2 | - | - | -4.02 |
| miR-28-3p | 26 | 3 | -4.68 | -7.31 | -1.56 |
| miR-126-3p | 23 | 2 | 4.60 | 4.96 | 1.08 |
| miR-362-5p | 23 | 4 | -5.90 | -1.50 | 3.93 |
| miR-615 | 23 | 2 | -4.89 | -6.36 | -1.30 |
| miR-200c-3p | 21 | 2 | - | - | -1.05 |
| miR-LLT6 | 21 | 3 | - | - | 1.09 |
| miR-1306-5p | 20 | 3 | -4.41 | -7.01 | -1.59 |
| miR-25-5p | 19 | 3 | -31.70 | -15.48 | 2.05 |
| miR-140-5p | 17 | 2 | -11.17 | -4.14 | 2.70 |
| miR-30a-3p | 17 | 3 | -2.12 | 1.01 | 2.15 |
| miR-6651-5p | 17 | 3 | -1.77 | 4.18 | 7.39 |
| let-7g | 16 | 3 | -22.01 | -5.37 | 4.10 |
| miR-340-5p | 16 | 3 | -1.18 | -1.53 | -1.30 |
| miR-744 | 16 | 2 | - | - | -1.73 |
| miR-23b-5p | 15 | 3 | -11.25 | -13.73 | -1.22 |
| miR-29c-3p | 15 | 1 | -16.76 | -4.98 | 3.37 |
| miR-106b-5p | 14 | 2 | -3.92 | -1.64 | 2.39 |
| miR-130a | 14 | 2 | -9.91 | -2.29 | 4.34 |
| miR-421-3p | 14 | 1 | -8.21 | -2.62 | 3.13 |
| miR-106a | 13 | 2 | 1.34 | -1.06 | -1.41 |
| miR-1386 | 13 | 2 | -11.43 | -12.60 | -1.10 |
| miR-3184-5p | 13 | 2 | 1.55 | 1.14 | -1.36 |
| miR-LLT2 | 13 | 2 | - | - | -6.75 |
| miR-1285 | 12 | 2 | - | - | -2.78 |
| miR-196b-5p | 12 | 2 | -24.73 | -6.04 | 4.10 |
| miR-19a | 12 | 1 | -2.93 | 1.08 | 3.16 |
| miR-3195 | 12 | 3 | -40.58 | -30.11 | 1.35 |
| miR-361-3p | 12 | 2 | -1.18 | 1.15 | 1.36 |
| miR-455-5p | 12 | 2 | -2.71 | -3.25 | -1.20 |
| miR-5097 | 11 | 3 | 1.02 | -2.20 | -2.25 |
| miR-5105 | 11 | 2 | -2.95 | - | - |
| miR-101 | 10 | 2 | - | -1.32 | - |
| miR-2320-3p | 10 | 2 | - | - | 2.71 |
| miR-LLT8 | 10 | 1 | - | - | 3.09 |
| miR-1249 | 9 | 2 | 4.18 | -2.68 | -11.20 |
| miR-125b-2-3p | 9 | 2 | -1.78 | -2.62 | -1.47 |
| miR-192-3p | 9 | 2 | 2.02 | -2.63 | -5.31 |
| miR-210 | 9 | 3 | -1.42 | 2.70 | 3.84 |
| miR-26a-1-3p | 9 | 1 | -1.01 | 2.32 | 2.35 |
| miR-331-5p | 9 | 1 | - | - | -2.84 |
| miR-466i-5p | 9 | 3 | -22.92 | -8.54 | 2.68 |
| miR-1271-3p | 8 | 2 | 1.70 | -2.62 | -4.45 |
| miR-151-5p | 8 | 1 | -4.25 | -5.21 | -1.23 |
| miR-183-3p | 8 | 2 | -11.14 | - | - |
| miR-200b-5p | 8 | 1 | -3.53 | -1.09 | 3.25 |
| miR-215 | 8 | 1 | - | - | -3.53 |
| miR-26b-5p | 8 | 2 | - | - | 2.04 |
| miR-31-3p | 8 | 1 | 1.69 | 1.54 | -1.10 |
| miR-31-5p | 8 | 1 | -2.05 | -7.83 | -3.82 |
| miR-328 | 8 | 2 | 1.67 | -2.62 | -4.37 |
| miR-499-5p | 8 | 1 | - | - | 1.36 |
| miR-316-3p | 7 | 1 | - | - | -3.57 |
| miR-335-5p | 7 | 1 | 1.27 | 1.14 | -1.11 |
| miR-425-5p | 7 | 2 | -2.03 | -1.75 | 1.16 |
| miR-455-3p | 7 | 1 | -8.78 | -1.32 | 6.66 |
| miR-1458 | 6 | 2 | -2.36 | -5.18 | -2.20 |
| miR-15a | 6 | 1 | -1.01 | 1.14 | 1.16 |
| miR-181b | 6 | 1 | - | - | 2.02 |
| miR-195 | 6 | 1 | - | - | - |
| miR-296-5p | 6 | 2 | -4.27 | -3.97 | 1.07 |
| miR-3074-5p | 6 | 1 | - | - | -2.08 |
| miR-32 | 6 | 1 | - | - | 1.17 |
| miR-33b-3p | 6 | 1 | -6.16 | -4.51 | 1.37 |
| miR-490 | 6 | 1 | -3.53 | -2.62 | 1.35 |
| miR-101b-5p | 5 | 1 | - | -10.35 | - |
| miR-2310 | 5 | 1 | -6.16 | -9.02 | -1.47 |
| miR-338-5p | 5 | 1 | - | - | 2.00 |
| miR-3586-3p | 5 | 1 | 1.13 | - | - |
| miR-450b-5p | 5 | 1 | - | - | 1.18 |
| miR-7-5p | 5 | 1 | -1.28 | - | - |
| miR-760 | 5 | 1 | - | - | -1.10 |
| miR-107-3p | 4 | 1 | - | - | - |
| miR-107-5p | 4 | 1 | - | - | - |
| miR-219-1-3p | 4 | 1 | -3.53 | - | - |
| miR-23c | 4 | 1 | - | - | 1.62 |
| miR-301b | 4 | 1 | -3.53 | -2.59 | 1.37 |
| miR-450c-5p | 4 | 1 | - | - | -3.64 |
| miR-103a-5p | 3 | 1 | 1.17 | - | - |
| miR-10a-3p | 3 | 1 | - | - | 1.32 |
| miR-1246 | 3 | 1 | - | -2.59 | - |
| miR-1296-5p | 3 | 1 | - | -1.32 | - |
| miR-138-5p | 3 | 1 | - | - | - |
| miR-149 | 3 | 1 | -7.07 | - | - |
| miR-194-5p | 3 | 1 | - | -5.18 | - |
| miR-222-5p | 3 | 1 | - | -5.18 | - |
| miR-22-5p | 3 | 1 | -3.53 | -2.68 | 1.32 |
| miR-2411 | 3 | 1 | - | - | 2.73 |
| miR-24-5p | 3 | 1 | 1.41 | - | - |
| miR-2478 | 3 | 1 | 1.89 | - | - |
| miR-26a-2-3p | 3 | 1 | - | - | -1.47 |
| miR-29b-1-5p | 3 | 1 | - | -2.59 | - |
| miR-29b-2-5p | 3 | 1 | - | - | -2.55 |
| miR-330-3p | 3 | 1 | - | -5.18 | - |
| miR-3613-3p | 3 | 1 | -8.78 | -6.44 | 1.37 |
| miR-4485 | 3 | 1 | - | - | - |
| miR-454 | 3 | 1 | - | - | -1.47 |
| miR-539-5p | 3 | 1 | - | - | 1.35 |
| miR-545-3p | 3 | 1 | - | -3.27 | - |
| miR-574-3p | 3 | 1 | -1.01 | - | - |
| miR-6243 | 3 | 1 | - | - | - |
| miR-652-5p | 3 | 1 | - | - | -1.82 |
| miR-660-5p | 3 | 1 | - | -5.18 | - |
| miR-LLT9 | 3 | 1 | - | - | - |
