# Supplementary material for: The Role of Viral and Host MicroRNAs in the Aujeszky’s Disease Virus during the Infection Process
Source: PLoS One. 2014 Jan 24;9(1):e86965. doi: 10.1371/journal.pone.0086965 (PMC3901728; doi:10.1371/journal.pone.0086965)
Supplement: Table S4 — Complete in vivo miRNA profile from sequencing data and differential expression analysis through fold change between NIA-3 strain infected group (NIA), Begonia strain infected group (BEG) and healthy group (HE). (DOCX) [file pone.0086965.s005.docx]

**Table S4. Complete *in vivo* miRNA profile from sequencing data and differential expression analysis through fold change between NIA-3 strain infected group (NIA) and Begonia strain infected group (BEG).**

| **miRNA** | **Copy number** | **IsomiRs** | **Fold Change NIA *vs*. BEG** |
| --- | --- | --- | --- |
| miR-125b-5p | 57,400 | 171 | -1.62 |
| miR-23b-3p | 21,832 | 114 | -1.02 |
| miR-200b-3p | 17,544 | 92 | 3.71 |
| miR-99a-5p | 11,178 | 76 | -1.38 |
| miR-23a-3p | 10,690 | 74 | 1.30 |
| miR-92a | 6,689 | 62 | 2.02 |
| miR-125a-5p | 5,560 | 84 | -2.57 |
| miR-100 | 5,044 | 34 | -1.30 |
| miR-338-5p | 4,766 | 28 | 1.32 |
| miR-183-5p | 4,598 | 44 | -1.18 |
| miR-200c-3p | 4,354 | 32 | 3.11 |
| miR-126-5p | 3,945 | 26 | 2.03 |
| miR-124a-3p | 3,810 | 63 | 2.03 |
| miR-126-3p | 3,251 | 37 | -1.25 |
| miR-138-5p | 3,017 | 44 | -3.66 |
| miR-139-5p | 3,002 | 27 | -1.66 |
| miR-539-5p | 2,991 | 34 | -3.87 |
| miR-99b-5p | 2,900 | 30 | -1.25 |
| miR-30d | 2,324 | 40 | 1.25 |
| miR-133a | 1,990 | 35 | 107.79 |
| miR-218b | 1,887 | 25 | -2.54 |
| miR-9-3p | 1,620 | 32 | -1.61 |
| miR-129a | 1,566 | 35 | -1.25 |
| miR-429 | 1,475 | 25 | 4.64 |
| miR-128 | 1,432 | 45 | -1.90 |
| miR-26a-5p | 1,374 | 21 | 2.79 |
| let-7b-5p | 1,271 | 26 | 1.82 |
| miR-200a-3p | 1,182 | 23 | 2.15 |
| miR-92b-3p | 1,173 | 24 | -1.60 |
| miR-29c-5p | 1,007 | 23 | -1.13 |
| miR-25-3p | 987 | 13 | 1.76 |
| let-7a | 953 | 15 | 4.62 |
| miR-324-3p | 947 | 39 | -1.24 |
| miR-378 | 923 | 25 | 5.07 |
| miR-150 | 917 | 15 | 1.17 |
| miR-451 | 867 | 11 | -1.23 |
| miR-191-5p | 795 | 15 | -2.37 |
| miR-145-5p | 773 | 28 | 1.18 |
| miR-324-5p | 772 | 9 | -3.35 |
| miR-874-3p | 768 | 23 | -1.46 |
| let-7d-3p | 668 | 17 | -1.59 |
| miR-486 | 637 | 15 | -1.13 |
| miR-3065-3p | 633 | 24 | -1.15 |
| miR-409-3p | 629 | 29 | -1.47 |
| miR-500a-5p | 578 | 15 | -1.76 |
| miR-30e-5p | 564 | 18 | 1.29 |
| miR-9-5p | 548 | 15 | 1.91 |
| miR-365-3p | 541 | 14 | 1.80 |
| miR-204 | 534 | 17 | -1.18 |
| miR-206 | 512 | 8 | 648.10 |
| miR-181a-5p | 500 | 12 | -1.26 |
| miR-129b | 449 | 7 | -1.82 |
| let-7c | 432 | 12 | 1.05 |
| miR-29a-5p | 419 | 12 | -1.86 |
| miR-221-3p | 417 | 10 | -3.09 |
| miR-532-3p | 412 | 9 | -2.30 |
| miR-409-5p | 395 | 8 | 1.03 |
| miR-329 | 364 | 11 | 1.14 |
| miR-551b-3p | 356 | 9 | -1.13 |
| miR-187 | 321 | 18 | -1.21 |
| miR-181c-5p | 318 | 11 | -1.32 |
| miR-149 | 314 | 13 | -1.15 |
| miR-27b-3p | 286 | 6 | -1.21 |
| miR-186 | 280 | 9 | -1.05 |
| let-7d-5p | 278 | 6 | 1.48 |
| miR-320 | 261 | 18 | -3.26 |
| miR-485-5p | 261 | 10 | -1.75 |
| miR-21-5p | 252 | 6 | 1.14 |
| miR-1249 | 247 | 8 | -5.23 |
| miR-374a-5p | 238 | 5 | 1.11 |
| miR-676-3p | 234 | 8 | 1.46 |
| miR-133b | 228 | 10 | 87.89 |
| miR-1224-5p | 226 | 16 | -2.40 |
| miR-328 | 219 | 11 | -1.76 |
| let-7i-5p | 218 | 8 | 2.26 |
| miR-193b-3p | 198 | 11 | -1.34 |
| miR-532-5p | 198 | 7 | -1.83 |
| miR-137 | 189 | 8 | -6.39 |
| miR-15b-5p | 189 | 3 | 2.05 |
| miR-151-3p | 185 | 11 | 1.43 |
| miR-29b-3p | 183 | 6 | 1.22 |
| miR-16 | 182 | 8 | -1.50 |
| miR-664-5p | 181 | 6 | -2.84 |
| miR-193a-5p | 179 | 5 | 1.85 |
| miR-1983 | 177 | 13 | -1.20 |
| miR-10b | 170 | 12 | -1.18 |
| miR-127 | 154 | 19 | 1.69 |
| let-7b-3p | 151 | 7 | -3.53 |
| miR-181a-3p | 151 | 4 | 1.84 |
| miR-24-3p | 145 | 9 | -1.79 |
| miR-423-5p | 144 | 15 | -1.24 |
| miR-551a | 136 | 7 | -2.64 |
| miR-181b | 132 | 9 | -2.07 |
| miR-30a-5p | 130 | 6 | 1.49 |
| miR-1247-5p | 120 | 13 | -4.52 |
| miR-20b | 120 | 5 | -2.69 |
| miR-345-5p | 119 | 4 | -1.08 |
| miR-652-3p | 111 | 4 | 1.21 |
| miR-361-5p | 109 | 7 | 4.29 |
| miR-363 | 108 | 7 | -2.17 |
| let-7f | 106 | 5 | 4.16 |
| miR-34a | 97 | 6 | -2.80 |
| miR-99a-3p | 97 | 5 | -4.55 |
| miR-299-5p | 96 | 5 | 2.73 |
| miR-93-5p | 92 | 7 | -3.31 |
| miR-154b | 91 | 3 | -3.01 |
| miR-125b-2-3p | 90 | 13 | -1.45 |
| miR-483-3p | 89 | 5 | -5.64 |
| miR-152 | 84 | 6 | 2.00 |
| miR-342 | 84 | 4 | 2.35 |
| let-7e | 83 | 5 | 2.66 |
| miR-338-3p | 82 | 4 | -5.04 |
| miR-182 | 81 | 6 | 3.61 |
| miR-1468 | 80 | 3 | 1.32 |
| miR-22-3p | 80 | 6 | 1.64 |
| miR-29a-3p | 79 | 4 | 1.10 |
| miR-339-5p | 79 | 7 | 4.40 |
| miR-769-5p | 79 | 6 | -1.54 |
| miR-495-3p | 78 | 5 | 1.03 |
| miR-214-5p | 75 | 5 | -2.25 |
| miR-200a-5p | 73 | 5 | 9.38 |
| miR-181c-3p | 73 | 5 | 1.33 |
| miR-20a-5p | 72 | 5 | -1.16 |
| miR-374b-5p | 72 | 4 | -1.23 |
| miR-484 | 68 | 7 | 1.03 |
| miR-4454 | 67 | 5 | -1.03 |
| miR-362-5p | 64 | 5 | 1.70 |
| miR-19b | 64 | 6 | 1.52 |
| miR-140-5p | 63 | 7 | -3.01 |
| miR-199a-5p | 63 | 5 | -2.05 |
| miR-199a-3p | 62 | 8 | -1.24 |
| miR-325 | 62 | 3 | -1.31 |
| miR-183-3p | 60 | 8 | 1.23 |
| miR-758 | 60 | 6 | -2.28 |
| miR-670-5p | 58 | 3 | 1.56 |
| miR-29c-3p | 57 | 3 | 1.26 |
| miR-2483 | 56 | 5 | -1.07 |
| miR-28-3p | 56 | 6 | -1.34 |
| miR-3065-5p | 55 | 2 | -6.42 |
| miR-6529 | 55 | 3 | -1.71 |
| miR-377-5p | 54 | 5 | 1.02 |
| miR-505-5p | 53 | 3 | -1.71 |
| miR-199b-3p | 51 | 6 | -1.05 |
| miR-574-3p | 51 | 2 | -6.25 |
| miR-99b-3p | 50 | 5 | -3.63 |
| miR-1-3p | 49 | 3 | 57.10 |
| miR-4286 | 49 | 8 | -2.74 |
| miR-504 | 48 | 3 | -1.02 |
| miR-505-3p | 47 | 7 | 3.29 |
| miR-425-3p | 46 | 5 | -1.41 |
| miR-30c-5p | 43 | 4 | 2.03 |
| miR-502-3p | 42 | 5 | -1.87 |
| miR-195 | 42 | 4 | 3.16 |
| miR-2898 | 42 | 5 | -6.29 |
| miR-105-5p | 41 | 6 | -1.85 |
| miR-574-5p | 41 | 6 | -4.13 |
| miR-543 | 39 | 5 | -1.79 |
| miR-1306-5p | 37 | 4 | -1.30 |
| miR-15a | 36 | 2 | 1.60 |
| miR-219-5p | 36 | 4 | -9.98 |
| miR-106b-3p | 35 | 2 | -4.45 |
| miR-130a | 35 | 5 | 2.30 |
| miR-18a-5p | 35 | 5 | 1.20 |
| miR-331-5p | 35 | 5 | 1.19 |
| miR-30b-5p | 34 | 1 | 2.98 |
| miR-130b-3p | 34 | 4 | 1.20 |
| miR-140-3p | 34 | 4 | 1.04 |
| miR-329a | 34 | 7 | 1.64 |
| miR-455-5p | 34 | 3 | -4.50 |
| miR-1343 | 33 | 4 | -4.23 |
| miR-135b-5p | 32 | 2 | 1.84 |
| miR-125a-3p | 31 | 3 | -1.31 |
| miR-101 | 30 | 7 | 1.47 |
| miR-27a | 30 | 3 | 1.28 |
| miR-222-3p | 28 | 4 | -2.07 |
| miR-3613-3p | 28 | 3 | -2.23 |
| miR-497 | 28 | 4 | -2.56 |
| miR-500a-3p | 27 | 5 | -1.47 |
| miR-503 | 27 | 5 | -2.13 |
| miR-499-5p | 26 | 2 | - |
| miR-193a-3p | 25 | 2 | -1.75 |
| miR-5109 | 25 | 5 | -2.42 |
| miR-151-5p | 24 | 1 | 1.80 |
| miR-378a-5p | 24 | 1 | 2.16 |
| miR-542-5p | 24 | 3 | -3.92 |
| miR-132-3p | 24 | 3 | -1.04 |
| miR-2779 | 23 | 4 | -6.87 |
| miR-330-5p | 23 | 2 | 1.69 |
| miR-103 | 22 | 3 | -1.32 |
| let-7g | 22 | 2 | 2.25 |
| miR-18a-3p | 21 | 4 | -5.02 |
| miR-4324 | 21 | 4 | -7.81 |
| miR-330-3p | 21 | 3 | 4.31 |
| miR-490 | 21 | 3 | -1.33 |
| miR-760 | 21 | 5 | -1.79 |
| miR-26b-5p | 21 | 3 | 1.24 |
| miR-369-3p | 21 | 1 | 4.52 |
| miR-361-3p | 20 | 4 | -13.12 |
| miR-135a-5p | 20 | 2 | -1.15 |
| miR-545-3p | 20 | 3 | 1.94 |
| miR-143-3p | 20 | 3 | 3.33 |
| miR-132-5p | 19 | 3 | 6.29 |
| miR-211 | 19 | 3 | -2.27 |
| miR-421-3p | 19 | 2 | -1.11 |
| miR-326 | 18 | 1 | -3.51 |
| miR-381 | 18 | 3 | -1.26 |
| miR-138-3p | 17 | 2 | 3.22 |
| miR-23b-5p | 17 | 4 | -1.66 |
| miR-106b-5p | 17 | 2 | 2.42 |
| miR-185 | 16 | 1 | 1.30 |
| miR-423-3p | 16 | 3 | 1.58 |
| miR-148b-3p | 15 | 2 | 2.79 |
| miR-4497 | 15 | 1 | -1.46 |
| miR-29b-2-5p | 14 | 3 | -1.09 |
| miR-32 | 14 | 1 | -1.00 |
| miR-335-3p | 14 | 3 | 3.66 |
| miR-592 | 14 | 1 | 3.92 |
| miR-124a-5p | 13 | 2 | 1.27 |
| miR-154c | 13 | 3 | -1.61 |
| miR-3956-3p | 12 | 3 | 2.39 |
| miR-716b | 11 | 3 | 7.38 |
| miR-130b-5p | 11 | 2 | 2.73 |
| miR-141 | 11 | 3 | 2.71 |
| miR-382-5p | 11 | 2 | 2.91 |
| miR-412-3p | 11 | 3 | -1.83 |
| miR-376c-3p | 10 | 1 | 1.11 |
| miR-425-5p | 10 | 2 | - |
| miR-192-5p | 9 | 2 | 1.30 |
| miR-96-5p | 9 | 2 | 1.39 |
| miR-LLT1 | 9 | 1 | - |
| miR-1298 | 8 | 2 | 1.06 |
| miR-301a-3p | 8 | 2 | -1.64 |
| miR-17-5p | 8 | 2 | 2.48 |
| miR-3586-3p | 8 | 1 | 4.69 |
| miR-744 | 8 | 2 | -9.16 |
| miR-885-5p | 8 | 1 | 4.04 |
| miR-450b-5p | 8 | 1 | 1.07 |
| miR-122 | 7 | 1 | 1.91 |
| miR-2904 | 7 | 2 | -1.03 |
| miR-181d-5p | 7 | 2 | -1.05 |
| miR-5100 | 7 | 2 | -1.26 |
| miR-346 | 7 | 1 | -3.25 |
| miR-339-3p | 6 | 2 | 10.17 |
| miR-3576 | 6 | 1 | 5.03 |
| miR-7-5p | 6 | 1 | -7.75 |
| miR-146a-5p | 6 | 1 | -2.21 |
| miR-191-3p | 6 | 2 | -1.14 |
| miR-193b-5p | 6 | 1 | -1.15 |
| miR-200b-5p | 6 | 2 | - |
| miR-3184-5p | 6 | 2 | - |
| miR-345-3p | 6 | 1 | -1.42 |
| miR-935 | 6 | 1 | -2.13 |
| miR-3957-3p | 6 | 2 | -1.72 |
| miR-1296-5p | 6 | 2 | - |
| miR-7-3p | 6 | 1 | - |
| miR-1271-3p | 5 | 1 | -1.67 |
| miR-30e-3p | 5 | 1 | 2.11 |
| miR-432-5p | 5 | 1 | -2.85 |
| miR-450c-5p | 5 | 1 | -1.71 |
| miR-488-3p | 5 | 1 | -2.41 |
| miR-26b-3p | 5 | 1 | - |
| miR-146b | 5 | 1 | - |
| miR-375 | 5 | 1 | - |
| miR-3596 | 4 | 1 | 1.24 |
| miR-450a | 4 | 1 | 1.64 |
| miR-489 | 4 | 1 | -1.08 |
| miR-656 | 4 | 1 | -4.45 |
| miR-1260 | 4 | 1 | - |
| miR-148a-3p | 4 | 1 | -1.35 |
| miR-219-3p | 4 | 1 | - |
| miR-30a-3p | 4 | 1 | -2.70 |
| miR-362-3p | 4 | 1 | - |
| miR-93-3p | 4 | 1 | - |
| miR-125b-1-3p | 4 | 1 | - |
| miR-106a | 3 | 1 | -1.25 |
| miR-148a-5p | 3 | 1 | -1.51 |
| miR-2887 | 3 | 1 | 1.77 |
| miR-340-5p | 3 | 1 | 1.84 |
| miR-545-5p | 3 | 1 | 2.25 |
| miR-6651-5p | 3 | 1 | 1.08 |
| miR-874-5p | 3 | 1 | -3.16 |
| miR-1-5p | 3 | 1 | - |
| miR-1912 | 3 | 1 | - |
| miR-1271-5p | 3 | 1 | - |
| miR-1285 | 3 | 1 | - |
| miR-2320-3p | 3 | 1 | - |
| miR-23a-5p | 3 | 1 | - |
| miR-31-3p | 3 | 1 | - |
| miR-370 | 3 | 1 | - |
| miR-323 | 3 | 1 | - |
| miR-384 | 3 | 1 | - |
| miR-3587 | 3 | 1 | - |
| miR-369-5p | 3 | 1 | - |
| miR-485-3p | 3 | 1 | - |
| miR-380 | 3 | 1 | - |
| miR-410 | 3 | 1 | - |
| miR-411-5p | 3 | 1 | - |
| miR-487b | 3 | 1 | - |
| miR-668-3p | 3 | 1 | - |
| miR-664-3p | 3 | 1 | - |
| miR-885-3p | 3 | 1 | - |
| miR-98 | 3 | 1 | - |
