# Supplementary material for: The Role of Viral and Host MicroRNAs in the Aujeszky’s Disease Virus during the Infection Process
Source: PLoS One. 2014 Jan 24;9(1):e86965. doi: 10.1371/journal.pone.0086965 (PMC3901728; doi:10.1371/journal.pone.0086965)
Supplement: Table S5 — Described miRNAs in only one approach: cell culture (CC) or infected tissue (IT). (DOCX) [file pone.0086965.s006.docx]

**Table S5. Described miRNAs in only one approach: cell culture (CC) or infected tissue (IT).**

| **miRNA** | **Copy number** | **IsomiRs** | **Approach** |
| --- | --- | --- | --- |
| miR-99a-5p | 11,178 | 76 | IT |
| miR-124a-3p | 3,810 | 63 | IT |
| miR-10a-5p | 3,570 | 52 | CC |
| miR-133a | 1,990 | 35 | IT |
| miR-218b | 1,887 | 25 | IT |
| miR-9-3p | 1,620 | 32 | IT |
| miR-129a | 1,566 | 35 | IT |
| miR-150 | 917 | 15 | IT |
| miR-451 | 867 | 11 | IT |
| miR-145-5p | 773 | 28 | IT |
| miR-874-3p | 768 | 23 | IT |
| miR-486 | 637 | 15 | IT |
| miR-3065-3p | 633 | 24 | IT |
| miR-409-3p | 629 | 29 | IT |
| miR-9-5p | 548 | 15 | IT |
| miR-204 | 534 | 17 | IT |
| miR-206 | 512 | 8 | IT |
| miR-181a-5p | 500 | 12 | IT |
| miR-129b | 449 | 7 | IT |
| let-7c | 432 | 12 | IT |
| miR-rR1-5 | 415 | 10 | CC |
| miR-409-5p | 395 | 8 | IT |
| miR-329 | 364 | 11 | IT |
| miR-551b-3p | 356 | 9 | IT |
| miR-187 | 321 | 18 | IT |
| miR-181c-5p | 318 | 11 | IT |
| miR-485-5p | 261 | 10 | IT |
| miR-676-3p | 234 | 8 | IT |
| miR-133b | 228 | 10 | IT |
| miR-1224-5p | 226 | 16 | IT |
| miR-193b-3p | 198 | 11 | IT |
| miR-137 | 189 | 8 | IT |
| miR-127 | 154 | 19 | IT |
| miR-181a-3p | 151 | 4 | IT |
| miR-1247-5p | 120 | 13 | IT |
| miR-20b | 120 | 5 | IT |
| miR-184 | 109 | 5 | CC |
| miR-363 | 108 | 7 | IT |
| miR-99a-3p | 97 | 5 | IT |
| miR-299-5p | 96 | 5 | IT |
| miR-154b | 91 | 3 | IT |
| miR-483-3p | 89 | 5 | IT |
| miR-342 | 84 | 4 | IT |
| miR-338-3p | 82 | 4 | IT |
| miR-182 | 81 | 6 | IT |
| miR-1468 | 80 | 3 | IT |
| miR-6240 | 80 | 11 | CC |
| miR-15b-3p | 79 | 9 | CC |
| miR-2889 | 78 | 9 | CC |
| miR-495-3p | 78 | 5 | IT |
| miR-214-5p | 75 | 5 | IT |
| miR-181c-3p | 73 | 5 | IT |
| miR-92c | 65 | 7 | CC |
| miR-199a-5p | 63 | 5 | IT |
| miR-199a-3p | 62 | 8 | IT |
| miR-325 | 62 | 3 | IT |
| miR-758 | 60 | 6 | IT |
| miR-670-5p | 58 | 3 | IT |
| miR-2483 | 56 | 5 | IT |
| miR-3065-5p | 55 | 2 | IT |
| miR-377-5p | 54 | 5 | IT |
| miR-199b-3p | 51 | 6 | IT |
| miR-1-3p | 49 | 3 | IT |
| miR-504 | 48 | 3 | IT |
| miR-502-3p | 42 | 5 | IT |
| miR-105-5p | 41 | 6 | IT |
| miR-502b | 40 | 2 | CC |
| miR-543 | 39 | 5 | IT |
| miR-LLT11a | 39 | 2 | CC |
| miR-155-5p | 36 | 6 | CC |
| miR-219-5p | 36 | 4 | IT |
| miR-329a | 34 | 7 | IT |
| miR-1343 | 33 | 4 | IT |
| miR-17-3p | 28 | 3 | CC |
| miR-497 | 28 | 4 | IT |
| miR-132-3p | 24 | 3 | IT |
| miR-542-5p | 24 | 3 | IT |
| miR-330-5p | 23 | 2 | IT |
| miR-615 | 23 | 2 | CC |
| miR-369-3p | 21 | 1 | IT |
| miR-4324 | 21 | 4 | IT |
| miR-LLT6 | 21 | 3 | CC |
| miR-135a-5p | 20 | 2 | IT |
| miR-143-3p | 20 | 3 | IT |
| miR-132-5p | 19 | 3 | IT |
| miR-211 | 19 | 3 | IT |
| miR-25-5p | 19 | 3 | CC |
| miR-326 | 18 | 1 | IT |
| miR-381 | 18 | 3 | IT |
| miR-138-3p | 17 | 2 | IT |
| miR-592 | 14 | 1 | IT |
| miR-124a-5p | 13 | 2 | IT |
| miR-1386 | 13 | 2 | CC |
| miR-154c | 13 | 3 | IT |
| miR-LLT2 | 13 | 2 | CC |
| miR-196b-5p | 12 | 2 | CC |
| miR-19a | 12 | 1 | CC |
| miR-3195 | 12 | 3 | CC |
| miR-3956-3p | 12 | 3 | IT |
| miR-141 | 11 | 3 | IT |
| miR-382-5p | 11 | 2 | IT |
| miR-412-3p | 11 | 3 | IT |
| miR-5097 | 11 | 3 | CC |
| miR-5105 | 11 | 2 | CC |
| miR-376c-3p | 10 | 1 | IT |
| miR-LLT8 | 10 | 1 | CC |
| miR-192-3p | 9 | 2 | CC |
| miR-210 | 9 | 3 | CC |
| miR-26a-1-3p | 9 | 1 | CC |
| miR-466i-5p | 9 | 3 | CC |
| miR-96-5p | 9 | 2 | IT |
| miR-1298 | 8 | 2 | IT |
| miR-215 | 8 | 1 | CC |
| miR-31-5p | 8 | 1 | CC |
| miR-885-5p | 8 | 1 | IT |
| miR-122 | 7 | 1 | IT |
| miR-181d-5p | 7 | 2 | IT |
| miR-316-3p | 7 | 1 | CC |
| miR-335-5p | 7 | 1 | CC |
| miR-346 | 7 | 1 | IT |
| miR-455-3p | 7 | 1 | CC |
| miR-5100 | 7 | 2 | IT |
| miR-1458 | 6 | 2 | CC |
| miR-146a-5p | 6 | 1 | IT |
| miR-193b-5p | 6 | 1 | IT |
| miR-296-5p | 6 | 2 | CC |
| miR-3074-5p | 6 | 1 | CC |
| miR-33b-3p | 6 | 1 | CC |
| miR-345-3p | 6 | 1 | IT |
| miR-3576 | 6 | 1 | IT |
| miR-3957-3p | 6 | 2 | IT |
| miR-7-3p | 6 | 1 | IT |
| miR-935 | 6 | 1 | IT |
| miR-101b-5p | 5 | 1 | CC |
| miR-146b | 5 | 1 | IT |
| miR-2310 | 5 | 1 | CC |
| miR-26b-3p | 5 | 1 | IT |
| miR-30e-3p | 5 | 1 | IT |
| miR-375 | 5 | 1 | IT |
| miR-432-5p | 5 | 1 | IT |
| miR-488-3p | 5 | 1 | IT |
| miR-107-3p | 4 | 1 | CC |
| miR-107-5p | 4 | 1 | CC |
| miR-125b-1-3p | 4 | 1 | IT |
| miR-148a-3p | 4 | 1 | IT |
| miR-219-1-3p | 4 | 1 | CC |
| miR-219-3p | 4 | 1 | IT |
| miR-23c | 4 | 1 | CC |
| miR-301b | 4 | 1 | CC |
| miR-3596 | 4 | 1 | IT |
| miR-450a | 4 | 1 | IT |
| miR-489 | 4 | 1 | IT |
| miR-656 | 4 | 1 | IT |
| miR-93-3p | 4 | 1 | IT |
| miR-103a-5p | 3 | 1 | CC |
| miR-10a-3p | 3 | 1 | CC |
| miR-1246 | 3 | 1 | CC |
| miR-1271-5p | 3 | 1 | IT |
| miR-148a-5p | 3 | 1 | IT |
| miR-1-5p | 3 | 1 | IT |
| miR-1912 | 3 | 1 | IT |
| miR-194-5p | 3 | 1 | CC |
| miR-222-5p | 3 | 1 | CC |
| miR-22-5p | 3 | 1 | CC |
| miR-2411 | 3 | 1 | CC |
| miR-24-5p | 3 | 1 | CC |
| miR-2478 | 3 | 1 | CC |
| miR-26a-2-3p | 3 | 1 | CC |
| miR-29b-1-5p | 3 | 1 | CC |
| miR-323 | 3 | 1 | IT |
| miR-3587 | 3 | 1 | IT |
| miR-369-5p | 3 | 1 | IT |
| miR-370 | 3 | 1 | IT |
| miR-380 | 3 | 1 | IT |
| miR-384 | 3 | 1 | IT |
| miR-410 | 3 | 1 | IT |
| miR-411-5p | 3 | 1 | IT |
| miR-4485 | 3 | 1 | CC |
| miR-454 | 3 | 1 | CC |
| miR-485-3p | 3 | 1 | IT |
| miR-487b | 3 | 1 | IT |
| miR-545-5p | 3 | 1 | IT |
| miR-6243 | 3 | 1 | CC |
| miR-652-5p | 3 | 1 | CC |
| miR-660-5p | 3 | 1 | CC |
| miR-664-3p | 3 | 1 | IT |
| miR-668-3p | 3 | 1 | IT |
| miR-874-5p | 3 | 1 | IT |
| miR-885-3p | 3 | 1 | IT |
| miR-98 | 3 | 1 | IT |
| miR-LLT9 | 3 | 1 | CC |
