# Supplementary material for: The Role of Viral and Host MicroRNAs in the Aujeszky’s Disease Virus during the Infection Process
Source: PLoS One. 2014 Jan 24;9(1):e86965. doi: 10.1371/journal.pone.0086965 (PMC3901728; doi:10.1371/journal.pone.0086965)
Supplement: Table S6 — In silico target genes predicted for the most abundant DE porcine miRNAs in the infected groups. (DOCX) [file pone.0086965.s007.docx]

**Table S6. *In silico* target genes predicted for the most abundant DE porcine miRNAs in the infected groups.**

| **miRNA** | **Predicted target genes** |
| --- | --- |
| miR-92a | ABHD13, ACOX1, ACTC1, ADAM10, ADAM19, ADAM23, ADAMTS3, ADAMTSL1, ADAMTSL3, ADCY3, ADM, ADRB1, AFF1, AFF3, AFF4, AHCYL1, AKAP2, ALPK3, ALS2CR13, ALX4, ANGPTL2, ANKRD13C, ANKRD28, ANP32E, APOBEC3F, APOBEC3G, APOL6, APPL1, ARF1, ARFGEF1, ARHGEF10, ARHGEF17, ARID1B, ARID5B, ARMC1, ARPC2, ARRDC3, ARRDC4, ASB5, ASB7, ASPH, ASPN, ASXL2, ATP6V1B2, ATP8B1, ATRX, ATXN1, ATXN3, AURKA, AXL, AZIN1, B3GALT2, BAI3, BAZ2A, BAZ2B, BCAT2, BCL11A, BCL11B, BCL2L11, BCL9, BMPR2, BSDC1, BSN, BTBD12, BTBD14A, BTG2, C10orf118, C10orf22, C11orf56, C12orf5, C17orf39, C18orf1, C1orf21, C20orf111, C20orf39, C21orf66, C21orf91, C4orf16, C4orf40, C5orf24, C5orf28, C5orf30, C6orf103, C6orf62, C6orf98, CACNA1C, CACNA1I, CADM1, CALN1, CAMK2A, CAND1, CASD1, CASK, CASKIN1, CBFA2T3, CBLN4, CCDC113, CCDC131, CCNE2, CCNJ, CCNJL, CCT6A, CD2AP, CD69, CDC27, CDC42, CDC42BPA, CDCA7L, CDK5R1, CDK6, CDKN1C, CEP350, CFL2, CHD9, CHKA, CHMP7, CHRM2, CHRM5, CHST1, CHST7, CIC, CLCN5, CLDN11, CLGN, CLIP4, CLK3, CLTA, CNIH, CNNM4, CNOT2, CNTN4, COL12A1, COL19A1, COL1A2, COL27A1, COL5A1, COPS2, CPEB1, CPEB2, CPEB3, CPEB4, CPLX2, CPNE8, CPSF6, CRB1, CREB1, CREB3L2, CREM, CSMD3, CTNNBIP1, CTTNBP2, CUL3, CXADR, CXCL5, CXXC4, CXXC5, DAAM1, DAB2IP, DACT1, DAG1, DBT, DCLK2, DCP2, DCX, DDIT4, DDX3X, DDX3Y, DENND4B, DIAPH2, DIRAS1, DISC1, DKK3, DLGAP2, DMXL1, DNAJB12, DNAJB9, DOCK5, DOCK9, DPP10, DRGX, DSC2, DSCAML1, DST, DTX2, DUS2L, DUSP10, DUSP5, DUSP5P, DUSP6, DYNLT3, DYRK2, E2F3, EBAG9, EDEM1, EDEM3, EDG1, EDNRB, EGR2, EIF1, EIF2C4, EIF4G2, EIF5A2, ELOVL4, ELOVL6, EN2, EOMES, EPC2, EPHA8, EPS8, ERBB2IP, ERC2, ERGIC2, ESCO1, ESRRG, EVI5, EXOC5, EXOSC6, EZH2, FAM110B, FAM120A, FAM126B, FAM135A, FAM70A, FAM76B, FAM91A1, FARP1, FASLG, FBN1, FBN2, FBXL11, FBXO28, FBXO33, FBXW7, FCHO2, FHL2, FIGN, FKBP1C, FLI1, FMN2, FMR1, FNBP4, FNDC3B, FNIP1, FOXG1, FOXN2, FOXN3, FOXP2, FREM1, FRS2, FRY, FRYL, FRZB, FSTL1, FUNDC1, FXR1, FZD10, FZD6, G3BP2, GAN, GAP43, GATA2, GATA6, GATAD2B, GCLM, GDF11, GFPT2, GIT2, GLCE, GNPDA2, GNS, GOLGA1, GOLGA3, GOLGA4, GOLGA7, GOLGA8A, GOLGA8B, GOLGA8E, GOLGA8F, GOLGA8G, GPATCH8, GPBP1L1, GPM6A, GPR124, GPR137C, GPR158, GPR162, GPR180, GPR85, GRAMD3, GRHL1, GRIA1, GRIA4, GRID2, GRIP2, GRK5, GRM7, HAND1, HAND2, HAS3, HCN2, HDAC9, HECTD2, HECW1, HERC2, HERC3, HERC4, HERPUD2, HIPK1, HIPK3, HIVEP1, HMBOX1, HNF1B, HNRNPA0, HNRNPU, HNRPH1, HOXA9, HOXC8, HOXD10, IDH1, IKZF2, IKZF4, IL6ST, ING2, INSIG1, IQGAP2, IQWD1, IRS2, ITGA5, ITGA6, ITGAV, ITM2B, ITPR1, JARID2, JOSD1, KBTBD8, KCNA1, KCNC4, KCND2, KCNJ3, KCNK10, KIAA0240, KIAA0329, KIAA0831, KIAA1109, KIAA1128, KIAA1279, KIAA1370, KIAA1409, KIAA1432, KIAA1600, KIAA1632, KIAA1712, KIF3B, KIF5B, KLF12, KLF2, KLF4, KLF5, KLHL14, KLHL29, KLHL3, LATS2, LBX1, LCOR, LEPREL2, LHFPL2, LIMCH1, LIN28, LIN28B, LIN54, LMBR1L, LRP1B, LRRC1, LRRC4, LRRC8B, LUZP1, LYST, MACF1, MACROD2, MAN2A1, MAP2K4, MAP4K5, MAPK8, MARCH4, MARK1, MBOAT2, MCOLN2, MDN1, ME1, MEF2D, MEGF10, MFHAS1, MIA3, MIER3, MITF, MKL2, MLL5, MLLT10, MLSTD2, MMD, MMP16, MOAP1, MOBKL2A, MORC3, MPP1, MRPS25, MRS2L, MSRB3, MTDH, MTF1, MTHFD2, MTMR9, MTPN, MYCBP2, MYH9, MYLIP, MYLK, MYO18A, MYO1B, MYO5A, MYT1L, NARG1, NAT12, NAV3, NCOA1, NCOA3, NCOA6, NECAP1, NEDD4L, NEFH, NEFL, NEFM, NEGR1, NELF, NEUROD1, NF2, NFAT5, NFIA, NFIB, NFIX, NFYB, NFYC, NHLH2, NHSL1, NICN1, NIPBL, NKX2-4, NLK, NOL7, NOTCH1, NOV, NOVA1, NOX4, NPAL1, NPAS3, NPC1, NPTN, NR4A3, NRF1, NRK, NSF, NTRK2, NUP43, NUPL1, OAZ3, ODZ1, OTUD3, OTUD4, OXSR1, PAFAH1B1, PAIP1, PALLD, PALM2, PAPD5, PAPOLA, PAX3, PAX9, PCAF, PCDH10, PCDH11X, PCDH11Y, PCDH20, PCDH9, PCGF3, PCMTD1, PCOLCE2, PCTK1, PDE10A, PDE4D, PDS5A, PDS5B, PDSS2, PDXDC1, PDZD2, PER2, PFN2, PHF15, PHF17, PHF3, PHLPPL, PHTF2, PIAS4, PIK3AP1, PIK3R1, PIK3R3, PIP5K1C, PIP5K3, PITPNA, PITPNC1, PITPNM2, PLCB4, PLEKHA1, PLEKHA6, PLEKHB2, PLEKHG3, POLK, POLS, PPCS, PPP1R12A, PPP1R12C, PPP1R9A, PRDM13, PRDM16, PRKAB2, PRKAR2B, PRKCE, PROX1, PTAR1, PTEN, PTENP1, PTF1A, PTGER4, PTGFR, PTPRD, PTPRK, PTPRO, PVRL1, QKI, QSER1, RAB14, RAB23, RAB8B, RAD21, RAG1, RANBP9, RAP1A, RAP1B, RAP2C, RAPGEF6, RASAL2, RASSF2, RBJ, RBL2, RBM24, RBM35A, RBM9, RBPJ, REV3L, REXO1, RFX1, RFXDC2, RGS17, RGS3, RHPN2, RIMBP2, RIPK5, RIT1, RNF11, RNF141, RNF38, RNF4, RNF44, ROBO1, ROBO2, RPL15, RPS6KA4, RPS6KB1, RRBP1, RREB1, RSBN1, RUNDC2B, RYR3, SAR1B, SATB1, SATB2, SCN3A, SCUBE3, SDC2, SEC24A, SEC24C, SERTAD2, SERTAD3, SESN3, SETD5, SETD7, SFMBT2, SFRS2IP, SGK, SGK3, SGPP1, SH3PXD2A, SHOX, SIM1, SIM2, SLC12A2, SLC12A5, SLC17A6, SLC1A2, SLC24A3, SLC25A32, SLC2A14, SLC2A3, SLC38A2, SLC44A1, SLC4A7, SLC5A7, SLC6A1, SLC7A11, SLC9A1, SLC9A2, SMAD6, SMAD7, SMURF1, SNAP91, SNAPC1, SNF1LK, SNN, SOBP, SOCS5, SOCS6, SORCS3, SORL1, SOX11, SOX4, SP4, SPHK2, SPN, SPOCK2, SPRYD4, SRPK2, SRPR, SSBP3, SSFA2, STAG2, STAT2, STEAP2, STK39, STRN, STRN3, STYX, SUDS3, SUPV3L1, SUV420H1, SYNE1, SYNJ1, SYT1, TACC2, TAGAP, TBC1D12, TBX3, TCFL5, TEAD1, TEF, TEX2, TFAP2A, TGIF1, TMCC1, TMCC3, TMEM119, TMEM16C, TMEM16H, TMEM184B, TMEM87A, TMEPAI, TMF1, TNPO1, TNRC6B, TOB2, TOP1, TP63, TPCN1, TRAF3, TRAK2, TRAM2, TRIM33, TRIM34, TRIM36, TRIM6, TRIO, TSC1, TSGA14, TTC28, TTC9, TULP4, TWF1, TWIST1, UBE2G1, UBE2W, UBE2Z, UBR1, UGP2, UNC84A, UPF2, USF2, USP28, USP44, VASH2, VAX1, VEZF1, VPS13B, VPS54, WASL, WDFY3, WDR32, WDR42A, WDR81, WNT5A, WWC1, WWP2, XRN1, XYLT2, YIPF4, YTHDF3, YWHAZ, ZBTB34, ZBTB40, ZBTB8, ZCCHC2, ZDHHC21, ZDHHC3, ZDHHC5, ZEB2, ZFHX3, ZFPM2, ZNF238, ZNF287, ZNF295, ZNF512B, ZNF521, ZNF532, ZNF533, ZNF626, ZNF654, ZNF659, ZNF711, ZNF804A. |
| miR-92b-3p | ABCF2, ABHD13, ABI3BP, ACOX1, ACTC1, ADAM10, ADAM19, ADAM23, ADAMTS3, ADAMTS9, ADAMTSL1, ADAMTSL3, ADCY3, ADM, ADRB1, AFF1, AFF3, AFF4, AGGF1, AHCYL1, AKAP2, ALPK3, ALS2CR13, ALX4, ANGPTL2, ANKRD13C, ANKRD28, ANP32E, APOBEC3F, APOBEC3G, APOL6, APPL1, ARF1, ARFGEF1, ARHGEF10, ARHGEF17, ARID1B, ARID5B, ARMC1, ARPC2, ARRDC3, ARRDC4, ASB5, ASB7, ASPH, ASPN, ASXL2, ATP6V1B2, ATP8B1, ATRX, ATXN1, ATXN3, ATXN3L, AURKA, AXL, AZIN1, B3GALT2, BACH1, BAI3, BAZ2A, BAZ2B, BCAT2, BCL11A, BCL11B, BCL2L11, BCL9, BICC1, BMPR2, BRMS1L, BSDC1, BSN, BTBD12, BTBD14A, BTG2, C10orf118, C10orf22, C11orf24, C11orf56, C12orf5, C14orf28, C17orf39, C18orf1, C18orf25, C1orf21, C1orf80, C20orf111, C20orf39, C21orf66, C21orf91, C4orf16, C4orf40, C5orf24, C5orf28, C5orf30, C5orf5, C6orf103, C6orf107, C6orf62, C6orf98, CACNA1C, CACNA1H, CACNA1I, CADM1, CALM1, CALM2, CALM3, CALN1, CAMK2A, CAMTA1, CAND1, CASD1, CASK, CASKIN1, CBFA2T3, CBLN4, CCDC113, CCDC131, CCNE2, CCNJ, CCNJL, CCT6A, CD2AP, CD69, CDC27, CDC42, CDC42BPA, CDCA7L, CDH10, CDK5R1, CDK6, CDKN1C, CEP350, CFL2, CHD9, CHGA, CHKA, CHMP7, CHRM2, CHRM5, CHST1, CHST7, CIC, CLCN5, CLDN11, CLEC16A, CLGN, CLIP4, CLK3, CLTA, CNIH, CNNM4, CNOT2, CNR1, CNTN4, COL11A1, COL12A1, COL19A1, COL1A2, COL27A1, COL5A1, COPS2, CPEB1, CPEB2, CPEB3, CPEB4, CPLX2, CPNE8, CPSF6, CRB1, CREB1, CREB3L2, CREM, CSMD3, CTNNBIP1, CTTNBP2, CUL3, CXADR, CXCL5, CXXC4, CXXC5, DAAM1, DAB2IP, DACT1, DAG1, DBT, DCLK2, DCP2, DCX, DDIT4, DDX3X, DDX3Y, DENND4B, DIAPH2, DIRAS1, DISC1, DKK3, DLGAP2, DMXL1, DNAJB12, DNAJB9, DNMT3A, DOCK5, DOCK9, DPP10, DRGX, DSC2, DSCAML1, DST, DTX2, DUS2L, DUSP1, DUSP10, DUSP5, DUSP5P, DUSP6, DYNLT3, DYRK2, E2F3, EBAG9, EDEM1, EDEM3, EDG1, EDNRB, EGR2, EIF1, EIF2C4, EIF4G2, ELOVL4, ELOVL6, EN2, EOMES, EPC2, EPHA8, EPS8, ERBB2IP, ERC2, ERGIC2, ESCO1, ESRRG, EVI5, EXOC5, EXOSC6, EZH2, FAM110B, FAM120A, FAM126B, FAM135A, FAM46A, FAM70A, FAM76B, FAM91A1, FARP1, FASLG, FBN1, FBN2, FBXL11, FBXO28, FBXO33, FBXW7, FCHO2, FHL2, FHL3, FIGN, FKBP1C, FLI1, FMN2, FMR1, FNBP4, FNDC3B, FNIP1, FOXG1, FOXN2, FOXN3, FOXP2, FREM1, FRS2, FRY, FRYL, FRZB, FSTL1, FUNDC1, FXR1, FZD10, FZD6, G3BP2, GAN, GAP43, GATA2, GATA6, GATAD2B, GCLM, GDF11, GFPT2, GIT2, GLCE, GNPDA2, GNS, GOLGA1, GOLGA3, GOLGA4, GOLGA7, GOLGA8A, GOLGA8B, GOLGA8E, GOLGA8F, GOLGA8G, GPATCH8, GPBP1L1, GPM6A, GPR124, GPR137C, GPR158, GPR162, GPR180, GPR85, GRAMD3, GRHL1, GRIA1, GRIA4, GRID2, GRIP2, GRK5, GRM7, HAND1, HAND2, HAS3, HCN2, HDAC9, HECTD2, HECW1, HERC2, HERC3, HERC4, HERPUD2, HHIP, HIPK1, HIPK3, HIVEP1, HMBOX1, HNF1B, HNRNPA0, HNRNPA1, HNRNPU, HNRPH1, HOXA9, HOXB8, HOXC8, HOXD10, ICK, IDH1, IKZF2, IKZF4, IL6ST, ING2, INSIG1, INSL5, IQGAP2, IQWD1, IRS2, ITGA5, ITGA6, ITGAV, ITM2B, ITPR1, JAKMIP1, JARID2, JAZF1, JOSD1, JUN, KBTBD8, KCNA1, KCNC4, KCND2, KCNJ3, KCNK10, KIAA0240, KIAA0329, KIAA0831, KIAA1109, KIAA1128, KIAA1279, KIAA1370, KIAA1409, KIAA1432, KIAA1600, KIAA1632, KIAA1712, KIF1B, KIF3B, KIF5B, KLF12, KLF2, KLF4, KLF5, KLHL14, KLHL29, KLHL3, KPNA5, KRT1, LATS2, LBX1, LCOR, LEPREL2, LHFPL2, LIN28, LIN28B, LIN54, LMBR1L, LMO2, LRP1B, LRRC1, LRRC20, LRRC4, LRRC8B, LUZP1, LYST, MACF1, MACROD2, MAN2A1, MAP2K4, MAP4K5, MAPK8, MARCH4, MARK1, MBOAT2, MCOLN2, MDN1, ME1, MEF2D, MEGF10, MFHAS1, MIA3, MIER3, MITF, MKL2, MLL5, MLLT10, MLSTD2, MMD, MMP16, MOBKL2A, MORC3, MPP1, MRPS25, MRS2L, MSRB3, MTDH, MTF1, MTHFD2, MTMR10, MTMR9, MTPN, MYCBP2, MYH3, MYH9, MYLIP, MYLK, MYO18A, MYO1B, MYO5A, MYT1L, NARG1, NAT12, NAT13, NAV3, NCK2, NCOA1, NCOA3, NCOA6, NECAP1, NEDD4L, NEFH, NEFL, NEFM, NEGR1, NELF, NEUROD1, NF2, NFAT5, NFIA, NFIB, NFIX, NFYB, NFYC, NHLH2, NHSL1, NICN1, NIPBL, NKX2-4, NLK, NOL7, NOTCH1, NOV, NOVA1, NOX4, NPAL1, NPAS3, NPC1, NPNT, NPTN, NR4A3, NRF1, NRIP1, NRK, NSF, NSMAF, NTRK2, NUP43, NUPL1, NUTF2, OAZ3, ODZ1, OTUD3, OTUD4, OXSR1, PAFAH1B1, PAIP1, PALLD, PALM2, PANK3, PAPD5, PAPOLA, PAX3, PAX9, PCAF, PCDH10, PCDH11X, PCDH11Y, PCDH20, PCDH9, PCGF3, PCMTD1, PCOLCE2, PCTK1, PDCD6IP, PDE10A, PDE4D, PDE8A, PDS5A, PDS5B, PDSS2, PDXDC1, PDZD2, PER2, PFN2, PHF15, PHF17, PHF3, PHLPPL, PHTF2, PIAS4, PIK3AP1, PIK3R1, PIK3R3, PIP4K2C, PIP5K1C, PIP5K3, PITPNA, PITPNC1, PITPNM2, PLCB4, PLEKHA1, PLEKHA6, PLEKHB2, PLEKHG3, PLEKHM1, POLK, POLS, PPCS, PPP1R12A, PPP1R12C, PPP1R9A, PRDM13, PRDM16, PRKAB2, PRKAR2B, PRKCE, PROX1, PSMD14, PTAR1, PTEN, PTENP1, PTF1A, PTGER4, PTGFR, PTPRD, PTPRK, PTPRO, PVRL1, QKI, QSER1, RAB14, RAB23, RAB7A, RAB8B, RAD21, RAG1, RANBP9, RAP1A, RAP1B, RAP2C, RAPGEF6, RASAL2, RASSF2, RBJ, RBL2, RBM24, RBM35A, RBM9, RBPJ, RECK, REV3L, REXO1, RFX1, RFXDC2, RGL1, RGS17, RGS3, RHPN2, RIMBP2, RIPK5, RIT1, RNF11, RNF141, RNF38, RNF4, RNF44, ROBO1, ROBO2, RPL15, RPS6KA4, RPS6KB1, RRBP1, RREB1, RSBN1, RUNDC2B, RYR3, SAR1B, SATB1, SATB2, SCN3A, SCUBE3, SDC2, SEC24A, SEC24C, SEMA3A, SERTAD2, SERTAD3, SESN3, SETD5, SETD7, SETDB1, SFMBT2, SFRS2IP, SGK, SGK3, SGMS2, SGPP1, SH3PXD2A, SHOX, SIM1, SIM2, SIRPA, SLC12A2, SLC12A5, SLC17A6, SLC1A2, SLC24A3, SLC25A32, SLC2A14, SLC2A3, SLC32A1, SLC38A2, SLC44A1, SLC5A7, SLC6A1, SLC7A11, SLC9A1, SLC9A2, SLC9A3R2, SLITRK5, SMAD6, SMAD7, SMURF1, SNAP91, SNAPC1, SNF1LK, SNN, SNX2, SOBP, SOCS5, SOCS6, SORCS3, SORL1, SOSTDC1, SOX11, SOX4, SP4, SPEN, SPHK2, SPN, SPOCK2, SPRYD4, SPTBN4, SRPK2, SRPR, SSBP3, SSFA2, STAG2, STAT2, STK39, STRN, STRN3, STYX, SUDS3, SUHW4, SUPV3L1, SUV420H1, SYNE1, SYNJ1, SYT1, TACC2, TAF15, TAGAP, TBC1D12, TBX3, TCFL5, TEAD1, TEF, TEX2, TFAP2A, TGIF1, TMCC1, TMCC3, TMEM16C, TMEM16D, TMEM16H, TMEM184B, TMEM87A, TMEPAI, TMF1, TNPO1, TNRC6B, TOB2, TOP1, TP63, TPCN1, TRAF3, TRAK2, TRAM2, TRIM33, TRIM34, TRIM36, TRIM6, TRIO, TSC1, TSGA14, TTC28, TTC9, TULP4, TWF1, TWIST1, UBE2G1, UBE2W, UBE2Z, UBR1, UGP2, UNC84A, UPF2, USF2, USP28, USP44, VAMP2, VASH2, VAX1, VCL, VEZF1, VPS13B, VPS4B, VPS54, WASL, WDFY3, WDR32, WDR42A, WDR81, WNT5A, WWC1, WWP2, XRN1, XYLT2, YIPF4, YTHDF3, YWHAZ, ZBTB34, ZBTB40, ZBTB46, ZBTB8, ZCCHC2, ZDHHC21, ZDHHC3, ZDHHC5, ZEB2, ZFHX3, ZFPM2, ZNF238, ZNF287, ZNF295, ZNF512B, ZNF521, ZNF532, ZNF533, ZNF626, ZNF654, ZNF659, ZNF711, ZNF804A. |
| miR-133a | ACVR2B, ADCY1, ADIPOQ, ANGPTL4, AZIN1, BAZ2A, BCL2L2, BCORL2, BICC1, BRUNOL4, BTBD14A, BTN2A3, C10orf38, C20orf117, CAPN5, CCDC88B, CCDC88C, CDC2L5, CDH23, CKAP5, CLTA, CMPK, CORO1C, CREB5, DDX3X, DLGAP3, DNM2, DOK1, ELAVL1, EPHA7, FAM46A, FBXL19, FGFR1, FOSL2, FOXP2, GDNF, GGTLA1, GLI3, GPM6A, GPR162, GSN, IGF1R, IHPK1, ITGA3, KCND3, KCNIP2, KIAA0323, KIAA0652, KIAA1147, KIAA1602, KIAA1853, KIF1A, KIF3C, KPNA6, KREMEN1, LASP1, LASS2, LEPREL2, LHFP, LIMD2, MDN1, MGAT3, MLL, MLL5, MMP24, NADK, NAT11, NCOA6, NLGN3, NOS1, NUP153, PAN3, PCDHGA11, PCDHGA12, PCDHGA2, PCDHGA3, PCDHGA6, PCDHGA8, PCDHGB7, PCDHGC3, PCDHGC4, PCDHGC5, PER1, PER2, PITPNM2, POU2F1, PPP1R12C, PPP1R9B, PPP2CA, PPP2R5D, PRRT2, PTBP1, PTBP2, PTPRD, PTPRK, QKI, RAG1, RIMS4, RNF44, ROD1, SCN2B, SEC14L5, SFXN5, SLC2A12, SLC46A1, SLC6A1, SLC7A8, SMARCD1, SMEK1, SMEK2, SMPD3, SNRK, SNX30, SOBP, SOLH, SREBF2, SRGAP2, STAU1, SVOP, SYDE1, SYT2, TAGLN2, TMC6, TNR, TRAM2, TRO, TTBK1, TTYH3, TUSC5, TXLNA, TXNDC4, UBR4, WIPI2, YPEL2, ZBTB7B, ZHX3, ZNF436. |
| miR-133b | ABCC1, ACAT2, ACTR1A, ADAMTS19, ADAMTS5, ADCY5, ADCY6, ADCYAP1, AFAP1, AFTPH, AKAP9, ALCAM, ALDH5A1, ALPI, ALS2CR13, AMMECR1L, ANK2, ANK3, ANKRD12, ANKRD28, AP1S2, AP2M1, APPL2, ARFIP2, ARHGAP12, ARHGAP24, ARHGDIA, ARL3, ARPC1A, ARPC5, ASH1L, ATP2B3, ATP6AP2, ATP7B, ATRX, AUH, B3GALNT1, B3GAT2, B3GNT2, BACE1, BAT2D1, BAZ2A, BCL11A, BCL2L1, BCL2L2, BCORL1, BICC1, BNC2, BNIP3L, BPTF, BRUNOL4, BRUNOL6, BTBD10, BTBD3, BTN2A3, C10orf39, C10orf46, C11orf58, C11orf9, C14orf101, C14orf45, C16orf5, C1orf34, C20orf11, C20orf117, C20orf121, C5orf25, C5orf4, C5orf5, C6orf204, C9orf58, CACNA1B, CAMKK2, CAP1, CAPN5, CAV2, CBFA2T2, CBLN2, CCDC117, CD2AP, CD300LB, CD47, CDC2L5, CDC42BPA, CDK5R1, CECR6, CETN3, CKAP4, CLASP2, CLCN5, CLCN6, CLTA, CMPK, CNKSR3, CNNM2, CNOT1, CNOT6L, COL19A1, COL1A1, COL5A3, COL6A3, CORO1C, CPEB2, CPNE3, CREB5, CRK, CSNK1G1, CSNK1G3, CSPG4, CTBP2, CTGF, CUGBP2, CUL4A, CUL4B, CXXC4, DAB1, DCUN1D4, DDX3X, DDX3Y, DIAPH2, DLG2, DLGAP2, DLGAP3, DMXL1, DMXL2, DNAJB1, DNASE1L1, DOLPP1, DST, DUSP1, DYNC1LI2, DYNLT3, EDEM1, EEF1A1, EEF1AL3, EGFR, EIF2C1, EIF3J, EIF4A1, EIF4G3, ELAVL1, ELF2, ELOVL4, ENAH, ENC1, ENPEP, ENPP3, EPB41, EPB41L2, EPB41L3, EPHA4, EPHA7, EPHB4, ESRRG, ETF1, EVI1, EXDL2, EYA1, EYA4, FAIM, FAM117A, FAM46A, FAM46C, FAM49A, FAM57A, FAM62B, FBN1, FBN2, FBXL11, FBXL19, FBXL2, FBXO3, FBXW11, FGF1, FGFR1, FLI1, FOXC1, FOXG1, FOXK2, FOXN3, FOXP1, FOXP2, FOXP4, FOXQ1, FRS2, FTL, FURIN, GABARAPL1, GABARAPL3, GABRB2, GARNL3, GBA2, GCH1, GDNF, GLRA2, GLS, GLS2, GMEB1, GPM6A, GPR173, GRIA2, GRID2, GRM7, GZF1, HECW1, HIC2, HIVEP2, HLF, HLTF, HORMAD2, HOXA9, HS2ST1, ID4, IDH1, IGF1R, IGF2BP2, IKZF2, IKZF4, INSR, IRS2, JAZF1, JHDM1D, JPH1, KCNA1, KCND3, KCNMA1, KCTD20, KIAA0232, KIAA0323, KIAA0430, KIAA1045, KIAA1219, KIAA1239, KIAA1429, KIAA1432, KIAA1522, KIAA1600, KIAA2022, KIF13A, KIF21A, KIF3C, KIRREL, KLF5, KLHDC5, KLHL32, KLRC4, KLRK1, KPNA6, LANCL2, LASP1, LASS2, LCORL, LDLRAP1, LHFP, LHFPL4, LHX5, LIN28B, LIN7C, LPGAT1, LPIN2, LRRC7, LRRFIP1, LTBP1, LZTS2, MACF1, MAEA, MAFG, MAN1A2, MAP2K4, MAP3K3, MAPK14, MARCH9, MBNL1, MCL1, MDN1, MECP2, MED12L, MEIS1, MEIS2, MGA, MIER3, MITF, MKL2, MLL, MLL3, MLL5, MLLT3, MLLT4, MLXIP, MMP15, MRC2, MSN, MTF1, MTMR4, MTPN, MYH9, MYO9A, NAGS, NAT11, NCALD, NCAM2, NCOA6, NDRG1, NEBL, NFAT5, NFATC2, NFIB, NHSL1, NIPA1, NKX2-2, NLGN3, NME4, NMT1, NOS1, NPTX1, NR2E1, NR5A2, NRIP3, NRK, NTRK3, NUMB, NUP153, NUP160, NUS1, OGDHL, ONECUT2, OTUD3, OTUD4, OTX2, PAK7, PAN3, PAPD5, PAPPA, PCDH1, PCDH17, PCDHGA11, PCDHGA12, PCDHGA2, PCDHGA3, PCDHGA6, PCDHGA8, PCDHGB7, PCDHGC3, PCDHGC4, PCDHGC5, PCGF2, PCGF6, PCNP, PDE10A, PDIK1L, PER1, PERQ1, PEX5L, PFKFB3, PFN2, PHTF2, PI15, PIK3R3, PIP4K2B, PIP5K3, PITPNM2, PLCB4, PLEKHA3, PML, PMP22, POLH, POU2F1, POU2F2, POU3F2, POU4F1, PPFIA3, PPP2CA, PPP2CB, PPP2R2D, PPP2R5D, PPP2R5E, PRDM1, PRDM16, PRDM6, PRRT2, PRRX1, PSD3, PSEN1, PTBP1, PTBP2, PTGFRN, PTPN22, PTPRD, PTPRK, PTPRO, PTPRZ1, PXDN, QKI, RAB30, RAB34, RAB5C, RABGAP1, RABGAP1L, RAG1AP1, RANBP5, RAP2C, RAPH1, RARB, RAVER1, RB1CC1, RBMS1, RBPJ, RC3H1, RCE1, RCOR1, RFFL, RFXDC2, RHOQ, RIMS4, RIPK5, RNF38, RNF44, ROD1, RPE, RRP15, SACM1L, SAE2, SAMD5, SAPS3, SCAF1, SCN2B, SCOC, SDCCAG3, SDK2, SEC61B, SEMA5B, SENP1, SERBP1, SESN1, SF3B4, SFMBT2, SFXN5, SGCD, SGK, SGMS2, SGPP1, SGTB, SH3GL2, SH3PXD2B, SIRT1, SIX5, SLC25A36, SLC30A5, SLC30A7, SLC39A1, SLC44A1, SLC6A1, SLC6A6, SLITRK5, SLTM, SMARCA2, SMARCD1, SMPD3, SNORA67, SNRK, SNX30, SOBP, SOLH, SOX4, SP1, SP3, SPTBN1, SPTLC2, SPTY2D1, SRGAP2, SRGAP3, ST8SIA3, STARD13, STAU1, STC1, STOM, STX5, STX6, STXBP5, STXBP6, SUMO1, SUMO1P3, SUPT7L, SV2A, SYAP1, SYT1, SYT2, SYT9, TAF4B, TAGLN2, TBL1X, TBPL1, TEAD1, TFAP2B, TFAP2D, TFE3, TFG, TGFBR1, TGOLN2, TIMM17A, TM9SF3, TMEM178, TMEM29, TMEM29B, TMEM32, TMEM57, TNFRSF10B, TNKS, TNPO1, TNR, TNRC6B, TPD52, TRAM2, TRERF1, TRHDE, TRIM2, TRIM33, TRIM55, TRO, TSPAN18, TTN, TTYH3, TULP4, TXLNA, UBE2Q1, UBE2Z, UBFD1, UBR4, UBXD7, UBXD8, UNC13A, USP32, USP6, VAMP3, VAPB, VAT1, VKORC1, VPS13B, VPS54, WIPF2, WIPI2, WNT5A, XPNPEP1, XPO1, XPO4, XYLT1, YES1, YPEL2, YTHDF3, ZBTB34, ZBTB7B, ZBTB8, ZC3H11A, ZC3H14, ZFHX3, ZFP161, ZHX1, ZHX3, ZIC3, ZNF131, ZNF217, ZNF263, ZNF385, ZNF395, ZNF436, ZNF654, ZNF710, ZNF740, ZNRF3, ZSWIM6. |
| miR-378 | ADAR, BANP, C1orf132, C20orf91, C9orf5, CHRNA4, CRLS1, E2F2, EIF2C1, FANCA, FBXO40, FMNL3, GNA13, IPO9, KCNAB2, KLK4, MLL, OTUB2, PI4K2A, PITPNA, POU2F1, PTPRT, SLC38A1, TMCC3, UHRF1, VANGL1, ZNF134, ZNF776. |
| miR-206 | ABCB7, ABHD2, ADAM12, ADAR, ADCY1, ADPGK, AKAP11, AKAP2, PALM2, AMOT, ANKRD29, ANKRD38, ANXA2, ANXA2P3, ANXA4, AP1G1, API5, ARCN1, ARF3, ARFIP1, ARHGEF18, ARL10, ARSD, ASAH3L, ASH2L, ASXL2, ATF2, ATXN7, AZIN1, BACH2, BAG4, BAIAP2, BCL11A, BCL11B, BCL7A, BDNF, BICD1, BSCL2, BSN, BZRAP1, C10orf26, C10orf68, CCDC7, C11orf61, C17orf62, RNF165, C18orf23, C1orf96, C20orf112, C4orf34, C6orf120, C6orf89, C7orf23, C9orf82, CAND1, CAPRIN1, CBL, CCDC141, CCND2, CD164, CD28, CD2AP, CDC42, CDC42SE1, CDK6, CDON, CEBPZ, CENTB2, CLCN3, CLTC, CNN3, CNTNAP2, COL19A1, COL4A3, COL4A3BP, CORO1C, CPEB1, CPLX2, CREB5, CREBL2, CSNK1G1, CTBP2, CTTNBP2NL, CXorf23, CYP2U1, DCP2, DDX5, DENND2C, DGKH, DICER1, DLG4, DMRT2, E2F5, EDN1, EFNB2, EIF2C1, EIF4E, EIF4G3, ELOF1, ELOVL6, EPB41L1, ERC1, ETS1, FAM107B, FAM46C, FAM63B, FAM91A1, FBXO33, FN1, FNBP1L, FNDC3A, FNDC3B, FOSB, FOXP1, FRAS1, FRMD4A, FRS2, FUBP1, G3BP1, G6PD, GAN, GARNL1, GAS2L1, GCH1, GIT1, GJA1, GK5, GLCCI1, GLIS2, GNE, GNPDA2, GOLPH3, GPD2, GPR137C, GPR158, GPR6, GPR85, H3F3A, H3F3B, HACE1, HDAC4, HEYL, HIAT1, HIC2, HIVEP1, HIVEP3, HMBOX1, HMG2L1, HMGCR, HMGN1, HNRNPU, HNRPA3, HOOK1, HOXB4, HS3ST3B1, HSPD1, IGF1, IPO9, ITGB1BP1, JARID2, JOSD1, KCND3, KCNIP3, KCNJ2, KIAA0323, KIAA0329, KIAA0652, KIAA1045, KIAA1462, KIAA2022, KIF2A, KLF13, KLHDC5, KRAS, KTN1, LASP1, LRAT, LRRC8A, LYRM5, MAB21L1, MAL2, MAN1C1, MAP1A, MAP3K1, MAP4K3, MAPK1, MAPKBP1, MATR3, MDN1, MED1, MEIS1, MEOX2, MET, MEX3C, MGAT4A, MIER1, MIPOL1, MLL, MLL5, MLLT3, MLLT4, MMD, MMD2, MNT, MOBKL2C, MON2, MPP5, MPP7, MRAS, MXD1, MYLK, MYO15A, NANP, NBEA, NCBP1, NCL, NCOA1, NDRG3, HNRPA3P2, NFAT5, NPAS3, NR3C1, NRP1, NXT2, OAT, OLFML2A, ONECUT2, OSBPL7, OTX2, PAFAH1B1, PAQR5, PAX3, PBEF1, PCDH17, PDCD4, PDGFA, PDIK1L, PFN2, PFTK1, PGD, PHLPPL, PLEKHA7, PLEKHQ1, POGK, PPIB, PPP4R2, PRKACB, PRKCE, PRKRIR, PTBP1, PTPLAD1, PTPN1, PTPRC, PTPRG, PTPRK, PTPRT, PVRL3, QKI, RAB43, ISY1, RAB5A, RABEPK, RABGAP1, RASA1, RGS7, RIMS4, RIT2, RNF138, RNF145, RNF44, RSBN1, S100A7A, SDPR, SEC63, SEMA6D, SETBP1, SFRP1, SFRS1, SFRS10, SFRS2IP, SFRS9, SHANK2, SLC10A7, SLC1A2, SLC25A16, SLC25A22, SLC25A25, SLC25A30, SLC29A3, SLC2A13, SLC31A1, SLC35B4, SLC35F1, SLC38A3, SLC39A10, SLC44A1, SLC6A15, SLC7A11, SLC7A2, SLC8A1, SLC8A2, SMAD4, SMARCB1, SMARCC1, SMEK2, SMG7, SNAP25, SNX2, SOS1, SOX5, SOX6, SOX9, SP1, SP2, SPEG, SPHK2, SPRED1, SPTLC3, SRGAP2, STARD7, STC2, STX12, STXBP4, SUHW4, SULF1, SYNJ2, SYT1, TACR1, TAGLN2, TCF7L2, TEX2, TGIF2, THBS1, TIMP3, TLE4, TLOC1, TMCC1, TMEM178, TMEM55B, TMSB4X, TMSL1, TMSL2, TMSL6, TMSL3, TMSL4, TNKS2, TNPO1, TNPO2, TNRC6B, TNS3, TPM3, TPPP, TRAPPC3, TRHDE, TRIM2, TSPYL4, TWF1, UBE2H, UBE4A, UBN1, UBQLN1, UBXD3, UNC50, UST, UTRN, VAMP2, VAMP4, VGLL4, WDR1, WDR48, WEE1, WIPF2, WNK3, WNT3, XPO6, YLPM1, YWHAQ, YWHAZ, ZBTB4, ZC3H7B, ZFP36L2, ZMAT3, ZNF236, ZNF571. |
