# Supplementary material for: The Role of Viral and Host MicroRNAs in the Aujeszky’s Disease Virus during the Infection Process
Source: PLoS One. 2014 Jan 24;9(1):e86965. doi: 10.1371/journal.pone.0086965 (PMC3901728; doi:10.1371/journal.pone.0086965)

viR02 - In vitro
Begonia and NIA-3 groups

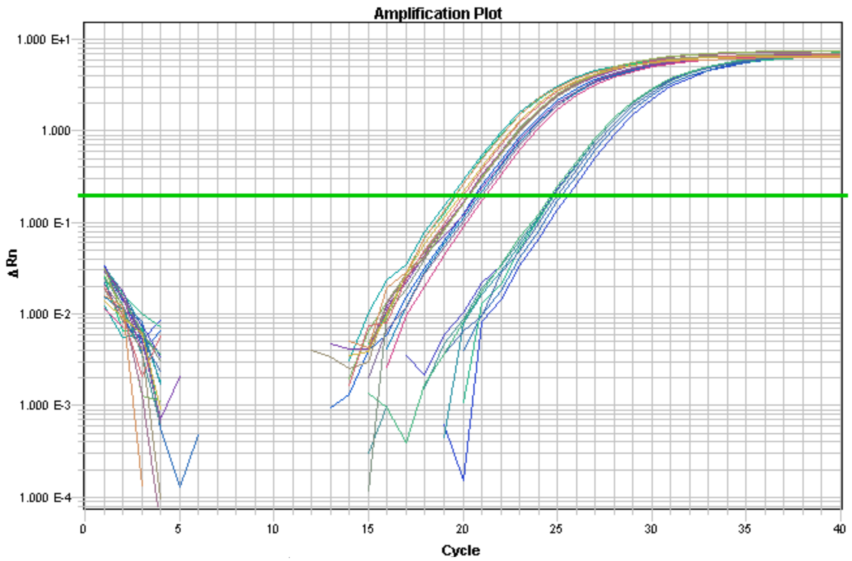

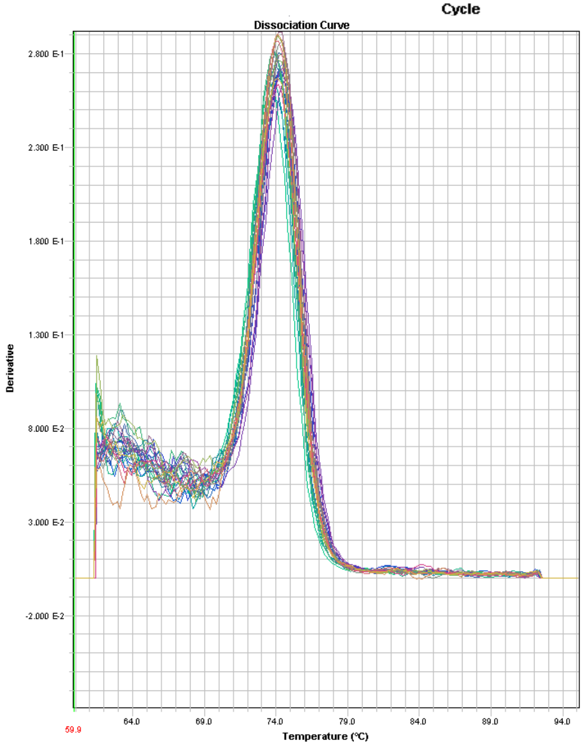

viR02 - In vitro
Mock-infected group

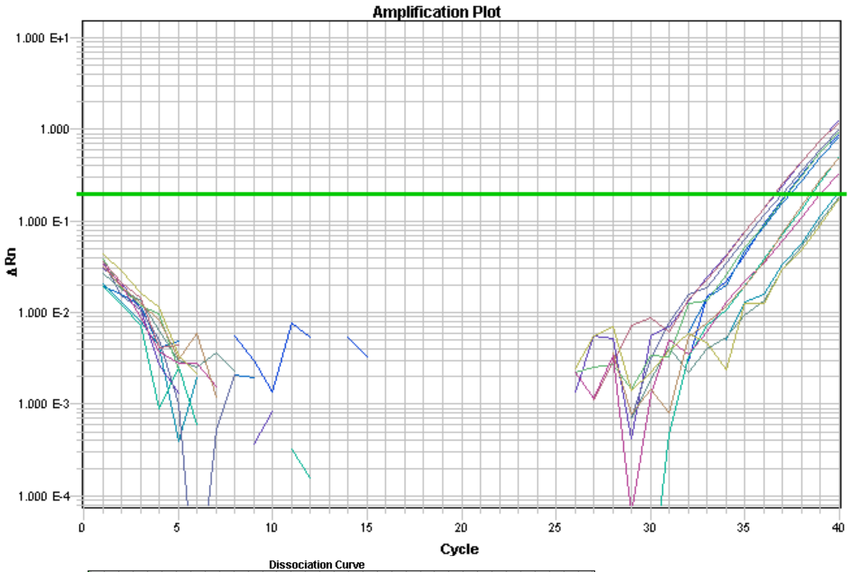

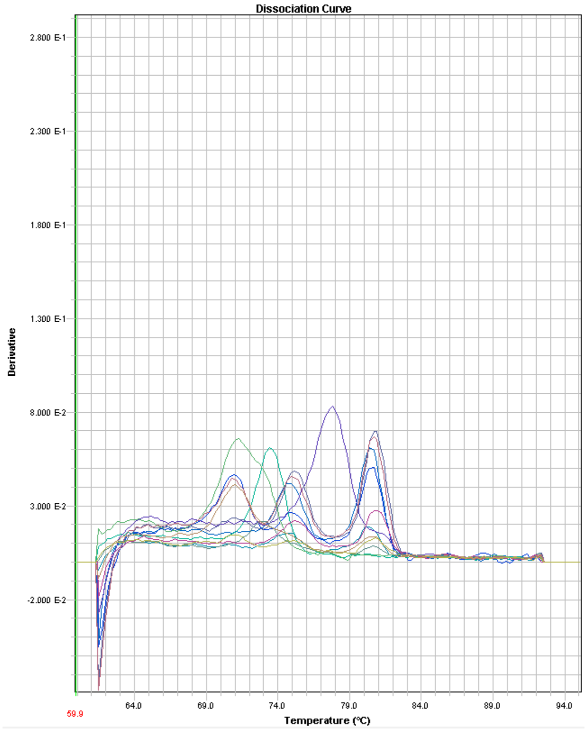

viR04 - In vitro
Begonia and NIA-3 groups

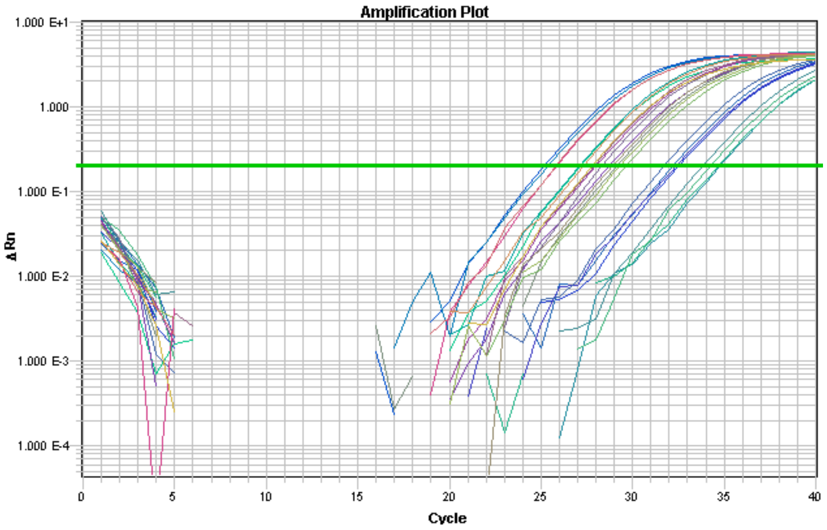

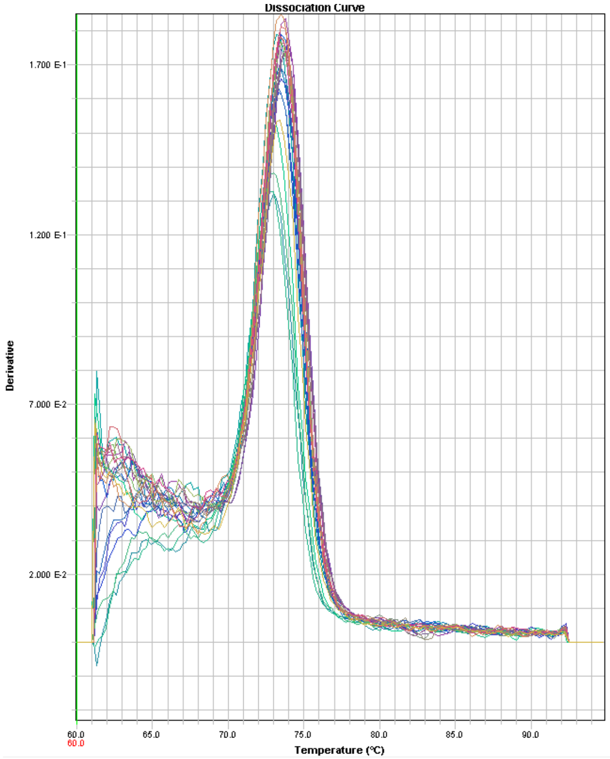

viR04 - In vitro
Mock-infected group

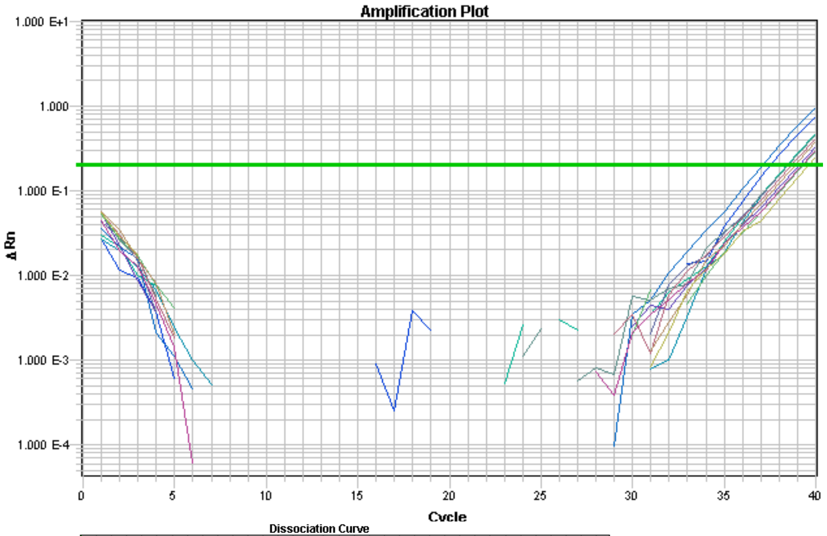

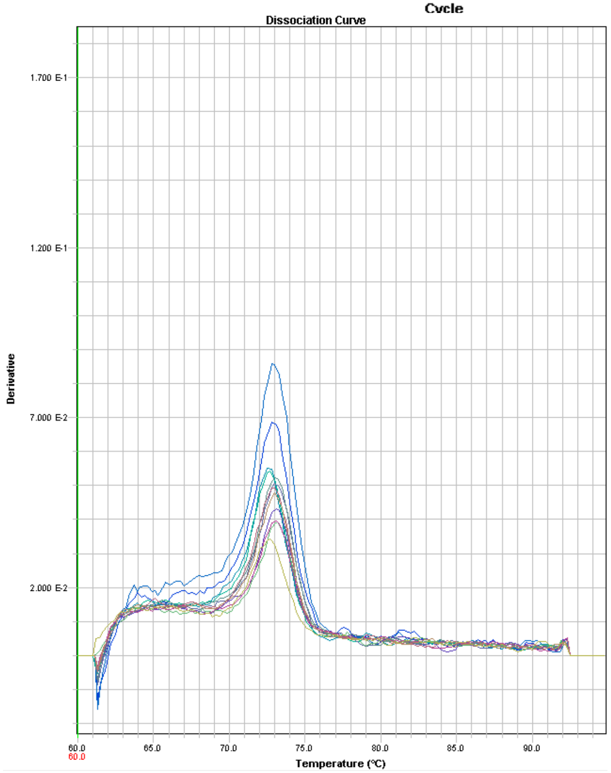

viR06 - In vitro
Begonia and NIA-3 groups

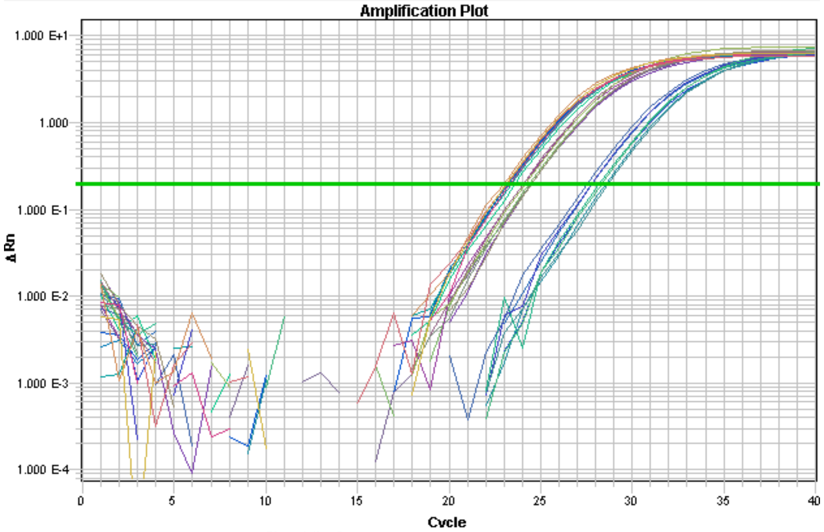

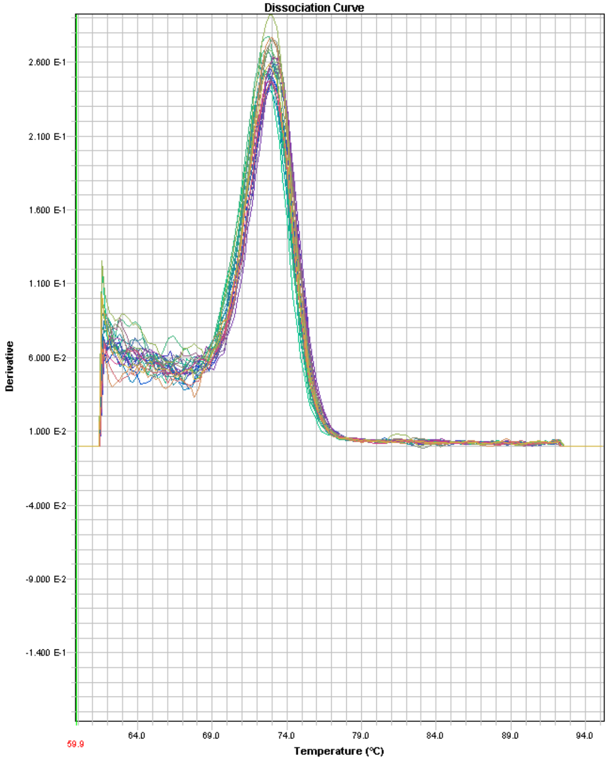

viR06 - In vitro
Mock-infected group

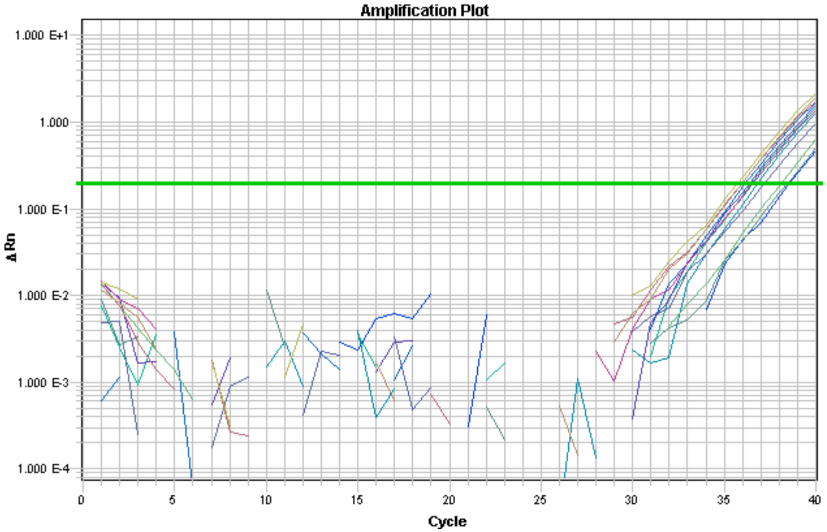

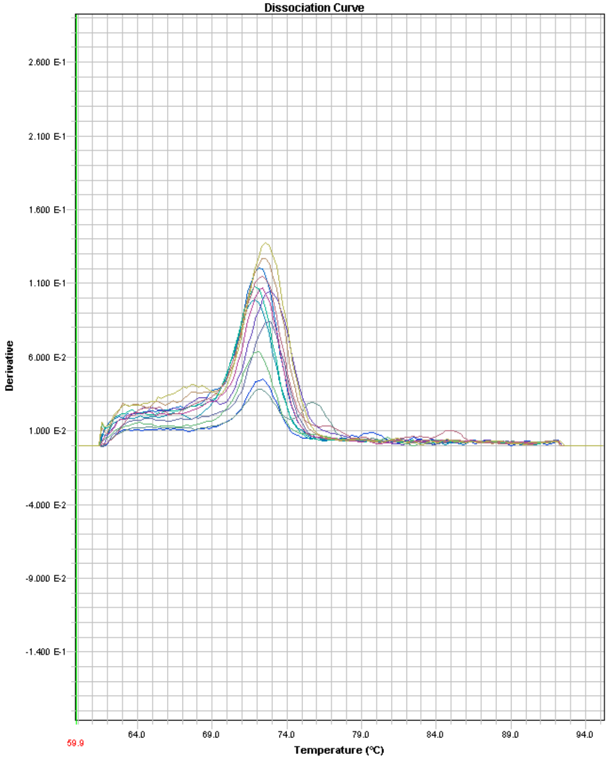

viR09 - In vitro
Begonia and NIA-3 groups

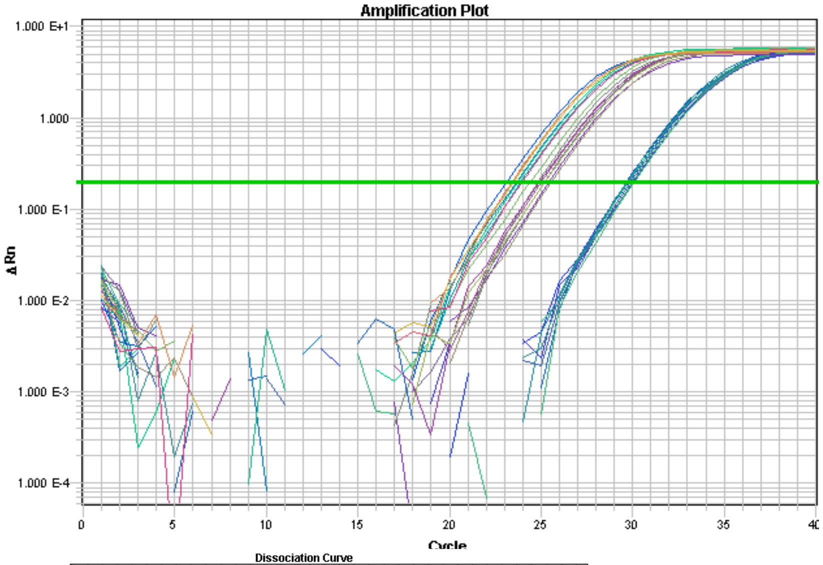

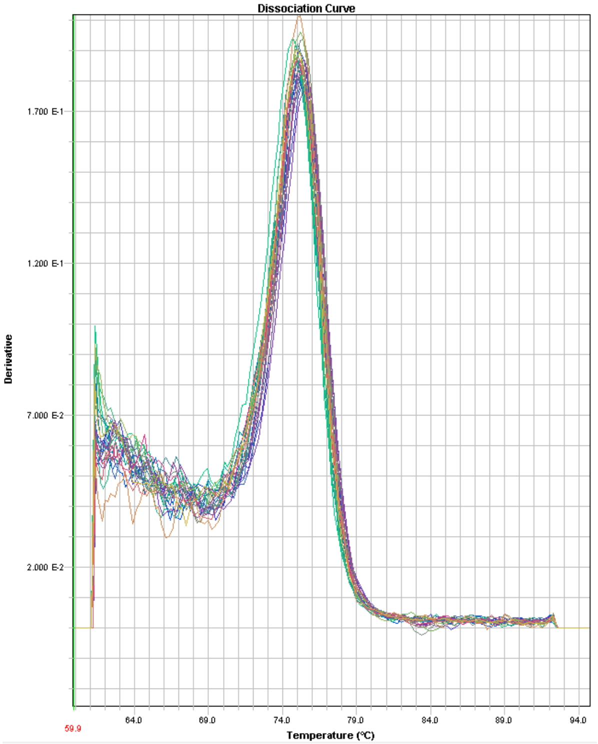

viR09 - In vitro
Mock-infected group

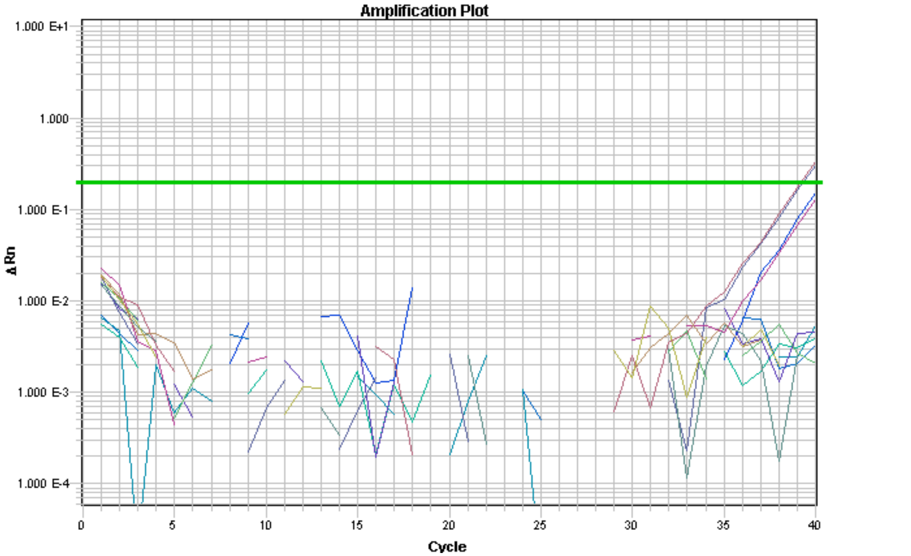

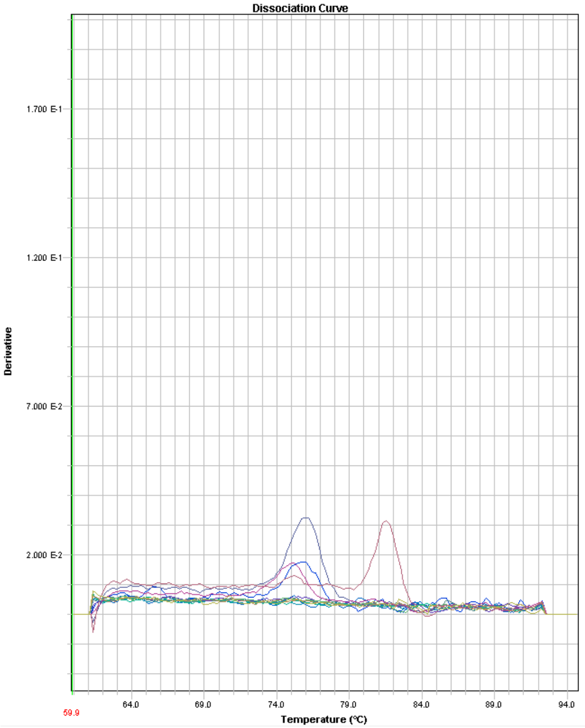

Supplement: Dataset S3 — Amplification and melting curves of qPCR in vitro samples. (PDF) [file pone.0086965.s010.pdf]
