# Supplementary material for: The Role of Viral and Host MicroRNAs in the Aujeszky’s Disease Virus during the Infection Process
Source: PLoS One. 2014 Jan 24;9(1):e86965. doi: 10.1371/journal.pone.0086965 (PMC3901728; doi:10.1371/journal.pone.0086965)

viR02 - In vivo
NIA-3 group
38TG and 31OB samples (RT1)

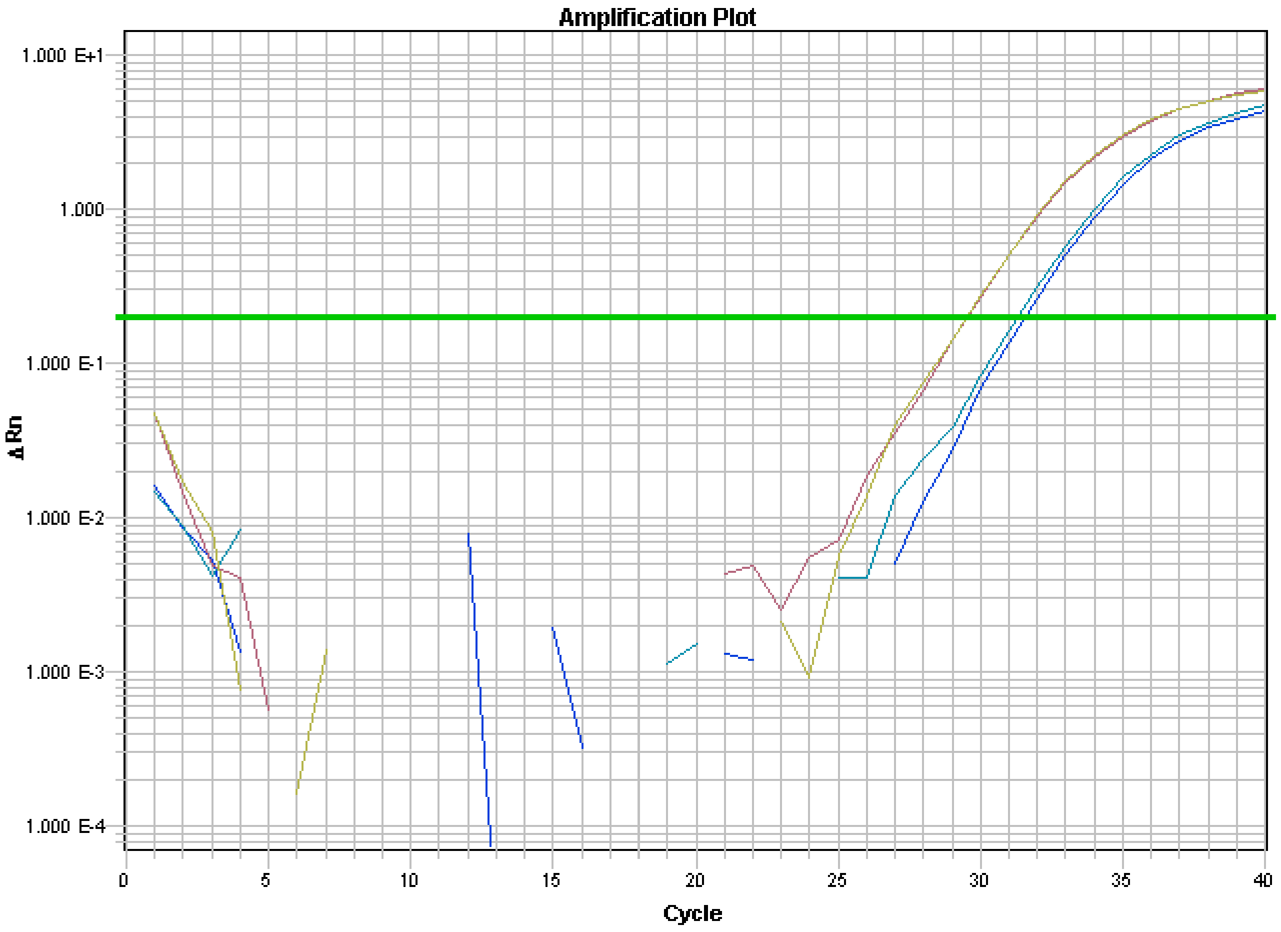

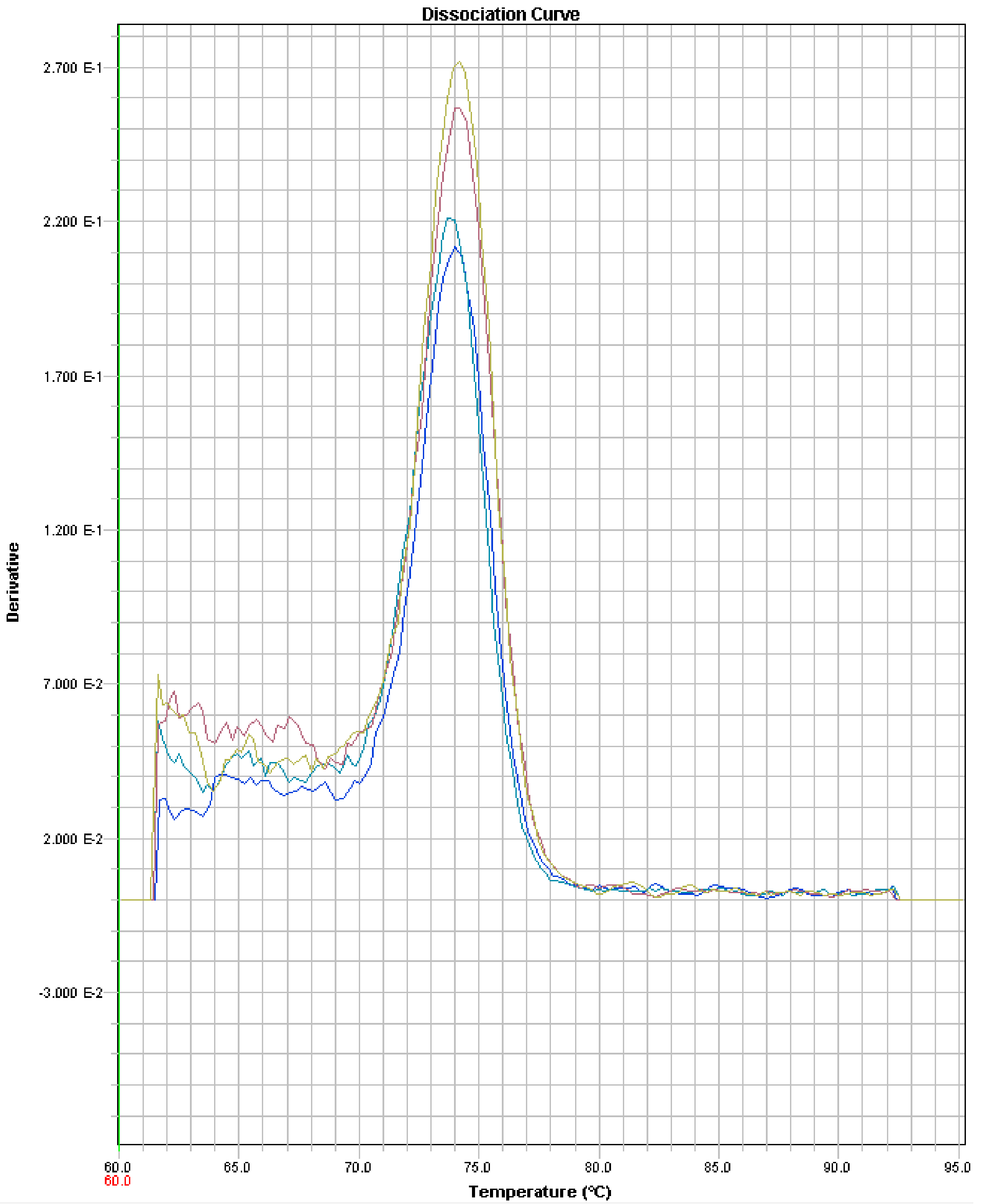

viR04 - In vivo
NIA-3 group
38OB and 41OB samples (RT1)

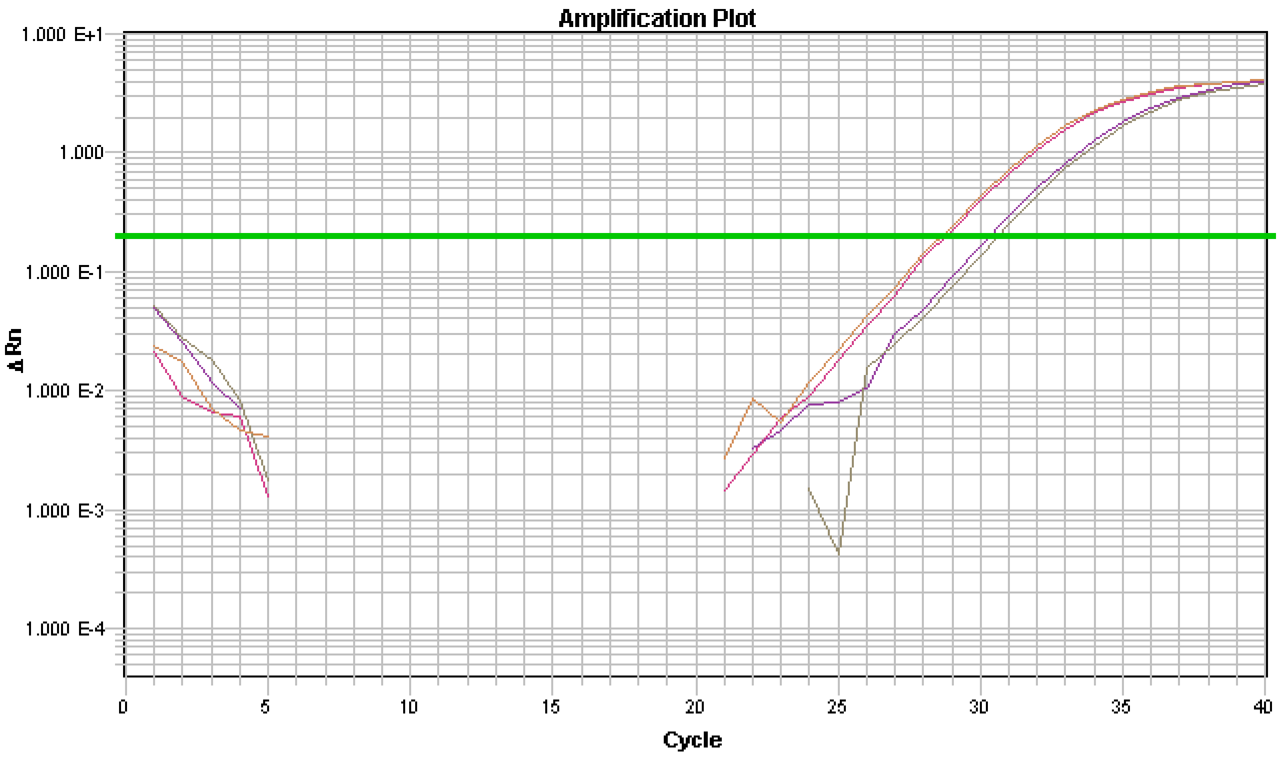

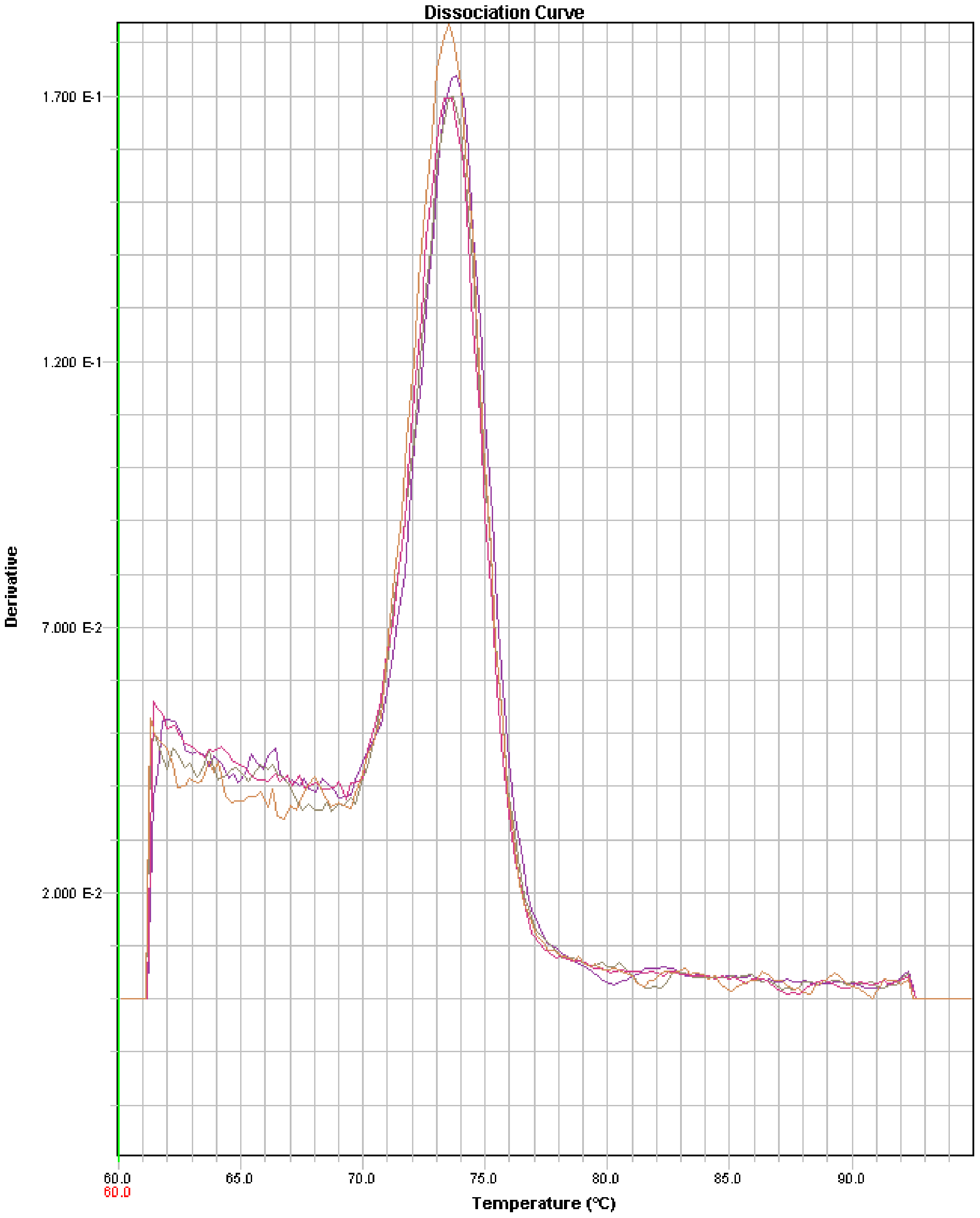

viR06 - In vivo
NIA-3 group
38OB and 41OB samples (RT1)

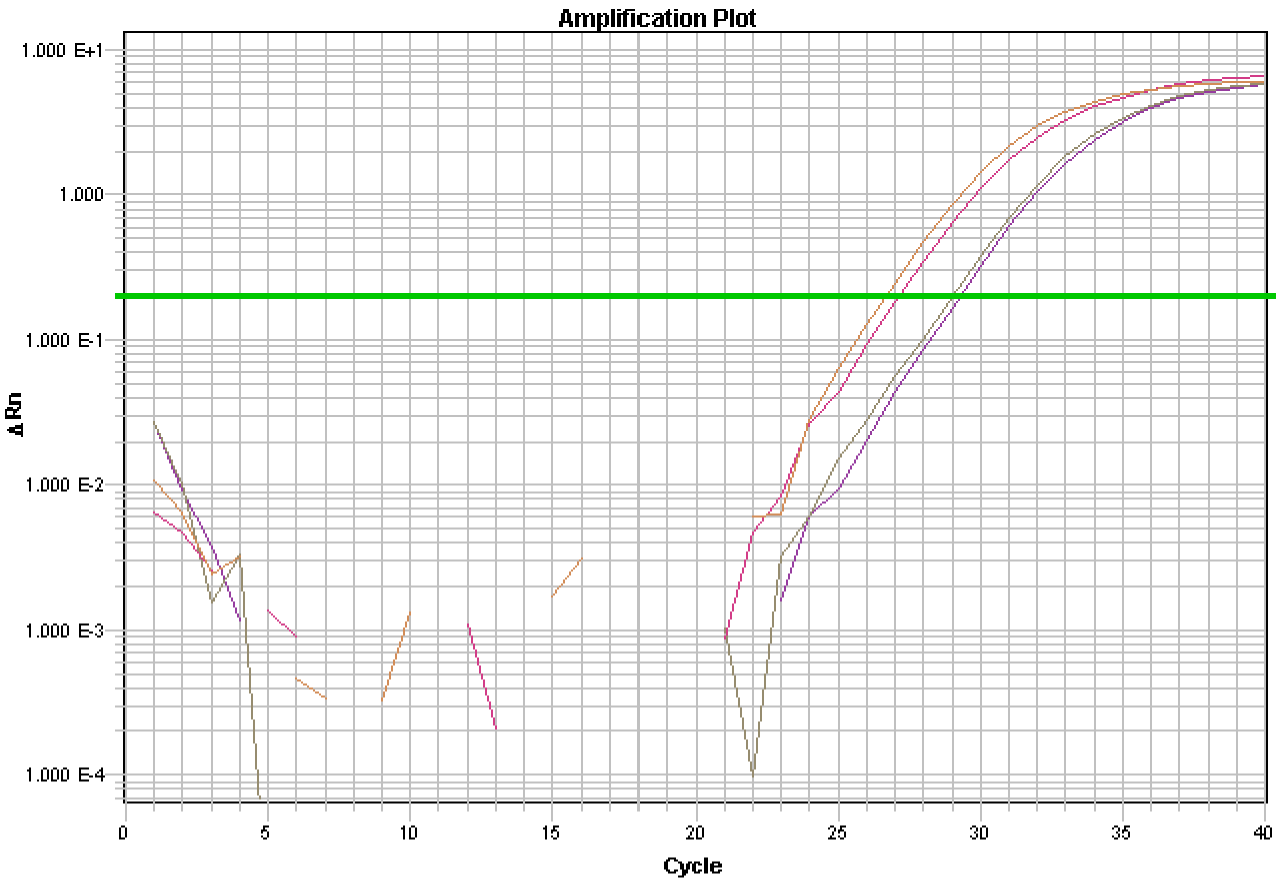

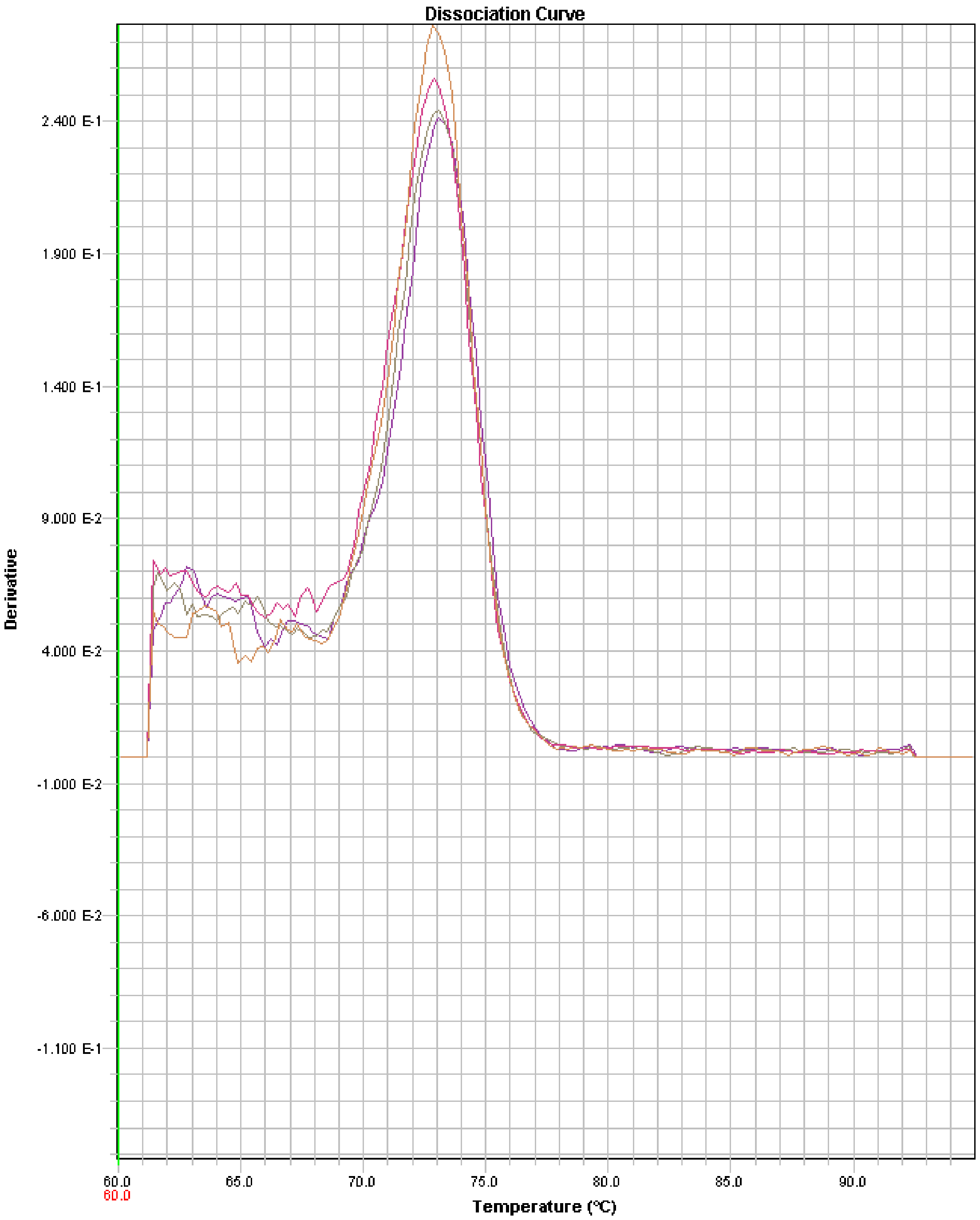

viR08 - In vivo
NIA-3 group
38OB and 41OB samples (RT2)

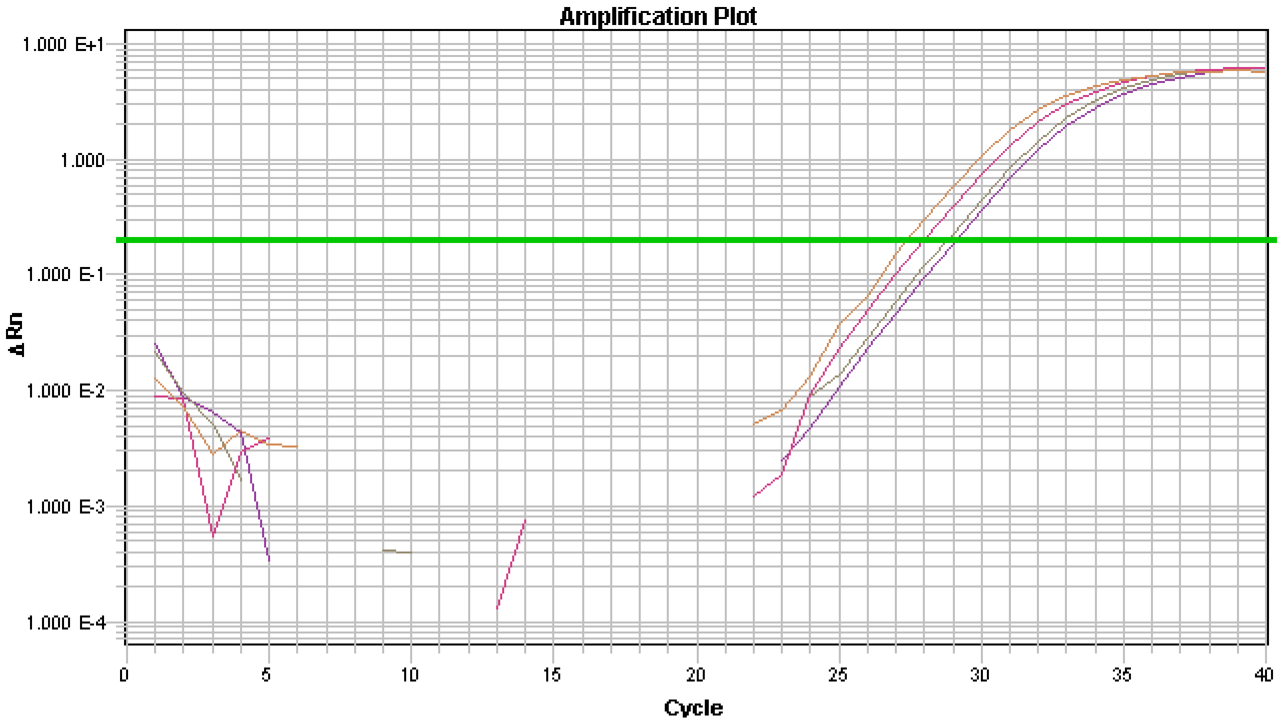

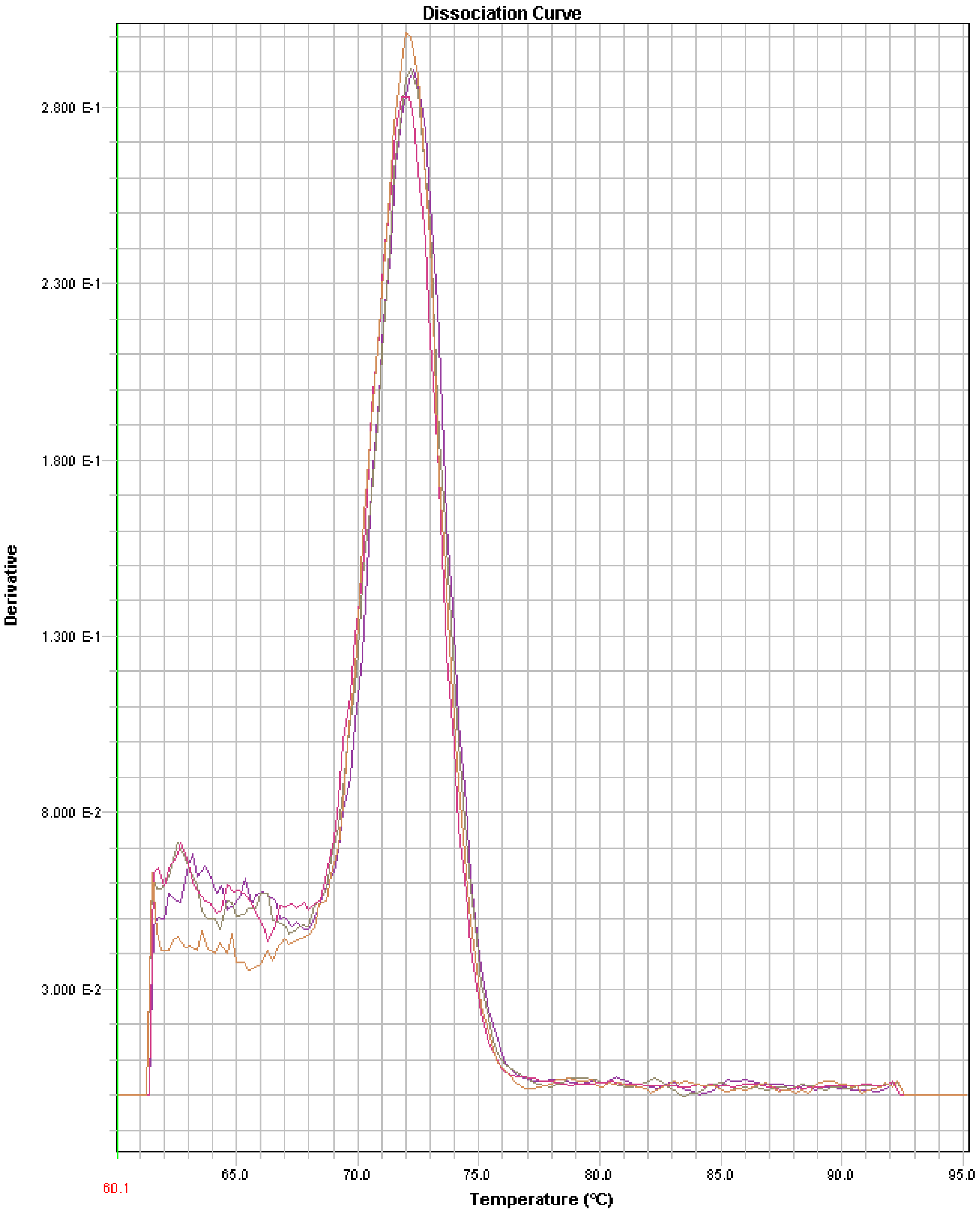

viR09 - In vivo
NIA-3 group
31TG and 39TG samples (RT2)

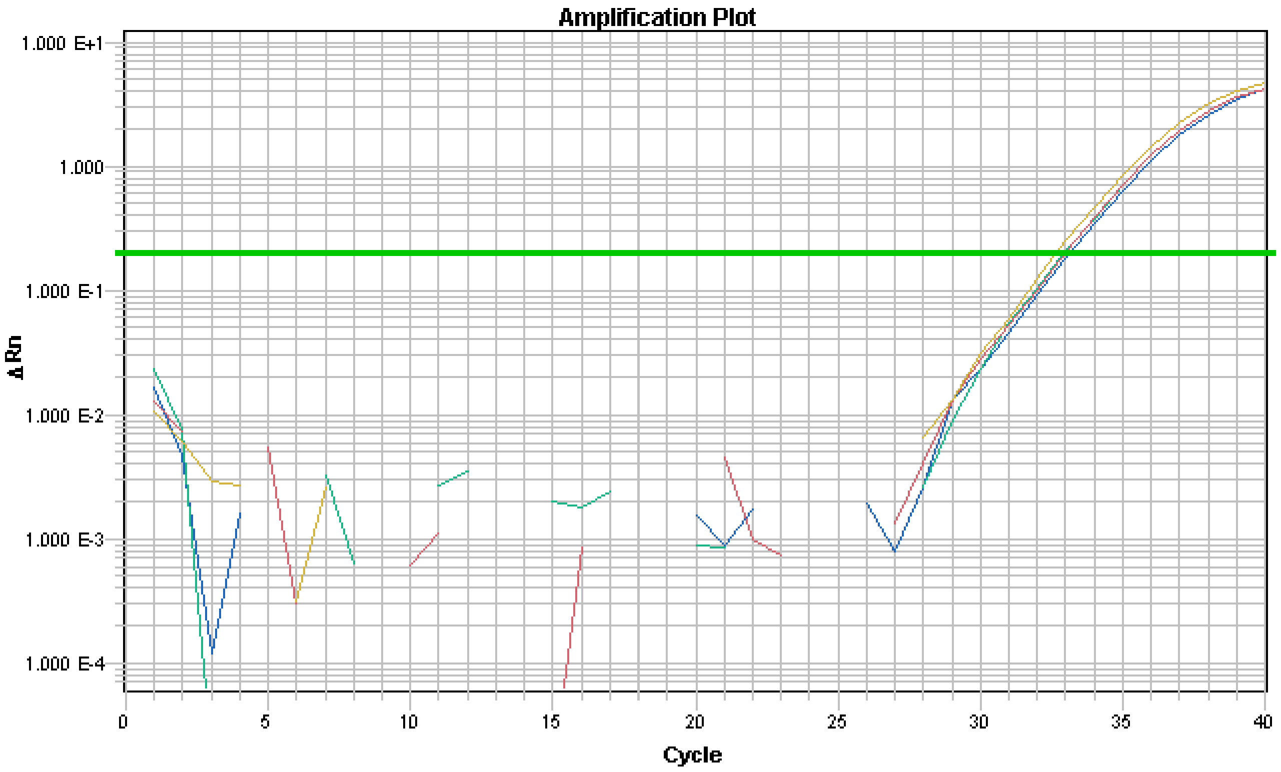

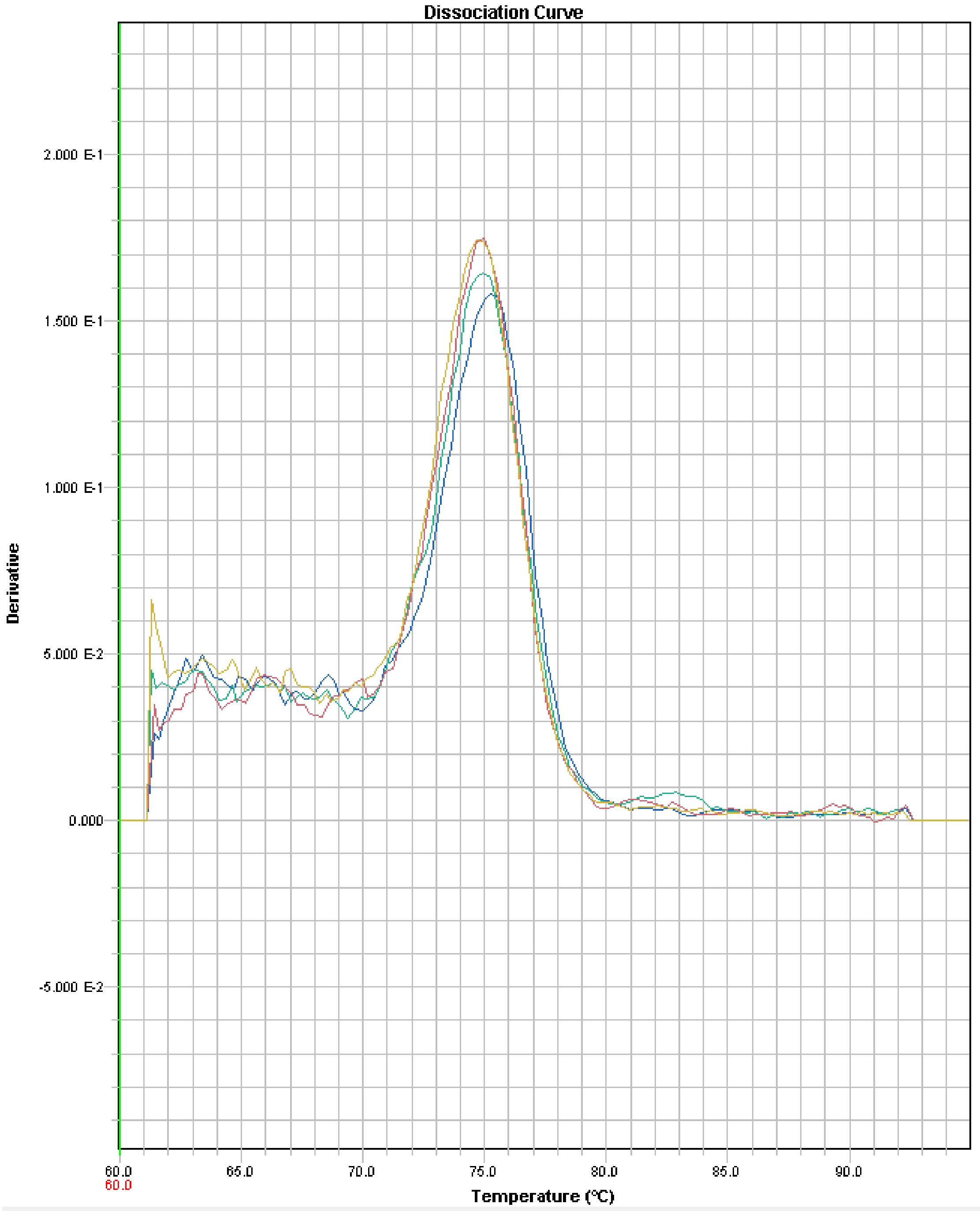

viR11 - In vivo
NIA-3 group
38BO and 41BO samples (RT2)

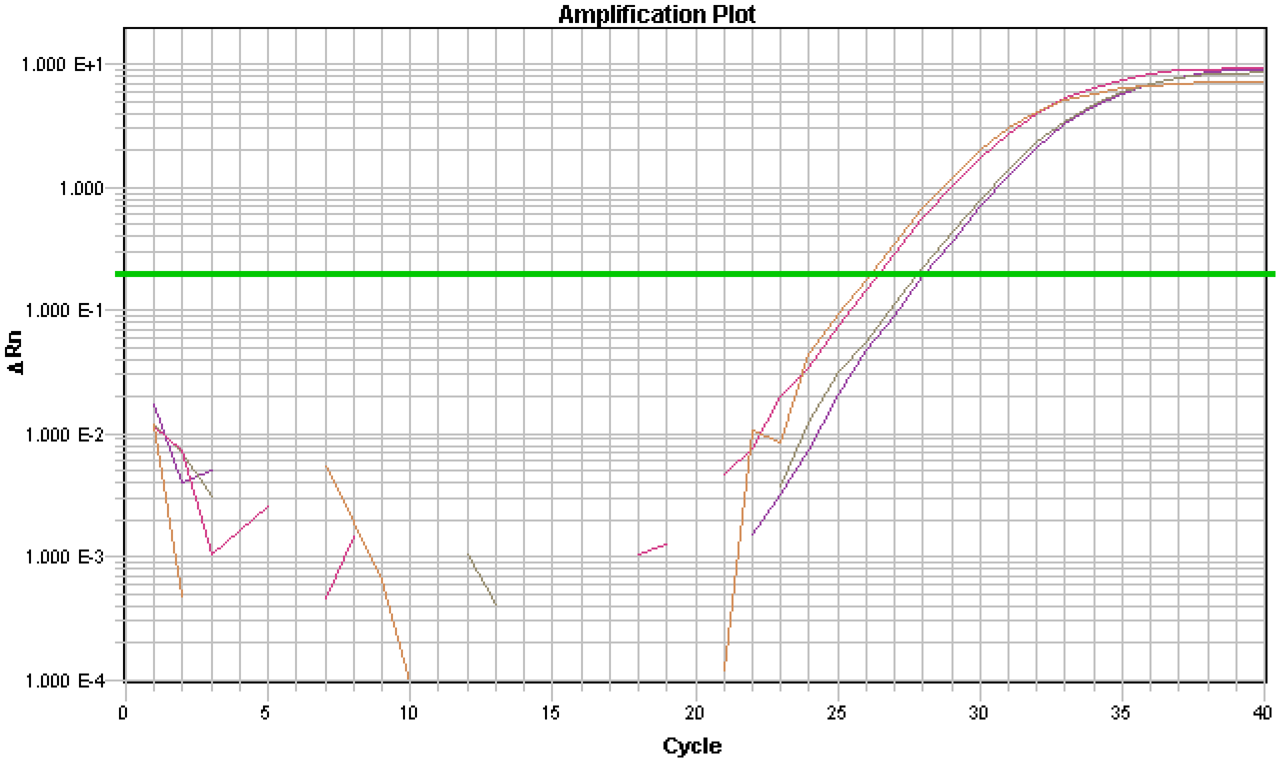

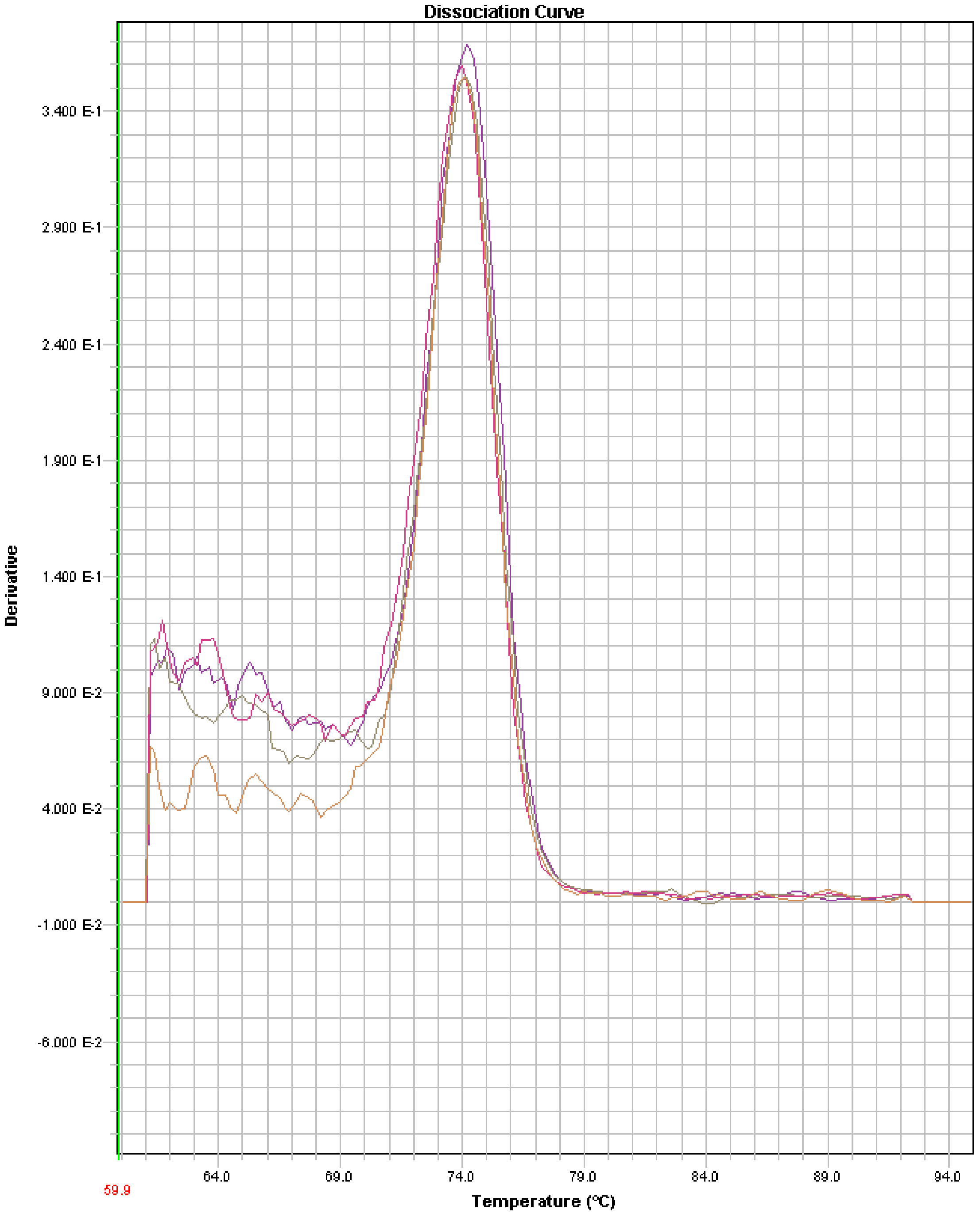

Supplement: Dataset S4 — Amplification and melting curves of qPCR in vivo samples. (PDF) [file pone.0086965.s011.pdf]
